# A positive feedback loop mediated by Sigma X enhances expression of the streptococcal regulator ComR

**DOI:** 10.1038/s41598-017-04768-5

**Published:** 2017-07-20

**Authors:** Rabia Khan, Roger Junges, Heidi A. Åmdal, Tsute Chen, Donald A. Morrison, Fernanda C. Petersen

**Affiliations:** 1 Department of Oral Biology, Faculty of Dentistry, University of Oslo, Oslo, Norway; 2 Department of Microbiology, The Forsyth Institute, Cambridge, USA; 3 Department of Biological Sciences, College of Liberal Arts and Sciences, University of Illinois at Chicago, Chicago, USA

## Abstract

Natural transformation is used by bacteria to take up DNA from their surroundings and incorporate it into their genomes. Streptococci do so during a transient period of competence, triggered by pheromones that they produce, secrete and sense under conditions influenced by the environment. In *Streptococcus mutans*, *Streptococcus suis*, and species of the bovis, salivarius and pyogenic groups of streptococci, the pheromone XIP is sensed by the intra-cellular regulator ComR, that in turn activates the transcription of *comS*, encoding the XIP precursor, and of *sigX*, encoding the only known alternative sigma factor in streptococci. Although induction of *comR* during competence has been known for more than fifteen years, the mechanism regulating its expression remains unidentified. By a combination of directional RNA-sequencing, optimal competence conditions, stepwise deletions and marker-less genome editing, we found that SigX is the missing link in overproduction of ComR. In the absence of *comR* induction, both *sigX* expression and transformation were significantly reduced. Placing *comR* and *comS* transcripts under the control of different regulators so as to form two interlocked positive feedback circuits may enable *S. mutans* to fine-tune the kinetics and magnitude of the competence response according to their need.

## Introduction

Natural competence for genetic transformation is a survival strategy that enables streptococci to attack their siblings, take up the released DNA or other naked DNA in their surroundings, and incorporate it into their genomes. Most of the effector genes are conserved in streptococci, and are under the control of the alternative sigma factor SigX, the master regulator of competence^1–4^. They comprise a set of 27 to 30 genes grouped into the core SigX regulon, showing a predominance of transformation functions, and accessory genes that depend on SigX for expression in species-specific patterns. Expression of SigX in a streptococcal population is triggered by communication signals that are peptide pheromones, produced as precursor molecules. The peptides are later processed and exported, and then sensed either by surface receptors of two-component regulatory systems or by intra-cellular regulators^5–9^.

Surface receptors involved in streptococcal competence that belong to the ComCDE class are found in *S. pneumoniae* and other members of the mitis and anginosus groups of streptococci^5, 10^. In this class, the competence stimulating peptide (CSP) pheromone encoded by *comC* binds to the membrane histidine kinase ComD, resulting in auto-phosphorylation and activation of the cognate response regulator ComE. The modified ComE then activates the promoters of both *sigX* and *comCDE*, inducing their transcription. In this way, the pheromone not only triggers the expression of the master regulator, but creates a positive feedback loop that strongly amplifies the signal.

The second type of competence pheromone system belongs﻿ to the ComRS class, found in *Streptococcus mutans*, *Streptococcus suis*, and in the salivarius, bovis, and pyogenic groups of streptococci^1, 6, 7, 11–14^. The pheromone in this class, a peptide encoded by *comS* known as XIP (SigX-inducing peptide), is imported by oligopeptide permeases. Once inside the cells the pheromone binds to and activates the intracellular regulator ComR. The ComRS complex, in analogy to the activated ComE regulator described above, activates the promoters of *sigX* and of the pheromone structural gene, *comS*. However, in contrast to the ComCDE class, which amplifies the signal by inducing the simultaneous expression of the pheromone and its regulator, in most of the ComRS class only the pheromone expression is induced. Yet, overexpression ﻿﻿of *com﻿R* in *S. mutans* has a strong positive effect on transformation efficiency^7^, while in the salivarius group, low levels of *comR* expression are associated with low transformation efficiency in a range of wild-type strains^15^. Thus, although mechanisms for *comR* regulation remain unknown, accumulated evidence indicates that control of *comR* expression would provide an additional way for streptococci to fine tune the competence response.

Variable levels of *comR* induction are actually observed among the genes up-regulated during *S. mutans* competence^16–20^. Interestingly, the 3′ portion of the gene immediately upstream of *comR* was recently found to be up-regulated upon stimulation of competence, possibly by SigX^2^. We hypothesized that this region may have a role in competence, mediated by either a putative peptide annotated as smut_c_1_105 at the Human Oral Microbiome Database (HOMD; http://www.homd.org), or by acting as an intragenic start site for transcription of *comR*. Using a combination of directional RNA-sequencing, optimal competence conditions, stepwise deletions and direct genome editing of the putative peptide gene and of the SigX-box upstream of *comR*, the present study shows that SigX, the master regulator of competence, is the missing link in overproduction of ComR during development of competence in *S. mutans*.

## Results

### Up-regulation of transcription of the intergenic region between SMU.60 and *comR* during competence is abolished in *comS*, *comR*, and *sigX* mutants

**Figure 1:**
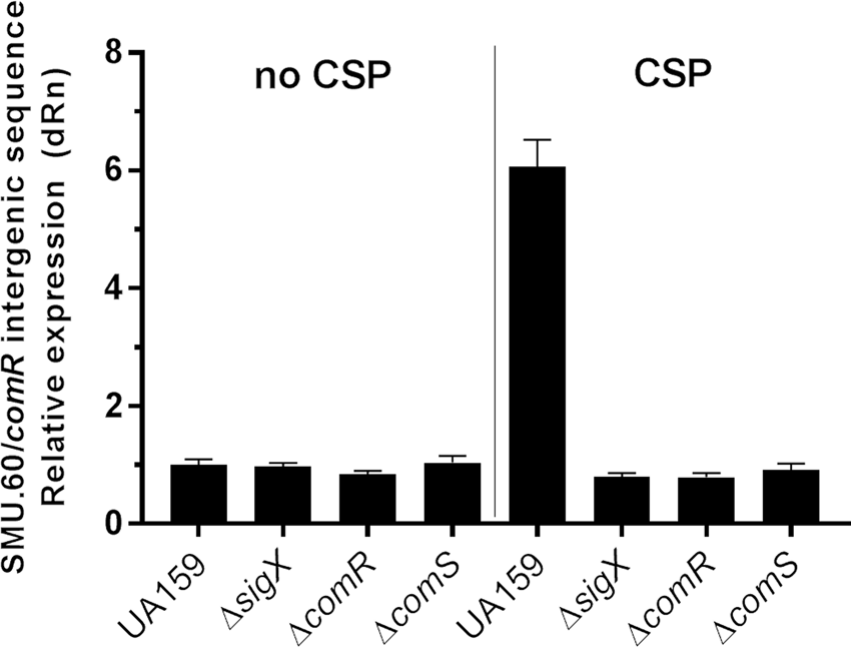
Up-regulation of the intergenic region between SMU.60 and *comR* is a late competence response. Relative expression of the region located between SMU.60 and *comR* in the *S. mutans* wild type UA159 and in the isogenic mutants SM061 (Δ*sigX*), SM066 (Δ*comR*), and SM089 (Δ*comS*). The strains were grown in TSB in the presence or absence of CSP. The real-time PCR data was normalized to the expression values in UA159 with no CSP. The results show mean values and standard errors for 3 replicates.

A recent transcription study suggested that an apparent intragenic SigX-box in SMU.60, which is located upstream of *comR*, may drive expression of a transcript that extends to the intergenic region (IGR) between SMU.60 and *comR* ^2^. To characterize transcripts extending upstream of *comR* more thoroughly, we performed real-time PCR with primers designed to amplify within the intergenic region between SMU.60 and *comR*. Using cultures optimized for CSP induction of competence2﻿1, the results showed that this region is up-regulated approximately 6-fold in UA159 (Fig. 1). Because ComRS is the complex that induces *sigX* transcription, and a predicted SigX box is located within the SMU.60 ORF, we repeated the analysis for mutants deleted for the *comS*, *comR*, or *sigX* genes; induction of transcription of the IGR was abolished in every case (Fig. 1). The requirement for all three genes for this transcriptional response suggested that SigX may be the proximal regulator of the transcript initiated within SMU.60.

### RNA-seq analysis maps the start of the induced *comR* transcript to an intragenic region in SMU.60 and suggests that it extends to the full length of *comR*

**Figure 2:**
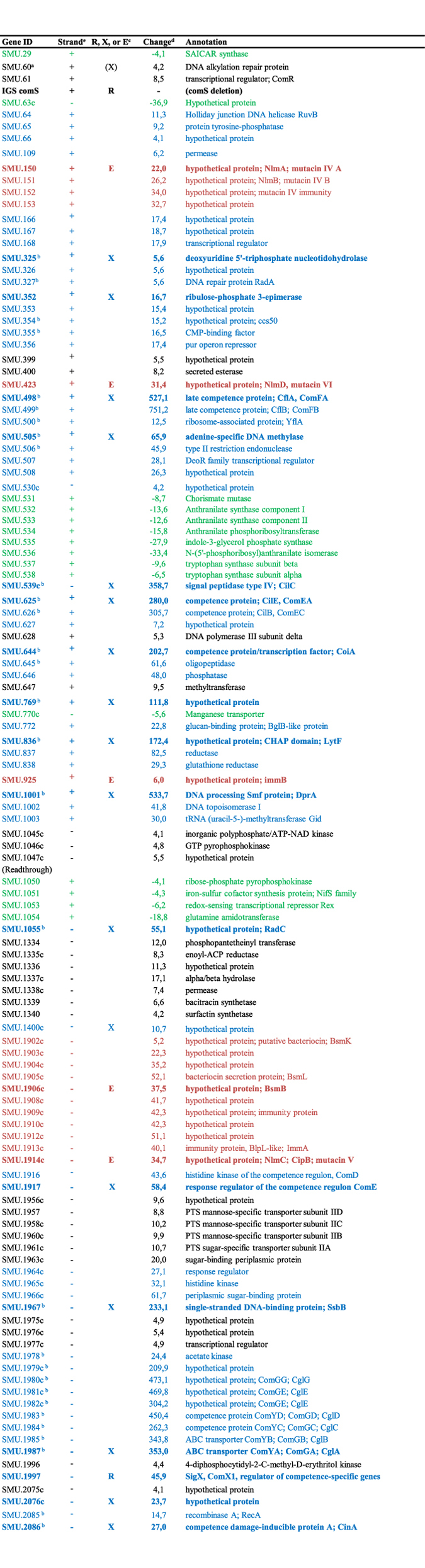
Genes differentially regulated in response to XIP in the *S. mutans comS* deletion mutant (>4 fold; p < 0.05; > 8000 mean reads). Genes belonging to the same transcript are grouped together. Induced genes by XIP that are specific to the early (red) and late (blue) responses to CSP are indicated by the corresponding colors^2^. Downregulated genes (green). ^a^SMU.60 was up-regulated only in its 3′-terminal region preceding *comR*. ^b^Pan-streptococcal genes belonging to the SigX regulon. ^c^First genes in the transcripts regulated by ComR (R), SigX (X), and ComE (E). ^d^Mean fold change in transcript expression from 3 independent biological experiments comparing 120 min XIP treated to untreated parallel cultures. ^e^Plus and minus strand for the ORF are presented, since some of the annotated genes in UA159 (accession AE014133) in the complementary strand (- strand) do not have the “c” designation after the gene number.

**Figure 3:**
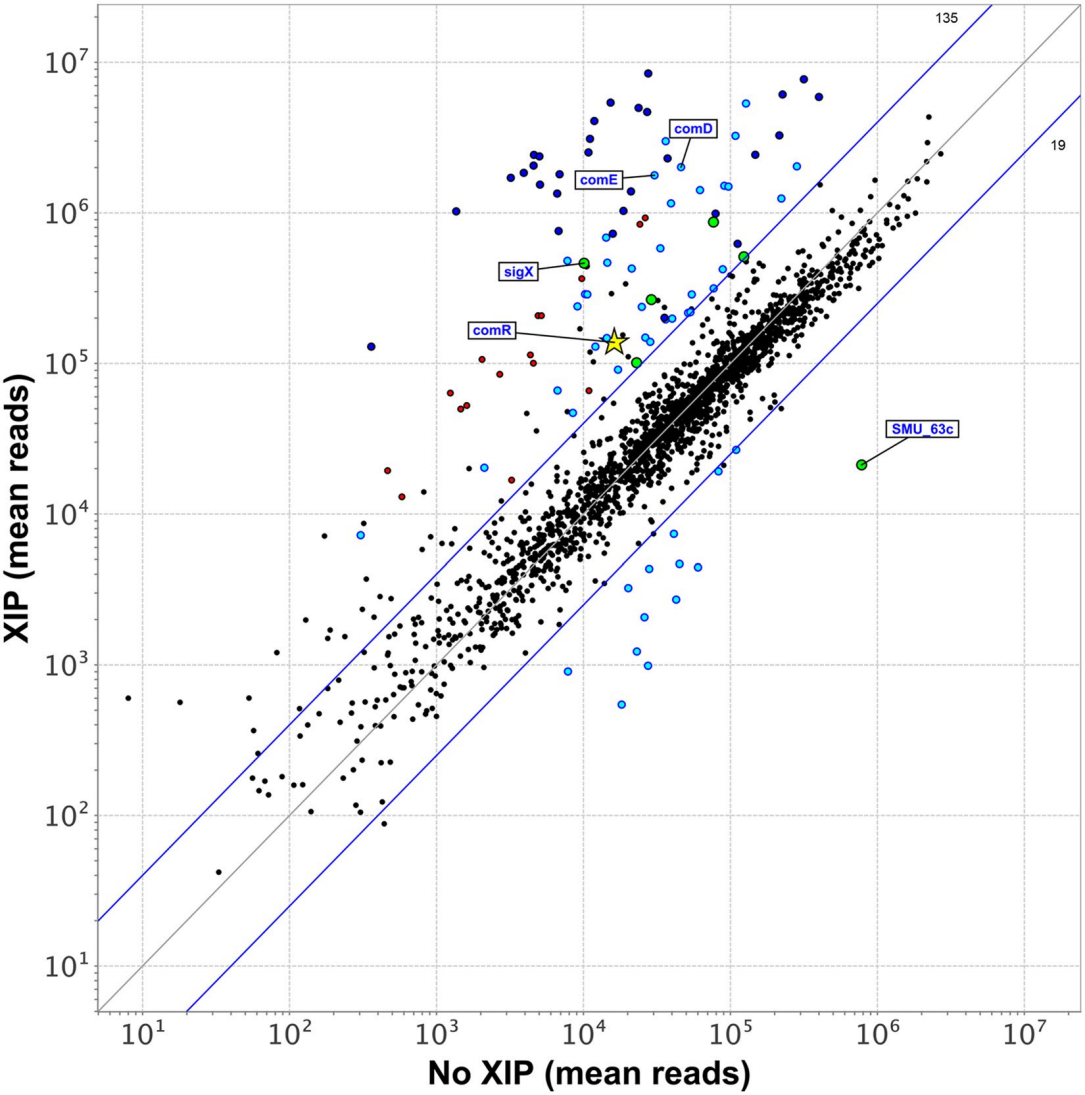
Transcriptome scatter-plot comparing *S*. *mutans* grown in the presence or absence of the XIP pheromone in CDM. Normalized mean reads (log scale) for all annotated *S. mutans* open reading frames (mean values are from three independent experiments). ORFs of the ComR (green), ComED (red), and SigX (light and da﻿rk ﻿blue) regulons, as shown in Fig. 2. The ORFs of the SigX regulon comprising the pan-streptococcal SigX regulon are in dark blue. The yellow star corresponds to the *comR* gene. Four-fold change marker (blue line; 135 ORFs > 4, 19 ORFs < 4).

**Figure 4:**
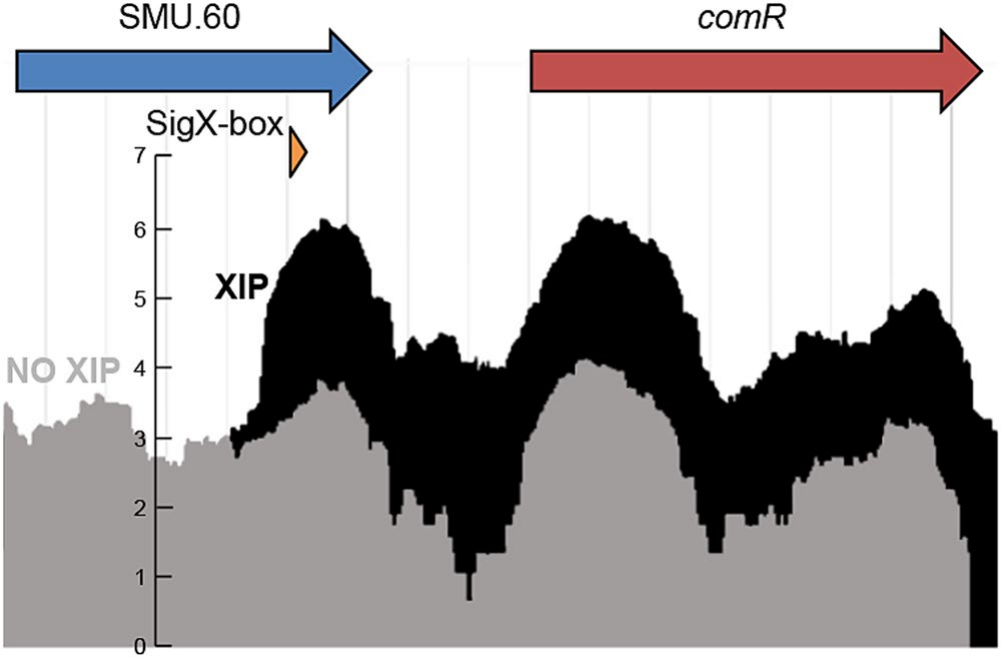
Transcriptome map showing expression of *comR* and its upstream region in *S. mutans* Δ*comS* grown in the presence (black) or absence (light gray) of XIP in chemically defined medium. The images are superimposed to illustrate the differences in expression between the two conditions, starting next to the predicted intragenic SigX box. The axis on the left represent the log_2_ average of read counts of normalized individual duplicated samples for each condition, as detailed in material and methods.

To obtain a precise map of the transcripts induced by XIP, including the SMU.60*/comR/comS* region, we adopted an optimized protocol for *S. mutans* competence development that includes optimal exposure time, dilution rates, and growth in CDM, as previously described^21^. To reduce possible influences from endogenous XIP production, the experiment used a *comS* deletion mutant. The comparisons revealed robust up-regulation of all the 30 core genes of the pan-streptococcal SigX regulon by XIP (Fig. 2 and Fig. 3). The transcriptome map showing the alignment of all transcripts to the reference sequenced genome of UA159 can be visualized at the Microbial Transcriptome Database (MTD; http://bioinformatics.forsyth.org/mtd/?name=RNAseq_smu065&toc=1) and fold changes for the genes in the entire genome are presented in Supplementary Table S1. The transcriptome map obtained with the *comS* mutant allowed us to investigate the up-regulation of the SMU.60-*comR* region at single-base resolution. We found that induced transcription of this locus was indeed initiated within and in the same direction as SMU.60, and that the ~nine-fold elevated transcription included the full length of *comR* (Fig. 4), whereas there was little or no transcription from the complementary strand.

All the genes of the ComE, ComR, and SigX regulons previously mapped in a comprehensive study of the competence responses in *S. mutans* ^2^ were identified here (Fig. 2). The surplus of genes that were up-regulated in the present study, except for the locus between SMU.1332 to 1340, were almost all co-transcribed with the genes of one of the three regulons (Fig. 2 and Fig. 3). Transcripts induced in the anti-sense direction are presented in the maps depicted in Supplementary Figure S1. Overall, the transcriptome obtained here by RNA-seq and XIP as the inducing pheromone showed induction of basically the same sets of transcripts as those found in the previous comprehensive study using a tiling array and CSP, with few exceptions.

### Stepwise deletions link the transcription of the intragenic region upstream of *comR* to expression of *sigX*, and point to a second predicted promoter upstream of *comR*

**Figure 5:**
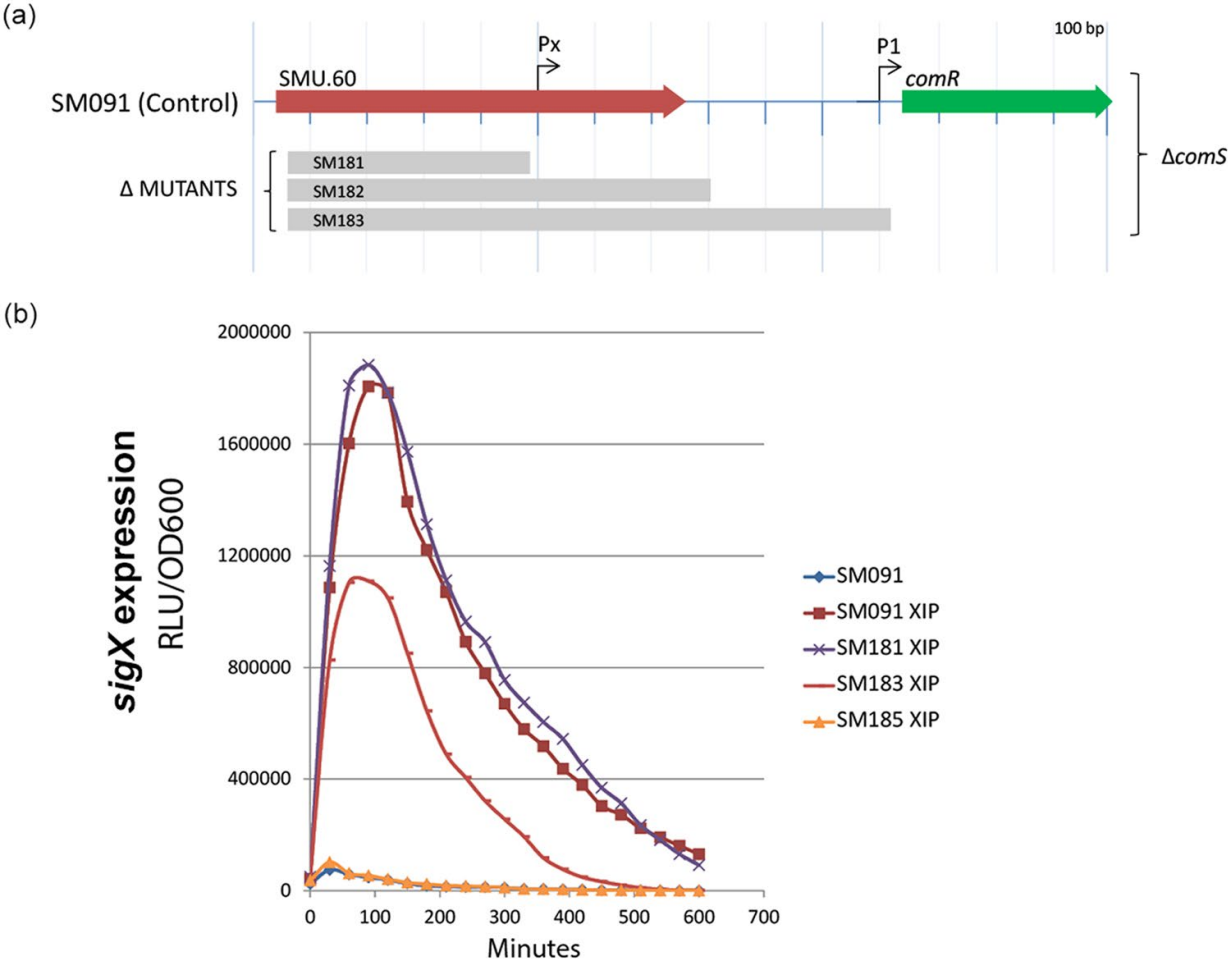
Effect of stepwise deletions upstream of *comR* on *sigX* expression. (**a**) Map of the SMU.60 intragenic region exhibiting a putative promoter with a σx-box (Px; AACGAATA), a predicted *comR* promoter close to the start of the gene (P1; palindrome sequence TAGTAAATTTACTA), and the regions deleted in the SigX-luciferase reporter mutants SM181, SM183, and SM185, respectively. All the strains were constructed in a Δ*comS* background. (**b**) *sigX* expression in response to XIP in the wild type background (SM091), and in the mutants SM181, SM183, and SM186. Luciferase activity was measured in the presence of luciferin, and is presented as relative light units (RLU) per OD600. The results are mean values of 3 experimental replicates, and are representative of 4 independent experiments.

To investigate the involvement of the 3′ portion of SMU.60 in regulation of competence, we used a stepwise deletion strategy to construct (1) a mutant that lacked the 5′ part of the SMU.60, but in which the intragenic SigX box was preserved (mutant SM181), (2) a mutant that lacked the entire SMU.60, including the intragenic SigX box (mutant SM183), and (3) a mutant that lacked both the entire gene SMU.60, the SigX-box, and in addition a *comR* upstream region with a palindromic sequence close to the start of the *comR* ORF (SM185) (Fig. 5). All the mutants were constructed in a *comS* deletion background that included a SigX-luciferase reporter.

The expression of *sigX* upon stimulation with XIP in mutants SM181, SM183, and SM185 was compared to that in the SM091 control using the luminescence assay. As expected, without XIP no *sigX* expression was observed in any of the strains (for simplicity, the results are only shown for the SM091 in Fig. 5). In the presence of XIP, induction of *sigX* was reduced by approximately 40% in SM183, and by 100% in SM185, while no significant differences were observed between SM181 and the control SM091 strain (Fig. 5). The results thus indicate that an intact DNA alkylation repair protein encoded by SMU.60 is not required for *sigX* induction by XIP. In contrast, induced expression of *sigX* was reduced two-fold by deleting the entire SMU.60 orf, and was completely abolished by extending the deletion nearly to *comR*. This pattern is consistent with expression of *comR* being absolutely dependent on a proximal promoter (P1) and partially dependent on an additional promoter (Px) located within SMU.60 near the apparent SigX-box.

### Inactivation of the intragenic SigX box by direct genome editing reduces *sigX* expression and induced expression of *comR*, and reduces transformation in response to XIP

**Figure 6:**
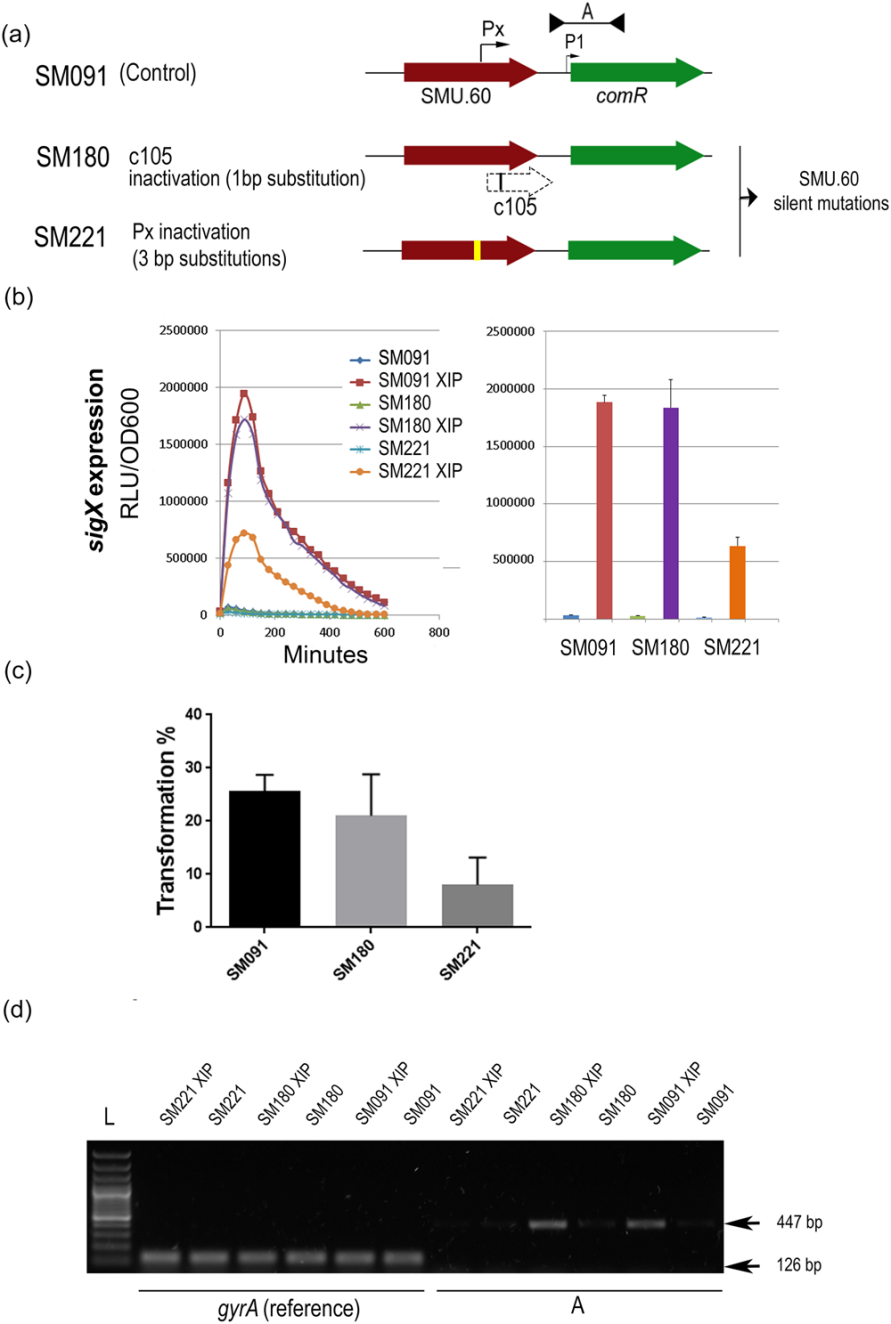
Effect of single base mutations upstream of *comR* on *sigX* expression, competence, and *comR* transcript length. (**a**) Map of the SMU.60 intragenic region with a putative promoter containing a σx-box (Px) and the downstream predicted *comR* promoter closest to the start of the *comR* gene (P1). The A region corresponds to the PCR fragment amplified by the primer pair FP785 and FP1200. The *S. mutans* strains used were all Δ*comS*, SigX-luciferase reporters: SM091, wild type background; SM221, mutant with an inactivated σx-box (yellow vertical box); SM180, mutant with a stop codon (black vertical line) within a possible alternative open reading frame (ORF; dashed arrow) overlapping with part of the SMU.60 gene. (**b**) *sigX* expression with and without addition of XIP in CDM. Luciferase activity measured in the presence of luciferin is presented as relative light units (RLU) per OD600. Left panel: Kinetic results representative of three independent experiments. Right panel: Average from three independent experiments at 100 min. Bars represent standard deviation. (**c**) Transformation efficiency using the 6,3 Kb amplicon aRJ02 as donor DNA. The results are averages of 3 independent experiments. Bars represent standard deviations. (**d**) Gel image showing cDNA amplification in the A region of the WT and SM180 in the presence of XIP. The A transcript was not detected in SM221. Primers FP785 and FP1200 were used to amplify A (447 bp), and FP299 and FP300 to amplify *gyrA* (126 bp; reference). L; 1Kb Plus DNA ladder (Invitrogen).

To investigate the intra-SMU.60 SigX-box site more precisely, we chose to inactivate the SigX box in SMU.60 without affecting its expression, by using primers for overlapping PCR in which bases A, C, and G in positions 2, 3, and 8 in the SigX box (T**AC**GAAA**A**) were replaced by G, A, and G (T**GA**GAAA**G**). The three single base substitutions introduced only silent mutations in SMU.60 (SM221 mutant). Both expression of *sigX* and transformation were reduced in SM221 by 50%. We then compared the expression of the transcript initiated at the putative SigX box in SM221 with the expression in the control strain SM091. Induction of the transcript by XIP in this mutant, as measured by reverse-trancriptase PCR, was severely reduced (Fig. 6). We conclude that the SigX box is essential for the increase in *comR* expression observed both here and in previous studies^16, 19, 20, 22^.

### Introduction of a stop codon in a putative open reading frame downstream of the intragenic SigX box has no significant effect on the expression of *sigX* and *comR*, or on competence

Annotation of the genome at HOMD (http://www.homd.org/) indicates the presence of a short ORF (c105) immediately downstream of the SMU.60 intragenic SigX-box. To investigate whether expression of a protein encoded by this ORF could be responsible for the linkage of competence to the presence of the SigX-box, we constructed a mutant with a single-base substitution that created both a silent mutation in SMU.60 and a stop codon at the start of the putative ORF (SM180). This mutant was not affected in *sigX* expression, transformation, or the formation of the extended transcript (Fig. 6). This indicates that if this protein is produced at all, it has no effect on competence.

### Conservation of the SMU.60 intragenic SigX-box in *S. mutans*

**Figure 7:**
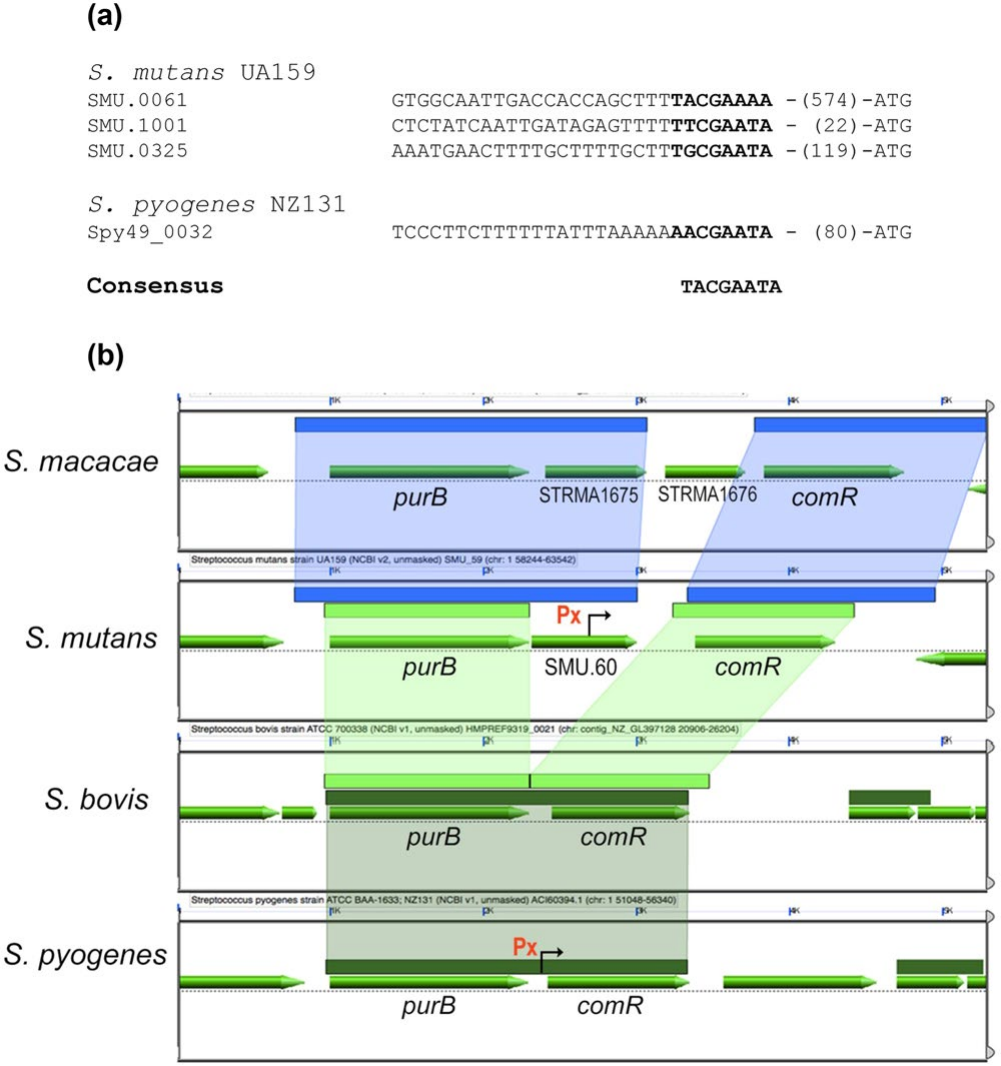
Alignment of DNA sequences upstream of *comR* in *S. mutans* UA159, *S. pyogenes* NZ131, *S. bovis* ATCC700338, and *S. macacae* NCTC11558. (**a**) In bold are the SigX-boxes in the Px promoters for *comR* in *S. mutans* and *S. pyogenes*. No apparent SigX-box was identified in *S. macacae* or *S. bovis*. The SigX-box regions of the most induced locus, *dprA* (SMU.1001), and of the least induced locus, *dut/radA* (SMU.0325) are also shown. (**b**) Synteny in the *comR* region as determined by CoGe:GEvo, using the conserved *purB* gene as a reference (https://genomevolution.org/coge). Mutans group; Synteny in the *comR* region was found only *in S. macacae*, as determined by CoGe:GEvo, using the conserved *purB* gene as a reference.

Analysis of all sequenced genomes of *S. mutans* using BLAST at COGE (https://genomevolution.org/coge) revealed that the SMU.60 intragenic SigX-box and the position of the *comR* gene is similar in all of them, indicating that the regulation of *comR* by SigX is widely conserved in *S. mutans*. Other members of the mutans group of streptococci are *Streptococcus sobrinus*, *Streptococcus criceti*, *Streptococcus downei*, and *Streptococcus macacae*, but *comRS* genes among these members were found only in *S. macacae*. The arrangement of the locus in *S. macacae* differs, however, from that in *S. mutans*, as there is an additional gene positioned between SMU.60 and *comR*, and that there is no apparent SigX-box in the SMU.60 intra-genic region. Among the 4 other streptococcal groups that use ComRS for competence regulation, including salivarius, bovis, *S. suis*, and *S. pyogenes*, only species in the bovis and pyogenic groups had a gene arrangement that resembled that in *S. mutans*, showing a *purB* gene upstream of *comR*. However, SMU.60 homologues are not found in their genomes. Despite the lack of SMU.60, there is a SigX-box signature upstream of *comR* in *S. pyogenes*, but not in *S. bovis*. The presence of the SigX-box only 80 bp upstream of the *comR* start codon suggests that *S. pyogenes* may regulate *comR* expression via SigX as well (Fig. 7).

## Discussion

In bacteria that develop competence transiently, the coordinated up-regulation of dozens of genes is supported by one or more positive feedback loops. In *S. pneumoniae*, for instance, the exported CSP pheromone stimulates its own production via the ComDE two-component signal transduction system. Another example is the ComK transcription activator of the DNA-uptake and recombination machinery in *Bacillus subtilis*, which binds to its promoter to activate its own expression. In *S. mutans*, one positive feedback loop connects SigX to ComE and bacteriocin expression and a second connects XIP to *comS* expression. Both feedback loops are involved in the generation of population heterogeneity sometimes observed during competence in *S. mutans*. Yet, the bi-stability behavior cannot be explained by the two loops alone^23^. The involvement of an additional positive loop involving ComR has been put forward as a possible explanation^24^. However, no mechanism for the regulation of the central competence regulator ComR is yet known, and although increased expression of *comR* during *S. mutans* competence has been reported, the described changes are small^16–20^.

The proximity of the intragenic SMU.60 SigX box to *comR* and *comS* suggested that this region could play a previously unknown role in competence, perhaps in the regulation of *comR*. Using optimized protocols to achieve high levels of competence^21, 25^, the induction of *comR* became more prominent. Nine-fold induction of *comR* was detected in CDM, in contrast to previous reports with results ranging from no induction to 1.4 and 4-fold induction﻿^2, 16–20, 26^. Evaluation of transformation and *sigX* expression in mutants representing stepwise deletions within this segment revealed the regulatory mechanism involved. Marker-less single base substitutions to inactivate the SigX box resulted in a mutant that reproduced the partial competence inhibition found in the SigX box deletion mutant, whereas a base substitution that introduced a stop codon in the putative c105 orf encoding a peptide transcribed from the SigX-box had no effect on competence. Finally, the RNA-seq and RT-PCR results indicating that the transcript initiated at the SigX-box is co-transcribed with *comR*, led to the conclusion that SigX regulates *comR* expression. The discovered mechanism for *comR* regulation is apparently the missing explanation for previous reports on *comR* induction during competence. It may also explain why complementation of a *comR* deletion mutant with a plasmid that incorporates only the proximal promoter of *comR*, and not the SigX-box, does not completely rescue the transformation phenotype observed in the wild type^7^, while expressing the same plasmid in the wild type results in a ten-fold increase in transformation efficiency.

Distal to *comS*, there is an additional set of genes consistently reported as up-regulated during competence that extends from SMU.64 to SMU.66, and occasionally to SMU.68^16–20^. Between *comS* and this set of genes is SMU.63c, which codes for a recently identified amyloid protein induced by both XIP and CSP^27^. The reported induction in gene expression contrasts with a recent microarray study using directly labeled RNA, showing that induction of SMU.63c was restricted to the non-coding strand^2^. The present directional RNA-seq results confirmed this observation, and showed that in the sense-direction, the expression of SMU.63c was actually reduced by more than 35-fold. While the most reasonable explanation for the increase in SMU.63c expression reported in previous studies is the use of methodologies that are not strand specific, it does not account for a recent transcriptome study using a directional approach that showed an increase of SMU.63c in the sense direction^26^. In the absence of a candidate regulator for SMU.63.c, it is reasonable to infer that SMU.63c transcription is induced from the non-coding strand as part of a single transcript that extends all the way to SMU.66 or beyond, depending on the magnitude of competence induction.

Streptococci have a core of 27 to 30 genes that are under the control of the alternative sigma factor SigX^2^. All of them were induced by XIP in the present study, confirming the conservation of the regulon. Two other studies have also investigated the effect of XIP on *S. mutans* competence^20, 26^. In the first, five of the SigX core genes were not induced during competence (*dut*, *radA*, *dpnA*, *pilC*, and *radC*)^20^, but in this study exposure to XIP was for a short period, and under conditions that do not prevent endogenous XIP production. In the other, only *dut* and *radA* were not found to be up-regulated^26^. The *dut* and *radA* genes are transcribed as a single operon that is among the least induced transcripts of the SigX regulon, which may explain why their induction was not detected in the two studies. The fold changes for all genes in this study, as well as in the previous comprehensive study defining the core set of panstreptococcal genes of the SigX regulon^2^, and the recently reported XIP transcriptome^26^ are presented in Supplementary Table S1. All the accessory genes of the SigX regulon, as well as the genes of the ComRS and ComE regulons identified in the comprehensive study mentioned above were also up-regulated in our study. The surpluses of genes induced in the present study were, except for a transcript initiated at SMU.1340, extension of transcripts belonging to one of the three regulons (Fig. 2).

A notable feature of the regulons induced during competence in streptococci is that core genes lie close to the promoters recognized by the main regulators of competence^2^. The majority has a function directly linked to natural transformation. The link to stress responses, on the other hand, is not well understood, and in most cases is related to accessory genes located downstream of the core genes. Among these is the *relQ* ppGpp synthase (SMU.1046c) and *levDEFG* (SMU1957 to SMU.1961), both associated with nutritional stress, and induced by XIP^26^. The present results confirmed their up-regulation and further revealed that they belong to the class of accessory genes which transcription initiates from upstream core genes of the competence system (Supplementary Figure 1). It remains to be determined whether *S. mutans* may have co-opted this arrangement of SigX regulons close to genes that may slow down their growth as part of a stress response, or whether what we observe is an indirect effect caused by variation in terminator presence and efficacy, as previously suggested^2^.

**Figure 8:**
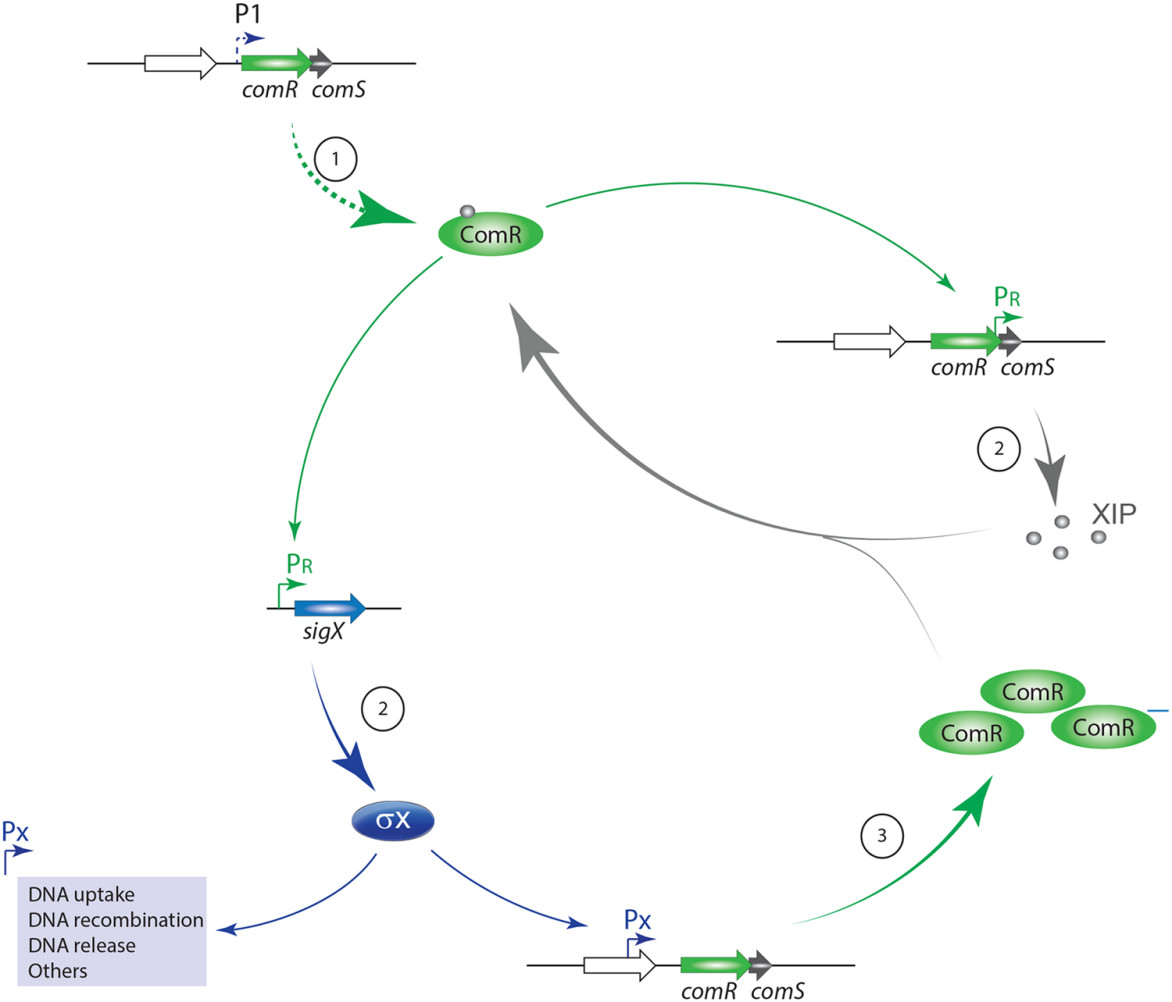
Model of *comR* regulation. (1) Basal levels of ComR and the XIP precursor ComS are kept by transcription of *comRS* from the promoter P1. Imported XIP binds to ComR, forming the ComRS complex. (2) ComRS induces the expression of *sigX* and *comS*, by binding to the PR promoter. This positive feedback loop generates increased amounts of XIP. (3) A second positive feedback loop is mediated by SigX, which, upon recognizing an intragenic SigX-box in the *comR* neighbor gene SMU.60, up-regulates the expression of *comR*. Thus, the system couples expression of *comS*, *comR*, and *sigX* to maximize amounts of the ComR regulator and the XIP cognate pheromone during competence.

Combining the present results with previous literature, leads to a model in which *comR* is expressed at a basal level under non-competence conditions, using a P1 neighbor promoter near a distal palindromic sequence (Fig. 8). During development of competence, XIP or intracellular ComS binds to ComR, initiating the competence response. This leads to elevated transcription of *sigX* and *comS*, and to increased levels of SigX and XIP. SigX in turn drives increased transcription of *comR* from a secondary promoter (Px), to assure that sufficient amounts of the ComR regulator are produced to mount an appropriate response. Placing *comR* and *comS* transcripts under the control of different regulators so as to form two interlocked positive feedback circuits may help *S. mutans* to better adjust the kinetics and magnitude of the competence response. This tight control is particularly important given the fitness cost of competence, which should be high judging by the significant autolysis observed in part of the competent *S. mutans* population^28^, and the dramatic changes in the array of proteins synthesized during competence^29^.

## Materials and Methods

### Bacterial strains and growth conditions

*S. mutans* strain UA159 and isogenic derivatives used in the study are listed in Table 1. Most mutations were inserted into a *p*_*sigX*_-luc and Δ*comS* background. For preparation of pre-cultures and assays, *S. mutans* were cultivated in 5% CO_2_ at 37 °C in tryptic soy broth (TSB) (Oxoid) or chemically defined medium (CDM)^30^. For plates, 1.5% Bacto agar (Becton, Dickinson and Company) was added to the media. When needed, antibiotics were used at the following concentrations: kanamycin (Kan), 500 μg/mL; erythromycin (Erm), 10 μg/mL; spectinomycin (Spc), 500 μg/mL.

**Table 1:** Strains used in the study.

Name	Description	Source
UA159	Wild-type	ATCC 700610
SM061	UA159, but Δ*sigX:: kan*; Kan^R^	This study
SM065	UA159, but Δ*comS*::*spc*; Spc^R^ (from strain MW02 Mashburn-Warren *et al*. (2010)	(Khan *et al*., 2012)
SM066	UA159, but Δ*comR*::*spc*; Spc^R^ (from strain MW05 Mashburn-Warren *et al*. (2010)	This study
SM068	UA159, *p*_*sigX*_-luc; Spc^R^	(Khan *et al*., 2012)
SM089	UA159, but Δ*comS*::*ery*; Erm^R^	(Khan *et al*., 2012)
SM091	SM068, but Δ*comS*::*ery*; Spc^R^ Erm^R^	(Khan *et al*., 2012)
SM166	UA159, but Δ*SMU.60*::*kan*; Kan^R^	This study
SM168	UA159, but Δ*SMU.60_c105*::*kan*; Kan^R^	This study
SM170	UA159, but Δ*SMU.60_*p*comR*::*kan*; Kan^R^	This study
SM180	SM188, but Δ*comS*::*ery*; Spc^R^ Erm^R^	This study
SM181	SM091, but Δ*SMU.60*::*kan*; Kan^R^ Spc^R^ Erm^R^	This study
SM183	SM091, but Δ*SMU.60_c105*::*kan*; Kan^R^ Spc^R^ Erm^R^	This study
SM185	SM091, but Δ*SMU.60_*p*comR*::*kan*; Kan^R^ Spc^R^ Erm^R^	This study
SM188	SM068, but c_105 1-bp substitution; Spc^R^	(Morrison *et al*., 2015)
SM218	UA159, but mutated *cinbox* upstream of c_105; marker-less	This study
SM221	SM091, but mutated *cinbox* upstream of c_105; Spc^R^ Erm^R^	This study

### Synthetic peptides

The *sigX*-inducing peptide (XIP) (NH2-GLDWWSL-COOH; 98% purity; GenScript) was reconstituted with 20 μl dimethyl sulfoxide (DMSO) (Sigma-Aldrich), to which 1 mL distilled water was added to give a final concentration of 10 mM. The mature competence-stimulating peptide (CSP18)^31, 32^ (NH2-SGSLSTFFRLFNRSFTQA-COOH; 98% purity; GenScript) was reconstituted in water to a final concentration of 1 mM. Both peptides were stored in aliquots at −20 °C.

### Construction of mutants with antibiotic resistance markers

A PCR-ligation mutagenesis strategy was used for the construction of four deletion mutants carrying antibiotic markers in the wild-type *S. mutans* strain UA159 (SM061, SM166, SM168 and SM170)^33^. Briefly, flanking regions of ~ 1-kb up- and down-stream of the deleted region were amplified from genomic template DNA, and the kanamycin resistance cassette was amplified from a plasmid. Amplicons carried AscI or FseI restriction sites which were digested with specific restriction enzymes, and ligated with T4 DNA ligase (New England Biolabs) for the creation of the final ligation product. The kanamycin resistance cassette in SM166, SM168 and SM170 was placed in the reverse strand to avoid transcriptional read-through from its promoter into downstream genes. Transformation into *S. mutans* was performed by culturing cells with CSP18 as described previously^21^. Briefly, stock cultures at OD600 0.5 were diluted 1:10 in TSB and an aliquot of a 1 mL was transferred to a microtube. Donor DNA and 18-CSP (50 nM) were added, and the culture was incubated at 37 °C in air for 3 h. At the end of the incubation period, cultures were plated onto selective THB-agar plates. Primers used for the construction of amplicons are listed in Table 2. The mutants were verified for gene deletion by PCR and gel electrophoresis. Genomic DNA from SM166, SM168 and SM170 was transformed into the *ΔcomS* background to create SM181, SM183 and SM185 respectively. SM066 was created by transformation with DNA from previously described strains^7^.

**Table 2:** Primers used in the study.

ID	Sequence	**Restriction site**	**Description**
FP460	CATTCCCTCTTGTTGCCAAT	—	Construction of SM061
FP461	aggcgcgccTGCCGAACACAGCAGTTAAG	AscI	
FP462	aggccggccCTTGGTAGCAGGAGAGCAC	FseI	
FP463	AAAGCACAGCCTGCTTCAAT	—	*Kan marker in the FW strand*
FP001	aggcgcgccGTTTGATTTTTAATG	AscI	
FP068	aggccggccTAGGTACTAAAACAATTCATCCAGTA	FseI	
FP869	ACGCCATCATTCGTAAGGAC	—	Construction of SM166 and SM181
FP870	aggccggccAGCATAAAAGGCAGCCCATC	FseI	
FP871	aggcgcgccGCAATTGACCACCAGCTTTT	AscI	
FP872	CTTTGTTGGTCGCCATAGGT	—	*Kan^T^ marker in the RC strand*
FP001	aggcgcgccGTTTGATTTTTAATG	AscI	
FP002	aggccggccATCGATACAAATTCCTC	FseI	
FP869	ACGCCATCATTCGTAAGGAC	—	Construction of SM168 and SM183
FP870	aggccggccAGCATAAAAGGCAGCCCATC	FseI	
FP859	aggcgcgccTGAAATTTTCTAGCCAGTCGTTT	AscI	
FP860	TTTCAATTCTAAGAGGAGTCCAAAA	—	*Kan^T^ marker in the RC strand*
FP001	aggcgcgccGTTTGATTTTTAATG	AscI	
FP002	aggccggccATCGATACAAATTCCTC	FseI	
FP869	ACGCCATCATTCGTAAGGAC	—	Construction of SM170 and SM185
FP870	aggccggccAGCATAAAAGGCAGCCCATC	FseI	
FP863	aggcgcgccTAAAAGTTTAGACAGAGATTATAGGAAAAGG	AscI	
FP864	TGATGCTTCCATGTCAGAAGA	—	*Kan^T^ marker in the RC strand*
FP001	aggcgcgccGTTTGATTTTTAATG	AscI	
FP002	aggccggccATCGATACAAATTCCTC	FseI	
FP894	TCCGGATGCAGAAGGTATTC	—	Construction of SM180
FP895	CAATAAAAGTTCTC ACCCAATCTGGA	—	
FP896	TCCAGATTGGGTG AGAACTTTTATTG	—	
FP897	CATCCTGCCGTTCCTATCAT	—	
FP898	GGTTGATTGGGTTTTTGTGG	—	Screening primers for SM180 specific to UA159
FP899	TTTTTATGCTTTTCAATAAAAGTTCTA	—	
FP902	CTCTAAGACTAATCCAGATTGGGTT	—	
FP903	GCGAGTTTCAAAAAGGAAGC	—	
FP900	CGGATTGGATTGGGAGACTA	—	Screening primers for SM180 specific to the mutant
FP901	TTTTTATGCTTTTCAATAAAAGTTCTC	—	
FP904	CTCTAAGACTAATCCAGATTGGGTG	—	
FP905	GGCAGACAGCTTCTTTGGTC	—	
FP1552	GCGAATTGAGGAAATGGTTG	—	Construction of SM218
FP1553	TGAGTCAGCCGATCAAAGTG	—	
FP299	CCATGACCATCAACCAACAT	—	RT/PCR
FP300	ATCAGCGCGTATTACAGGTG	—	*gyrA*
FP867	AATCAGATGGCCAATTTATCG	—	RT/PCR
FP868	GCGGTTGTTTTTCGTGTGAT	—	*transcript start*
FP785	TCGTTTGCTGCAAGACTACG	—	RT/PCR
FP786	GGGAACACATTCAGCGAGTT	—	*transcript mid*
FP1199	AACGGACGGAGAAAATGTTG	—	RT/PCR
FP1200	CTTTGTTGGTCGCCATAGGT	—	*transcript end (comR)*
FP299	CCATGACCATCAACCAACAT	—	Gel electrophoresis
FP300	ATCAGCGCGTATTACAGGTG	—	*gyrA*
FP785	TCGTTTGCTGCAAGACTACG	—	Gel electrophoresis
FP1200	CTTTGTTGGTCGCCATAGGT	—	*upstream of comR*

### Genome editing

A marker-less mutation in strain SM218 was constructed using a high-efficiency strategy for genome editing^25^. Overlapping primers were designed with three single base substitutions in the SigX box of SMU.60 present upstream of *comR*. The introduction of three base substitutions inactivated the SigX box with creation of silent mutation in gene SMU.60. The resulting amplicon containing the desired substitutions were transformed in *S. mutans* UA159. Colonies were selected and screened by PCR to identify a clone containing the mutated SigX box, which was designated as SM218. The amplicon was also transformed into SM091 to place the mutation in a *comS* defective background (SM221).

### Transformation

Competence for natural transformation in *S. mutans* can be achieved either indirectly by use of CSP or directly with XIP; hence, two different protocols previously described were used for transformation in this study^21, 25^. A traditional method uses CSP in complex media such as THY and does not require chemically-defined media, while a recently described method using XIP in CDM results in higher efficiency of transformation. The traditional method was used for transformation of mutations carrying antibiotic markers^21^, since maximal transformation levels were not required. Briefly, pre-culture stocks at OD_600_ 0.5 were diluted 1:10, and CSP was added to a final concentration of 50 nM. Purified PCR products were used as donor DNA. Cultures were then incubated at 37 °C in air for 3 h. The more recent strategy using XIP in CDM was employed for construction of marker-less mutations^25^.

### Luciferase reporter assays

For luciferase assays, frozen pre-cultures of reporter strains were prepared by growth in CDM to an optical density at 600 nm (OD_600_) of 0.5, supplementation to 15% glycerol, and storage at −80 °C. For the experiments, pre-cultures were thawed, diluted 10-fold in CDM in the presence or absence of 1 μM XIP, and distributed as 200 μL aliquots in wells of 96-well microtiter plates (Thermo Fisher Scientific). Blank wells contained only CDM and luciferin. A volume of 20 μL of a 1 mM aqueous D-luciferin solution (Synchem, Felsberg-Altenberg, Germany) was added to each well, followed by sealing of the plates with Top Seal (PerkinElmer), and incubation at 37 °C. Relative luminescence units (RLU) and OD600 were measured at various time intervals during growth by reading the plates in a multi-detection microplate reader (Synergy HT and Cytation 3; BioTek).

### Real time PCR

For RNA extraction, the High Pure RNA isolation kit (Roche) was used, following the protocol provided by the producer, except that we added one step to improve cell lysis: cells were incubated at 37 °C for 20 min in 200 μL of lysis buffer containing 10 mM Tris (pH 8), 20 mg per mL of lysozyme, and 100 U of mutanolysin per mL. DNase I was used during and after RNA extraction to ensure complete removal of DNA. Templates of cDNA templates were prepared from RNA with the First Strand cDNA Synthesis Kit (Thermo Fisher Scientific). In all assays, controls without reverse transcriptase were included. Assays were carried out with quantitative PCR master mix SYBR green/ROX (Thermo Fisher Scientific), according to the manufacturer′s protocol. For data comparisons, ROX was used as a reference, and the constitutive gene *gyrA* as a normalizer. Real-time PCR was performed (Agilent Technologies Stratagene Mx3005 P), and data were collected and analyzed with the software and graphics program MxPro (Stratagene). Table 2 shows the list of primers used.

### Gel electrophoresis

DNA and cDNA amplified by True start Hot start Taq DNA Polymerase (Thermo Fisher Scientific). Gel electrophoresis was performed by preparing 1% Gel (SeaKem LE Agarose; Lonza) in TAE Buffer with SYBR safe DNA gel stain (Thermo Fisher Scientific) 1:10000. Sample and loading dye were mixed 5:1 and loaded into the wells of agarose gel. Gels were protected from light during 25–50 min at 65 V. Gene ruler 1 kb ladder was used as a size standard, and images were captured in G:Box (Syngene) with GeneSnap software v.7.12 (Syngene).

### RNA-sequencing analysis

An overnight culture of SM065 was diluted 1:10 in CDM to a final volume of 180 mL, corresponding to an optical density at 600 nm of 0.2. Total volume of diluted culture was equally divided into two tubes and grown in the presence or absence of 1 μM XIP for 2 hours at 37 °C in 5% CO_2_ atmosphere (OD_600_ of approximately 0,4); cells were then harvested for RNA extraction. Initial RNA extraction was performed with the mirVana miRNA Isolation Kit (Life Technologies), followed by DNase treatment with TURBO DNase (Life Technologies) and a second RNA extraction. RNA quality was verified by running the samples on Bioanalyzer (Agilent 2100 Expert) prior to enrichment with MICROBExpress (Thermo Fisher Scientific). Briefly, purified RNA was incubated with the Capture Oligonucleotide Mix in Binding Buffer for 15 minutes. Magnetic beads, derivatized with an oligonucleotide that hybridizes to the capture oligonucleotide, were then added to the mixture and allowed to hybridize for 15 minutes. The magnetic beads, with 16 S and 23 S rRNAs attached, were pulled to the side of the tube with a magnet. The enriched RNA in the supernatant was transferred to a new microtube. Magnetic beads were briefly washed, and finally the RNA was precipitated with ethanol. More than 90% removal of 16 S and 23 S rRNA was verified using Bioanalyzer. The RNA samples were then treated with NEXTFlex Directional RNA-seq Kit (dUTP based) for preparation of the DNA library for sequencing using Illumina® HiSeq, following the intructions provided by the producer. A FASTQ file was generated for each sample. Bioinformatic analyses were performed as described previously^34, 35^. Briefly, using the software Bowtie, nucleotide reads were mapped to the annotated genome of strain UA159 (GenBank accession: NC_004350.2). SAMtools software was used to divide the reads in each according strand. For visualization, JBrowse viewer was employed^36^. Differential expression analyses of samples with and without XIP were determined based on the mapped read profiles using a Perl script. The read counts were subjected to “DESeq” to evaluate the differential levels when comparing samples. The results are derived as means from three independently conducted biological experiments. The average and standard deviation for the total number of paired-end sequenced fragments was 7 456 931 (SD 27 663) for the control, and 7 271 703 (SD 365 331) for the XIP-treated samples.

## Electronic supplementary material

Supplementary information
