# Supplementary material for: A positive feedback loop mediated by Sigma X enhances expression of the streptococcal regulator ComR
Source: Sci Rep. 2017 Jul 20;7:5984. doi: 10.1038/s41598-017-04768-5 (PMC5519730; doi:10.1038/s41598-017-04768-5)
Supplement: Supplementary file 1 — Supplementary information [file 41598_2017_4768_MOESM1_ESM.pdf]

Supplementary information for:

A positive feedback loop mediated by Sigma X enhances expression of the streptococcal regulator ComR.

Rabia Khan, Roger Junges, Heidi A. Åmdal, Tsute Chen, Donald A. Morrison, Fernanda Petersen

Supplementary Figure S1. Selected regions in the transcriptome map showing (a) that the stress-related genes levDEFG (top) and relQ (bottom) are co-transcribed with the SigX core genes ssb2 and radC respectively, most probably as read-throughs (red arrows: induced transcripts); (b) transcripts of the SigX regulon initiated at intragenic sequences in the anti-sense (SMU.691, SMU.1853, rl16).

(a)

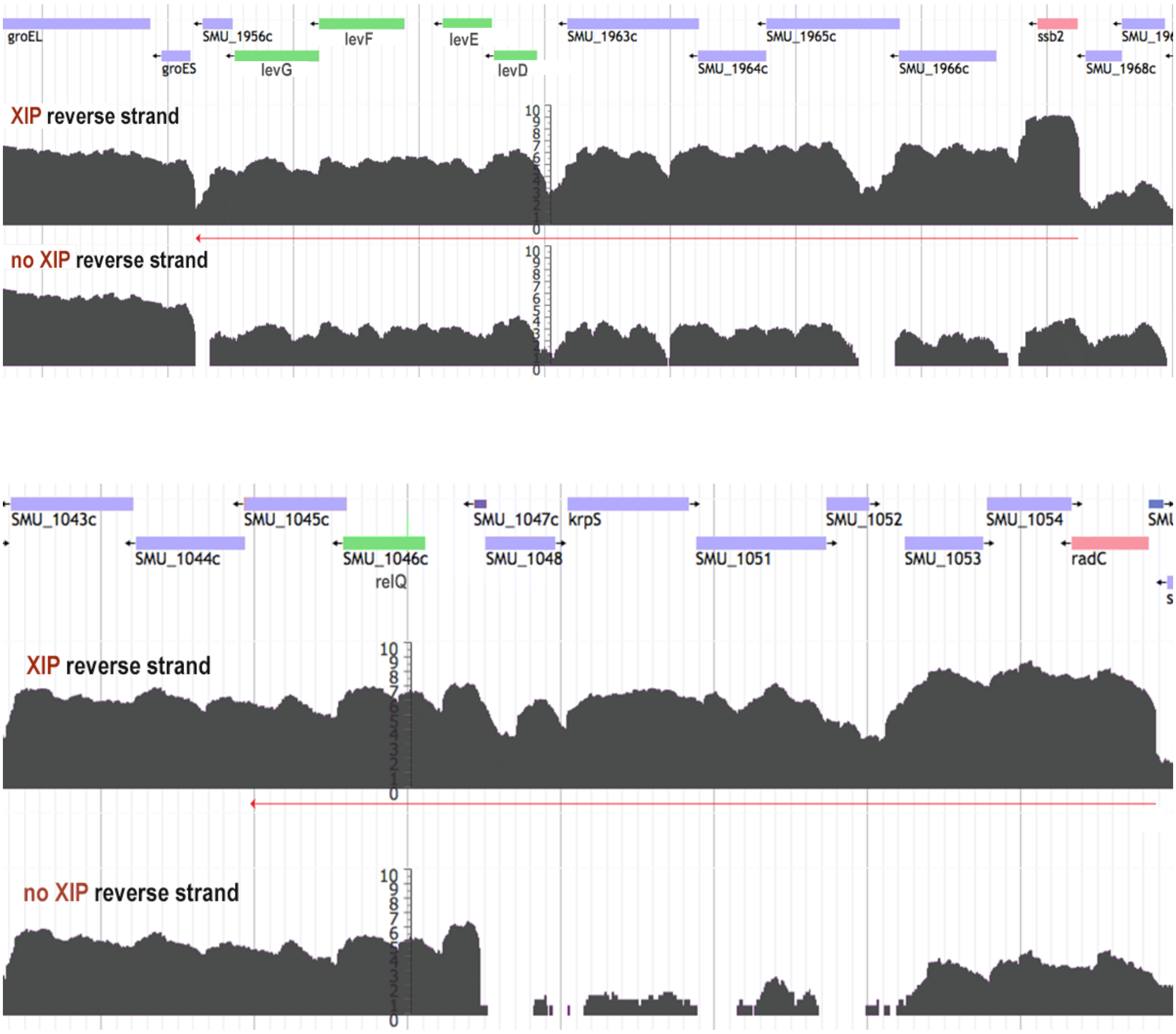

(b)

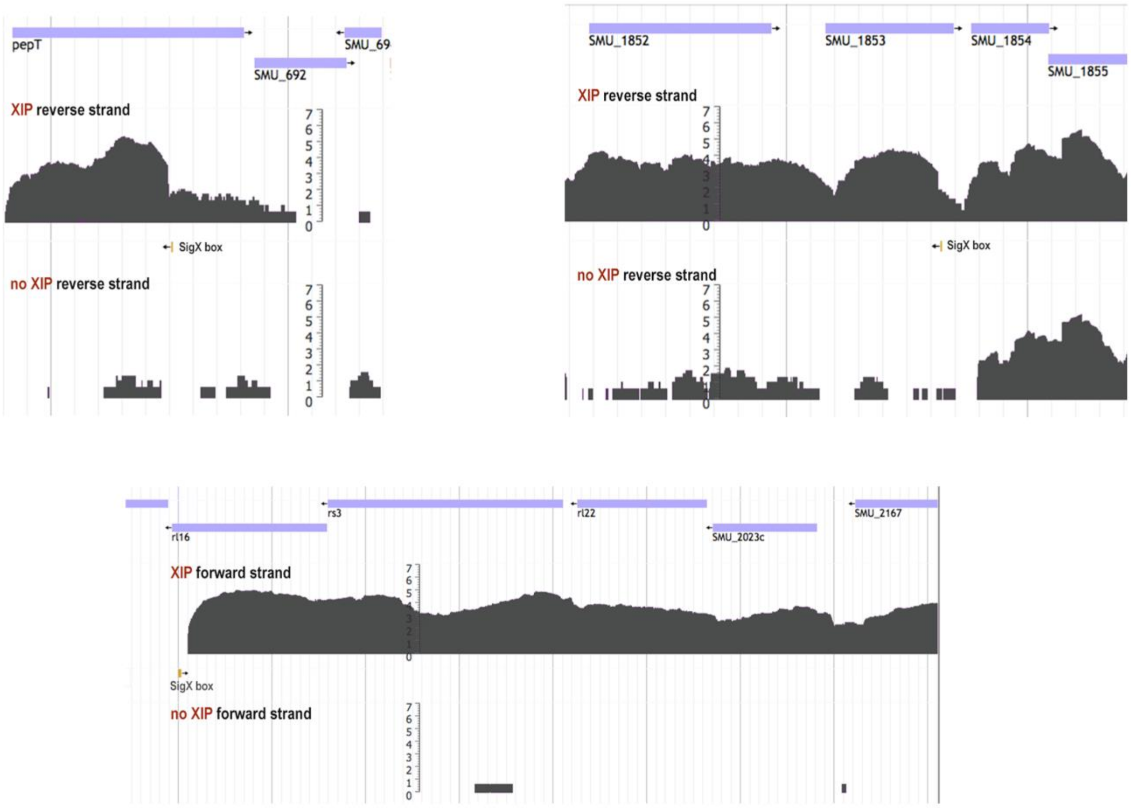
pepT
SMU_69
SMU_692
XIP reverse strand
SigX box
no XIP reverse strand
SMU_1852
SMU_1853
SMU_1854
SMU_1855
XIP reverse strand
SigX box
no XIP reverse strand
rs3
rl22
SMU_2167
rl16
SMU_2023c
XIP forward strand
SigX box
no XIP forward strand

Supplementary Table S1. Comparison of transcriptome results among three studies. Mean fold changes induced by CSP [1] or XIP [2] and this study).This study **P values < 0,05**. X; no reported changes. *Mean reads with XIP <8000. Gene locus tag as in GenBank accession no. AE014133.

Gene ID	Khan et al 2016[1]	Wenderska et al 2017[2]	This study	This study p value		Annotation
SMU.01	-1,1	x	1,0	0,79		chromosome replication initiator DnaA
SMU.02	-1,1	2,4	-1,1	0,52		DNA polymerase III subunit beta
SMU.05	1,1	x	1,6	0,14	*	hypothetical protein
SMU.06	-1	x	1,2	0,49		GTP-binding protein YchF
SMU.07	-1,1	x	1,5	**0,05**		peptidyl-tRNA hydrolase
SMU.08	-1	x	1,4	0,05		transcription-repair coupling factor
SMU.09	-1,1	x	-1,1	0,89	*	hypothetical protein
SMU.10	-1,5	x	-1,0	0,93	*	hypothetical protein
SMU.11	-1,3	x	1,5	0,43	*	hypothetical protein
SMU.12	-1,1	x	1,2	0,28		hypothetical protein
SMU.13	-1	x	1,4	0,06		cell-cycle protein
SMU.14	1,1	x	1,9	**0,00**		hypoxanthine-guanine phosphoribosyltransferase
SMU.15	1,1	x	1,6	**0,00**		cell division protein FtsH
SMU.16	-1,1	x	1,5	**0,02**		amino acid permease
SMU.18	-1,1	-4,1	-1,1	0,66		hypothetical protein
SMU.20	-1,1	x	1,1	0,48		cell shape-determining protein MreC
SMU.21	1,4	x	1,3	0,55	*	cell shape-determining protein MreD
SMU.22	1,3	x	1,9	**0,00**		secreted antigen GbpB/SagA
SMU.23	1	x	1,4	0,06		ribose-phosphate pyrophosphokinase
SMU.24	1	x	1,0	0,91		aromatic amino acid aminotransferase
SMU.25	-1,1	x	-1,2	0,72		DNA repair protein RecO
SMU.26	-1,2	x	-1,4	0,09		phosphate acyltransferase
SMU.27	-1,3	x	-1,3	0,20		acyl carrier protein
SMU.28	-1	x	1,1	0,84	*	ATP-binding protein
SMU.29	-1	-4,2	-4,1	**0,00**	*	phosphoribosylaminoimidazolesuccinocarboxamide synthase
SMU.30	-1	-4,6	-3,7	**0,01**		phosphoribosylformylglycinamidine synthase
SMU.31	-1,1	-4,3	-2,5	0,07	*	hypothetical protein
SMU.32	1	-3,7	-2,2	0,09		amidophosphoribosyltransferase
SMU.33	1,1	-3,6	-2,2	0,16	*	hypothetical protein
SMU.34	1,2	-3,3	-1,8	0,09		phosphoribosylaminoimidazole synthetase
SMU.35	1,2	-2,4	-1,5	0,16		phosphoribosylglycinamide formyltransferase
SMU.36	1,4	-2,4	-1,4	0,36		hypothetical protein
SMU.37	1,2	-2,5	-1,5	0,27		bifunctional phosphoribosylaminoimidazolecarboxamide formyltransferase/IMP cyclohydrolase
SMU.38c	1,1	x	2,2	**0,01**	*	transcriptional regulator
SMU.39	-1,3	x	-2,0	0,08	*	hypothetical protein
SMU.40	1	x	-1,5	0,43	*	hypothetical protein
SMU.41	-1,2	6	1,3	0,87	*	hypothetical protein
SMU.42	-1,1	x	1,4	0,18		hypothetical protein
SMU.43	-1,1	x	-1,4	0,44	*	site-specific DNA-methyltransferase restriction-modification protein
SMU.44	-1,5	x	-1,0	0,93	*	DNA mismatch repair protein
SMU.45	-1,2	x	-1,5	0,20	*	hypothetical protein
SMU.46	-1,2	x	1,1	0,83	*	hypothetical protein
SMU.47	-1,3	x	-1,8	0,37	*	hypothetical protein
SMU.48	-1	-2,6	-2,8	**0,00**		phosphoribosylamine--glycine ligase
SMU.49	-1	-2,3	-3,0	**0,00**		hypothetical protein
SMU.50	1	-2,7	-3,1	**0,00**		phosphoribosylaminoimidazole carboxylase catalytic subunit

SMU.51	1	-3	-3,2	**0,00**		phosphoribosylaminoimidazole carboxylase ATPase subunit
SMU.52	-1,2	-2,1	-2,9	**0,00**		hypothetical protein
SMU.53	-1,1	-2,5	-3,1	**0,00**		hypothetical protein
SMU.54	1	-2,4	-2,9	**0,00**		amino acid recemase
SMU.55	1,2	-2,1	-2,1	**0,01**	*	hypothetical protein
SMU.56	1,2	x	-1,5	0,34	*	hypothetical protein
SMU.58	-1	x	-2,3	0,20	*	hypothetical protein
SMU.59	1,2	x	-1,7	**0,01**		adenylosuccinate lyase
SMU.60	1,6	x	4,2	**0,00**		DNA alkylation repair protein
SMU.61	1,4	x	8,5	**0,00**		transcriptional regulator
SMU.63c	-2,3	8,5	-36,9	**0,00**		hypothetical protein
SMU.64	**5,6**	12	11,3	**0,00**		Holliday junction DNA helicase RuvB
SMU.65	**4,4**	11,7	9,2	**0,00**		protein tyrosine-phosphatase
SMU.66	**2,6**	6,9	4,1	**0,00**		hypothetical protein
SMU.67	**2,3**	5,8	3,8	**0,00**		acyltransferase
SMU.68	**2,3**	5,5	3,3	**0,00**		hypothetical protein
SMU.70	-1,2	x	-1,4	**0,05**		threonine synthase
SMU.71	-1	x	-1,1	0,64		cation efflux pump (multidrug resistance protein)
SMU.72	-1,2	x	-1,7	**0,01**		hypothetical protein
SMU.73	-1,1	x	-2,2	**0,00**		hypothetical protein
SMU.74	-1	x	-1,3	0,14		hypothetical protein
SMU.75	-1	x	-1,3	0,21		D-alanyl-D-alanine carboxypeptidase
SMU.76	-1	x	-1,3	0,19		N-acetyl-muramidase
SMU.78	1	x	-1,1	0,74		exo-beta-D-fructosidase
SMU.79	1,1	x	1,8	**0,03**		exo-beta-D-fructosidase
SMU.80	1,4	x	2,5	**0,00**		heat-inducible transcription repressor
SMU.81	1,5	x	1,8	**0,01**		heat shock protein GrpE
SMU.82	1,4	x	1,9	**0,04**		molecular chaperone DnaK
SMU.83	1,5	x	2,0	0,06		molecular chaperone DnaJ
SMU.84	-1	x	1,2	0,45		tRNA pseudouridine synthase A
SMU.85	-1	x	1,1	0,68		phosphomethylpyrimidine kinase
SMU.86	-1,2	x	1,0	0,94		hypothetical protein
SMU.87	-1,1	x	1,1	0,75		hypothetical protein
SMU.88c	-1,1	x	-1,4	0,10		mechanosensitive ion channel protein MscS
SMU.89c	-1,1	x	-1,2	0,48	*	nitrite transporter
SMU.91	-1,2	x	-1,2	0,27		trigger factor
SMU.92c	-1,2	x	-1,3	0,92	*	transposase fragment
SMU.93c	1,3	-2,2	1,5	0,79	*	transposase fragment
SMU.94c	1,1	x	-1,4	0,95	*	transposase fragment
SMU.96	-1	x	1,2	0,57		DNA-directed RNA polymerase subunit delta
SMU.97	1,1	x	1,6	**0,00**		CTP synthetase
SMU.99	-1	x	-1,3	0,21		fructose-bisphosphate aldolase
SMU.100	1	x	1,1	0,68		PTS system sorbose transporter subunit IIB
SMU.101	-1	x	-1,0	0,84		PTS system sorbose transporter subunit IIC
SMU.102	-1	x	-1,2	0,29		PTS system transporter subunit IID
SMU.103	1	x	-1,2	0,33		PTS system transporter subunit IIA
SMU.104	-1	x	-1,5	**0,03**		alpha-glucosidase
SMU.105	-1,1	x	-1,4	0,10		transcriptional regulator; repressor of sugar transport operon
SMU.106c	-1,2	x	-1,7	**0,02**	*	transposase fragment
SMU.107	-1,5	x	71,0	**0,00**	*	hypothetical protein
SMU.108	1,8	x	11,2	**0,00**	*	hypothetical protein
SMU.109	**2,2**	2,2	6,2	**0,00**		permease
SMU.110	1,1	x	-1,1	0,95	*	transcriptional regulator MutR
SMU.112c	1,1	x	1,4	0,17		transcriptional regulator

SMU.113	1,1	x	1,1	0,76	*	fructose-1-phosphate kinase
SMU.114	1,1	x	1,3	0,37	*	PTS system fructose-specific transporter subunit IIBC
SMU.115	1,1	x	2,4	0,10	*	PTS system fructose-specific transporter subunit IIA
SMU.116	1,2	x	1,3	0,41	*	tagatose 1,6-diphosphate aldolase
SMU.117c	1	x	1,3	0,28	*	hypothetical protein
SMU.118c	1	x	1,0	0,83		esterase
SMU.119	1,1	x	1,0	0,91		alcohol dehydrogenase
SMU.120	-1	x	-1,0	1,00		50S ribosomal protein L28
SMU.121	1,1	x	7,7	**0,00**	*	damage-inducible protein DinF
SMU.123	1,2	2,2	1,4	**0,04**		DNA polymerase III PolC
SMU.124	1,2	x	-1,2	0,46		MarR family transcriptional regulator
SMU.125	1	x	-1,1	0,56		hypothetical protein
SMU.127	-1,2	x	-1,3	0,70		acetoin dehydrogenase TPP-dependent, E1 component subunit alpha
SMU.128	-1,2	x	-1,3	0,58		acetoin dehydrogenase TPP-dependent, E1 component subunit beta
SMU.129	-1,2	x	-1,3	0,63		branched-chain alpha-keto acid dehydrogenase E2 subunit
SMU.130	-1,1	x	-1,1	0,68		dihydrolipoamide dehydrogenase
SMU.131	-1,2	x	1,0	0,87		lipoate-protein ligase
SMU.132	-1,1	2,2	1,1	0,69		hippurate amidohydrolase
SMU.133c	1	x	1,4	0,26	*	MDR permease
SMU.134	-1,1	x	-1,3	0,45	*	TetR/AcrR family transcriptional regulator
SMU.135	1	x	-1,6	**0,02**		transcriptional regulator
SMU.136c	-1,2	x	-1,3	0,39	*	transcriptional regulator
SMU.137	-1	x	1,1	0,59		malate dehydrogenase
SMU.138	-1,1	x	-1,1	0,72		malate permease
SMU.139	-1	x	1,0	0,99		hypothetical protein
SMU.140	1,1	x	1,1	0,85		glutathione reductase
SMU.141	-1	x	1,3	0,42		hypothetical protein
SMU.143c	1,1	x	-1,2	0,19		peptide deformylase
SMU.144c	1	x	1,1	0,81		transcriptional regulator
SMU.145	-1,2	x	-1,4	0,27	*	hypothetical protein
SMU.148	1,6	x	-1,5	0,13		bifunctional acetaldehyde-CoA/alcohol dehydrogenase
SMU.149	1,1	2	-1,2	0,46		transposase
SMU.150	**62,4**	2	22,0	**0,00**		hypothetical protein
SMU.151	**41,1**	2,1	26,2	**0,00**		hypothetical protein
SMU.152	**36,4**	2,3	34,0	**0,00**		hypothetical protein
SMU.153	**25,9**	x	32,7	**0,00**		hypothetical protein
SMU.154	1,5	x	1,2	0,14		30S ribosomal protein S15
SMU.155	-1,2	x	-1,5	**0,02**		polynucleotide phosphorylase
SMU.156	-1,5	x	-1,3	0,12		hypothetical protein
SMU.157	-1,1	x	-1,5	**0,03**		serine acetyltransferase
SMU.158	-1,2	x	-1,3	0,11		cysteinyl-tRNA synthetase
SMU.159	-1	x	-1,5	**0,02**		hypothetical protein
SMU.160	1,1	x	-1,1	0,77		metallopeptidase
SMU.161	1,1	x	1,1	0,80		transcriptional regulator
SMU.162c	-1,1	x	-1,1	0,52		hypothetical protein
SMU.163c	1	x	-1,1	0,60		hypothetical protein
SMU.164	-1	x	1,1	0,72		tRNA/rRNA methyltransferase
SMU.165	-1,2	x	-1,2	0,42		hypothetical protein
SMU.166	**3,7**	5,4	17,4	**0,00**		hypothetical protein
SMU.167	**2,6**	6,4	18,7	**0,00**		hypothetical protein
SMU.168	**3,5**	5,9	17,9	**0,00**		transcriptional regulator
SMU.169	1	x	1,0	0,93		50S ribosomal protein L13
SMU.170	1,1	x	-1,0	0,86		30S ribosomal protein S9
SMU.172	-1,4	x	1,4	0,07		cell growth regulatory protein

SMU.173	1	2,1	1,6	**0,03**		ppGpp-regulated growth inhibitor
SMU.174c	1	x	1,7	**0,02**		hypothetical protein
SMU.175	1	x	1,4	0,74	*	hypothetical protein
SMU.176	1,1	2,3	1,7	0,48	*	hypothetical protein
SMU.177	-1,3	x	-1,0	0,87		hypothetical protein
SMU.178	-1,2	x	-1,1	0,68		hypothetical protein
SMU.179	1,3	x	-1,0	0,75		hypothetical protein
SMU.180	1,2	x	-1,0	0,90		oxidoreductase
SMU.181	-1	x	-1,0	0,80		mevalonate kinase
SMU.182	-1,1	x	1,3	0,40		iron ABC transporter ATP-binding protein
SMU.183	-1,1	2,2	1,9	0,15		Mn/Zn ABC transporter
SMU.184	-1	x	2,2	0,18		ABC transporter metal binding lipoprotein
SMU.185	-1,3	2,1	1,2	0,74	*	hypothetical protein
SMU.186	1,1	x	1,1	0,76		metal-dependent transcriptional regulator
SMU.187c	-1,1	x	-1,5	**0,03**		hypothetical protein
SMU.188c	-1,1	x	-1,1	0,47		heat shock protein 33
SMU.189	-1	-5,6	-1,4	0,23	*	hypothetical protein
SMU.191c	-1,1	x	1,1	0,74		integrase
SMU.193c	1,2	x	1,1	0,81		hypothetical protein
SMU.194c	-1	x	1,2	0,50		hypothetical protein
SMU.195c	1,1	x	1,4	0,12		hypothetical protein
SMU.196c	1,2	x	-1,0	0,96		transfer protein
SMU.197c	1,1	x	1,0	0,94		hypothetical protein
SMU.198c	1,1	x	1,2	0,59		conjugative transposon protein
SMU.199c	1,1	x	1,3	0,36		hypothetical protein
SMU.200c	1,1	x	1,0	0,99		hypothetical protein
SMU.201c	1	x	1,1	0,82		transposon protein
SMU.202c	1	x	1,1	0,70		hypothetical protein
SMU.204c	1,1	x	1,2	0,50		hypothetical protein
SMU.205c	1	x	-1,0	0,99		hypothetical protein
SMU.206c	-1	x	-1,0	0,99		hypothetical protein
SMU.207c	1	x	1,1	0,78		transposon protein
SMU.208c	1	x	1,1	0,65		transposon protein
SMU.209c	1	x	1,4	0,23		hypothetical protein
SMU.210c	1	x	1,1	0,78		hypothetical protein
SMU.211c	1,2	x	1,2	0,75		hypothetical protein
SMU.212c	-1,1	x	1,1	0,88	*	hypothetical protein
SMU.213c	1,1	x	1,4	0,27	*	hypothetical protein
SMU.2151	-1	x	-1,0	0,81	*	phosphotidylglycerophosphate synthase
SMU.2161c	1	x	-1,8	**0,03**	*	hypothetical protein
SMU.216c	-1	2,1	1,3	0,53	*	hypothetical protein
SMU.217c	-1	2,2	1,7	**0,03**		hypothetical protein
SMU.218	-1	x	1,8	**0,04**	*	transcriptional regulator
SMU.219	-1,1	2,2	1,2	0,57	*	hypothetical protein
SMU.220c	-1,2	2,6	-1,3	0,32		hypothetical protein
SMU.221c	-1,3	x	-1,0	0,83		integrase
SMU.222c	-1,2	x	-1,9	0,12	*	integrase fragment
SMU.223c	1,2	x	-3,0	0,08	*	hypothetical protein
SMU.224c	-1,3	x	-1,8	0,18		hypothetical protein
SMU.225c	-1,6	x	-1,7	0,41	*	hypothetical protein
SMU.226c	-1,2	x	-1,9	**0,00**		transposase
SMU.227c	-1,2	x	-2,0	**0,01**		hypothetical protein
SMU.228	-1	x	1,1	0,68		alkaline-shock protein-like protein
SMU.229	1,1	x	-1,1	0,76		hypothetical protein

SMU.231	-1,2	x	-1,3	0,18		acetolactate synthase catalytic subunit
SMU.232	-1,2	x	-1,2	0,27		acetolactate synthase 3 regulatory subunit
SMU.233	-1,2	x	-1,5	**0,02**		ketol-acid reductoisomerase
SMU.234	-1	x	1,1	0,69		threonine dehydratase
SMU.235	1	x	1,0	0,85		hypothetical protein
SMU.236c	-1,2	x	-1,1	0,64		transcriptional regulator
SMU.237c	1	x	1,2	0,43	*	hypothetical protein
SMU.238c	1,1	x	1,2	0,73	*	ABC transporter ATP-binding protein
SMU.239c	-1,2	x	1,3	0,78	*	hypothetical protein
SMU.241c	-1,3	x	-1,8	**0,00**		amino acid ABC transporter ATP-binding protein
SMU.242c	-1,2	x	-1,8	**0,00**		amino acid ABC transporter permease
SMU.243	-1,2	x	-1,7	**0,00**		hypothetical protein
SMU.244	-1,2	x	-1,5	**0,02**		undecaprenyl pyrophosphate phosphatase
SMU.245	1,2	x	1,1	0,65		adaptor protein
SMU.246	1,1	x	1,1	0,70		glycosyl transferase N-acetylglucosaminyltransferase RgpG
SMU.247	-1	x	-1,1	0,54		ABC transporter ATP-binding protein
SMU.248	-1	x	-1,2	0,34		ABC transporter membrane protein
SMU.249	-1,1	x	-1,1	0,49		NifS protein-like protein class-V aminotransferase
SMU.250	-1,1	x	-1,2	0,37		nitrogen fixation-like protein, NifU
SMU.251	-1,1	x	-1,1	0,48		ABC transporter membrane protein
SMU.252	-1,1	x	-1,5	**0,04**		hypothetical protein
SMU.253	-1,1	x	-1,0	0,90		D-alanyl-D-alanine carboxypeptidase
SMU.255	-1,1	x	-1,3	0,10		oligopeptide ABC transporter substrate-binding protein OppA
SMU.256	-1,1	x	-1,2	0,20		oligopeptide transport system permease OppB
SMU.257	-1,2	x	-1,3	0,15		transmembrane permease OppC
SMU.258	-1,1	x	-1,4	**0,04**		oligopeptide ABC transporter ATP-binding protein OppD
SMU.259	-1	x	-1,4	**0,05**		oligopeptide ABC transporter ATP-binding protein OppF
SMU.260	-1	x	-1,1	0,72		hypothetical protein
SMU.261c	-1	3,4	1,6	0,12	*	transcriptional regulator
SMU.262	1	4,9	3,8	**0,04**	*	putrescine carbamoyltransferase
SMU.263	1,1	x	3,1	0,07	*	amino acid antiporter
SMU.264	1,2	2	5,6	**0,00**	*	agmatine deiminase
SMU.265	1,3	x	6,9	**0,00**	*	amino acid kinase
SMU.267c	-1	x	1,1	0,66		bifunctional glutamate--cysteine ligase/glutathione synthetase
SMU.268	-1,1	x	-1,4	0,07		adenylosuccinate synthetase
SMU.270	-1,2	x	-1,3	0,36		PTS system ascorbate-specific transporter subunit IIC
SMU.271	-1,2	x	-1,5	0,07	*	PTS system transporter subunit IIB
SMU.272	-1,2	x	-1,3	0,15		PTS system transporter subunit IIA
SMU.273	-1,2	x	-1,6	**0,01**		3-keto-L-gulonate-6-phosphate decarboxylase
SMU.274	-1,2	x	-1,5	**0,04**		L-xylulose 5-phosphate 3-epimerase
SMU.275	-1,2	x	-1,5	**0,05**		L-ribulose-5-phosphate 4-epimerase
SMU.276c	1,1	x	77,6	0,32	*	hypothetical protein
SMU.277	-1,1	-2	-2,9	**0,00**		hypothetical protein
SMU.278	-1,3	-2,7	-2,3	**0,00**		hypothetical protein
SMU.279	-1,2	-2,5	-1,7	0,33	*	hypothetical protein
SMU.281	-1,1	-2,6	-1,6	**0,03**		hypothetical protein
SMU.283	-1	x	-2,0	**0,00**	*	hypothetical protein
SMU.284	-1,4	-2,3	-2,3	**0,00**	*	hypothetical protein
SMU.285	-2,1	x	-2,2	0,10	*	hypothetical protein
SMU.286	-1,2	x	-1,5	**0,02**		ABC transporter ATP-binding protein ComA
SMU.287	-1,4	x	-1,3	0,15		ComB, accessory factor for ComA
SMU.289	-1,2	x	-1,0	0,85		transcriptional regulator
SMU.290	-1	2,2	1,0	0,99		L-ascorbate 6-phosphate lactonase
SMU.291	-1,1	x	-1,7	**0,00**		transketolase

SMU.292	1,1	x	1,2	0,28		transcriptional regulator
SMU.293	-1	x	-1,2	0,42	*	hypothetical protein
SMU.294	-1	x	1,2	0,36		hypothetical protein
SMU.295	1	x	1,2	0,49		hypothetical protein
SMU.296	1,1	x	1,0	1,00		hypothetical protein
SMU.297	-1	x	-1,1	0,46		DNA polymerase I
SMU.298	-1	x	-1,1	0,67		hypothetical protein
SMU.299c	-1,1	3,4	-1,6	0,36		bacteriocin peptide
SMU.300	1,1	x	1,0	0,89		queuine tRNA-ribosyltransferase
SMU.301	1,1	x	1,0	0,95		hypothetical protein
SMU.302	1	x	-1,1	0,76		hypothetical protein
SMU.303	1,1	x	1,0	0,89		hypothetical protein
SMU.304	1	x	1,0	0,89		deaminase
SMU.305	-1	x	1,1	0,50		hypothetical protein
SMU.307	1,2	x	-1,0	0,86		glucose-6-phosphate isomerase
SMU.308	1,1	x	1,1	0,58		sorbitol-6-phosphate 2-dehydrogenase
SMU.309	-1,1	x	1,1	0,56		regulator of sorbitol operon
SMU.310	1	x	1,3	0,40	*	sorbitol operon activator
SMU.311	1,2	x	1,4	0,31	*	PTS system sorbitol (glucitol) transporter subunit IIC2
SMU.312	1,1	x	1,2	0,52		PTS system sorbitol phosphotransferase transporter subunit IIBC
SMU.313	1,2	x	1,1	0,83	*	PTS system sorbitol-specific transporter subunit IIA
SMU.314	1	x	-1,5	0,41	*	hypothetical protein
SMU.317	1	x	-1,2	0,24		tetrahydrodipicolinate succinylase
SMU.318	-1	x	-1,5	**0,02**		hippurate hydrolase
SMU.320	1	x	1,1	0,62		5-formyltetrahydrofolate cyclo-ligase
SMU.321	-1	x	1,0	0,83		hypothetical protein
SMU.322c	-1	x	1,2	0,22		glucose-1-phosphate uridylyltransferase
SMU.323	1,1	x	1,1	0,73		NAD(P)H-dependent glycerol-3-phosphate dehydrogenase
SMU.325	**3**	x	5,6	**0,00**		deoxyuridine 5'-triphosphate nucleotidohydrolase
SMU.326	**3,3**	x	5,6	**0,00**		hypothetical protein
SMU.327	**3,1**	x	5,6	**0,00**		DNA repair protein RadA
SMU.328	1,9	x	3,6	**0,00**		carbonic anhydrase
SMU.329	1,7	x	3,7	**0,00**		hypothetical protein
SMU.330	-1	x	-1,0	0,83		glutamyl-tRNA synthetase
SMU.331	1	x	-1,0	0,93		transcriptional regulator
SMU.332	1	x	1,1	0,70		hypothetical protein
SMU.333	-1,3	x	-1,4	0,06		hypothetical protein
SMU.334	-1,3	x	1,0	0,93		argininosuccinate synthase
SMU.335	-1,3	x	1,1	0,76		argininosuccinate lyase
SMU.336	-1,1	x	1,1	0,50		ribonuclease P
SMU.337	-1,1	x	-1,1	0,64		hypothetical protein
SMU.338	-1,2	x	-1,1	0,68		RNA-binding protein
SMU.339	-1,4	x	-1,3	0,33		hypothetical protein
SMU.340	1,1	2,6	1,1	0,57		50S ribosomal protein L34
SMU.341	1,1	x	-1,1	0,73		deoxyribonuclease
SMU.342	-1	x	-1,1	0,47		hypothetical protein
SMU.343	1,1	x	1,0	0,93		hypothetical protein
SMU.344	1,3	x	1,1	0,64		hypothetical protein
SMU.345c	1	2	1,2	0,71	*	hypothetical protein
SMU.346	1,2	2,4	1,4	0,08		NADH dehydrogenase; NAD(P)H nitroreductase
SMU.348	-1,1	x	1,1	0,60		histidine triad (HIT) hydrolase
SMU.349	1,1	x	1,2	0,24		KsgA/Dim1 family 16S ribosomal RNA methyltransferase
SMU.350	1,1	x	1,2	0,35		hypothetical protein
SMU.351	1,3	x	3,7	**0,00**		GTPase RsgA

SMU.352	**6**	4	16,7	**0,00**		ribulose-phosphate 3-epimerase
SMU.353	**5,6**	4,2	15,4	**0,00**		hypothetical protein
SMU.354	**5,4**	4,5	15,2	**0,00**		hypothetical protein
SMU.355	**5,9**	4,9	16,5	**0,00**		CMP-binding factor
SMU.356	**3,2**	4	17,4	**0,00**		pur operon repressor
SMU.357	1,1	x	1,4	0,07		30S ribosomal protein S12
SMU.358	1,2	x	1,4	0,06		30S ribosomal protein S7
SMU.359	1,1	x	1,3	0,09		elongation factor G
SMU.360	1,1	x	-1,3	0,17		extracellular glyceraldehyde-3-phosphate dehydrogenase
SMU.361	-1	x	-1,4	0,09		phosphoglycerate kinase
SMU.362	-1,1	x	1,4	0,15		hypothetical protein
SMU.363	-1,1	-2,8	-1,9	0,36		transcriptional regulator; glutamine synthetase repressor
SMU.364	-1,2	-2,7	-2,0	0,28		glutamate--ammonia ligase
SMU.365	-1,3	x	-1,8	0,27		glutamate synthase
SMU.366	-1,3	x	-2,0	0,21		glutamate synthase
SMU.367	-1,1	x	-1,6	0,34		hypothetical protein
SMU.368c	1,1	x	1,2	0,40		hypothetical protein
SMU.369c	-1,2	x	1,4	0,12		hypothetical protein
SMU.370	1	x	1,5	0,16	*	ABC transporter ATP-binding protein
SMU.371	-1	x	-1,2	0,72	*	hypothetical protein
SMU.372	-1	x	-1,1	0,93	*	hypothetical protein
SMU.373	1,1	x	1,5	0,17	*	hypothetical protein
SMU.374	-1	x	1,2	0,45	*	oxidoreductase
SMU.375	1,1	x	2,3	**0,01**	*	hypothetical protein
SMU.376	1	x	1,7	**0,02**		aminotransferase
SMU.378	-1,2	x	1,3	0,20		hypothetical protein
SMU.379	1,3	2,7	1,3	0,25	*	hypothetical protein
SMU.381c	1	x	-1,1	0,74		hypothetical protein
SMU.382c	-1	x	1,1	0,68	*	oxidoreductase
SMU.383c	-1	x	1,5	0,27	*	reductase
SMU.384	-1,1	x	1,2	0,39		hypothetical protein
SMU.385	1,1	x	-1,0	0,87		glycoprotein endopeptidase
SMU.386	1	x	1,3	0,17		ribosomal-protein-alanine acetyltransferase
SMU.387	1	x	1,2	0,43		UGMP family protein
SMU.388	-1,1	x	-1,3	0,19		branched-chain amino acid ABC transporter permease
SMU.389	1,1	x	-1,1	0,65		hypothetical protein
SMU.390	-1,1	x	-1,4	0,17	*	hypothetical protein
SMU.391c	1	x	-1,2	0,21		hypothetical protein
SMU.392c	-1	x	1,1	0,58		hypothetical protein
SMU.393	-1,1	x	1,2	0,55		hypothetical protein
SMU.394c	1	x	-1,1	0,58		hypothetical protein
SMU.395	-1,1	x	1,2	0,42		x-prolyl-dipeptidyl aminopeptidase
SMU.396	-1,2	x	-1,3	0,27		glycerol uptake facilitator protein
SMU.399	1,6	x	5,5	**0,00**		hypothetical protein
SMU.400	**2**	x	8,2	**0,00**		secreted esterase
SMU.401c	-1,2	x	-1,1	0,79	*	hypothetical protein
SMU.402	1,1	x	-1,4	0,06		pyruvate formate-lyase
SMU.403	1,1	x	1,5	**0,02**		DNA polymerase IV
SMU.404c	1,2	x	1,8	0,11	*	hypothetical protein
SMU.405c	-1,2	x	1,3	0,46	*	transcriptional regulator
SMU.406c	-1,1	x	-1,4	0,08		hypothetical protein
SMU.407	1	x	1,3	0,33	*	hypothetical protein
SMU.408	-1,1	x	-1,6	**0,01**		permease
SMU.409	1,2	x	1,0	0,98		hypothetical protein

SMU.410	-1	x	-1,2	0,33		transcriptional regulator
SMU.411c	1,1	x	-1,1	0,77		hypothetical protein
SMU.412c	-1	x	1,2	0,48		cell-cycle regulation protein
SMU.413	1	x	-1,0	0,89		ABC transporter ATP-binding protein
SMU.414	-1,1	x	-1,1	0,66		ABC transporter permease
SMU.415	-1,1	x	1,0	0,88		hypothetical protein
SMU.416	-1,1	x	-1,1	0,68		tRNA (guanine-N(7)-)-methyltransferase
SMU.417	-1,1	x	1,3	0,08		ribosome maturation protein RimP
SMU.418	1,1	x	1,3	0,10		transcription elongation factor NusA
SMU.419	-1,3	x	1,4	**0,04**		hypothetical protein
SMU.420	-1,1	x	1,6	**0,01**		hypothetical protein
SMU.421	-1	x	1,4	**0,03**		translation initiation factor IF-2
SMU.422	-1,2	x	1,7	**0,00**		ribosome-binding factor A
SMU.423	**49,5**	4	31,4	**0,00**		hypothetical protein
SMU.424	**5,9**	x	3,8	**0,00**		negative transcriptional regulator, CopY
SMU.426	**5,5**	x	2,9	**0,00**		copper-transporting ATPase
SMU.427	**4**	x	2,4	**0,00**		copper chaperone
SMU.428	1,1	x	1,0	0,92		hypothetical protein
SMU.429c	-1,3	x	1,0	0,92	*	hypothetical protein
SMU.431	-1	x	1,3	0,62		ABC transporter ATP-binding protein
SMU.432	1	x	1,1	0,93		ABC transporter
SMU.433	-1,2	x	-1,6	**0,02**		transcriptional regulator
SMU.434	-1,3	x	-1,5	**0,04**		hypothetical protein
SMU.435	1	x	1,4	**0,04**		N-acetylglucosamine-6-phosphate deacetylase
SMU.436c	-1,1	x	1,4	0,38	*	transposase, ISSmu1
SMU.438c	-1	x	-1,2	0,34		(R)-2-hydroxyglutaryl-CoA dehydratase activator-like protein
SMU.439	1,2	x	3,4	**0,00**	*	transcriptional regulator
SMU.440	1,2	x	1,4	0,22		hypothetical protein
SMU.441	1,1	x	1,2	0,35		transcriptional regulator
SMU.442	1,1	x	-1,1	0,69		hypothetical protein
SMU.444	-1,3	x	-1,6	0,31	*	hypothetical protein
SMU.445	-1,2	x	-1,5	0,09		glycyl-tRNA synthetase subunit alpha
SMU.446	-1,3	x	-1,5	**0,01**		glycyl-tRNA synthetase subunit beta
SMU.447	-1,2	x	-1,5	0,06		hypothetical protein
SMU.448	-1,1	x	-1,6	**0,01**		hypothetical protein
SMU.449	-1,3	x	-1,4	**0,05**		gamma-glutamyl kinase
SMU.450	-1,1	x	-1,5	**0,02**		gamma-glutamyl phosphate reductase
SMU.451	-1,1	x	-1,6	0,24	*	hypothetical protein
SMU.453	-1	x	-1,1	0,53		16S rRNA m(4)C1402 methyltransferase
SMU.454	-1,1	x	-1,0	0,97		cell division protein
SMU.455	-1	x	-1,1	0,63		penicillin-binding protein 2X
SMU.456	-1,1	x	-1,3	0,10		phospho-N-acetylmuramoyl-pentapeptide-transferase
SMU.457	-1,3	x	-1,4	0,61	*	hypothetical protein
SMU.458	-1,1	x	-1,0	0,86		ATP-dependent RNA helicase
SMU.459	1	x	1,6	0,06		ABC transporter amino acid binding protein
SMU.460	1	x	1,1	0,58		amino acid ABC transporter permease
SMU.461	-1,1	x	1,1	0,77		amino acid ABC transporter ATP-binding protein
SMU.462	-1,5	x	1,1	0,74	*	hypothetical protein
SMU.463	-1,1	x	1,1	0,79		thioredoxin reductase
SMU.464	-1,1	x	-1,3	0,11		nicotinate phosphoribosyltransferase
SMU.465	-1,2	x	-1,3	0,10		NAD synthetase
SMU.466	-1,1	x	-1,4	**0,05**		cysteine aminopeptidase
SMU.467	1	x	1,0	0,97		penicillin-binding protein 1a; membrane carboxypeptidase
SMU.469	1	x	-1,1	0,52		Holliday junction-specific endonuclease

SMU.470	1,4	x	3,5	**0,00**		hypothetical protein
SMU.471	-1,2	x	-1,1	0,74		hypothetical protein
SMU.472	-1,1	x	1,3	0,17		N-6 adenine-specific DNA methylase
SMU.473	-1	2,3	1,2	0,35		hypothetical protein
SMU.474	-1,2	x	-1,5	**0,03**		S-ribosylhomocysteinase
SMU.475	1,1	x	1,0	0,95		hypothetical protein
SMU.478	1	x	-1,3	0,16		guanylate kinase
SMU.479	-1,2	x	-1,2	0,22		DNA-directed RNA polymerase subunit omega
SMU.480	-1,2	x	-1,2	0,18		primosome assembly protein PriA
SMU.481	-1,1	x	-1,2	0,21		methionyl-tRNA formyltransferase
SMU.482	-1,1	x	-1,2	0,26		16S rRNA methyltransferase GidB
SMU.483	1,1	x	-1,1	0,57		phosphoprotein phosphatase
SMU.484	1	x	-1,1	0,53		serine/threonine protein kinase
SMU.485	1,1	x	1,1	0,62		hypothetical protein
SMU.486	1,1	x	1,0	0,97		histidine kinase
SMU.487	-1,1	x	1,1	0,54		response regulator
SMU.488	-1,1	x	-1,1	0,70		hydrolase
SMU.489	-1,2	x	-1,1	0,77		hypothetical protein
SMU.490	1,2	x	3,5	**0,00**	*	pyruvate formate-lyase activating enzyme
SMU.491	-1,1	x	1,2	0,51	*	DeoR-type transcriptional regulator
SMU.493	1,2	x	1,7	**0,01**		formate acetyltransferase
SMU.494	1,2	x	1,5	0,09		fructose-6-phosphate aldolase
SMU.495	1,1	x	1,4	0,15		glycerol dehydrogenase
SMU.496	1	x	1,1	0,61		cysteine synthetase A
SMU.497c	-1,1	x	1,1	0,81	*	hypothetical protein
SMU.498	**64,8**	351	527,1	**0,00**		late competence protein
SMU.499	**40,5**	277,3	751,2	**0,00**		late competence protein
SMU.500	**3,2**	2,1	12,5	**0,00**		ribosome-associated protein
SMU.501	-1,5	-2,3	-1,6	**0,01**		hypothetical protein
SMU.502	-1,4	-2,3	-1,5	**0,03**		hypothetical protein
SMU.503c	-1,3	-2,4	-3,3	**0,00**	*	hypothetical protein
SMU.504	-1,3	x	-1,3	0,45	*	site-specific DNA-methyltransferase
SMU.505	**14,4**	9,4	65,9	**0,00**		adenine-specific DNA methylase
SMU.506	**5,6**	7,4	45,9	**0,00**		type II restriction endonuclease
SMU.507	**4,9**	4,9	28,1	**0,00**		DeoR family transcriptional regulator
SMU.508	**4,9**	4,5	26,3	**0,00**		hypothetical protein
SMU.509	1	x	3,4	**0,00**		hypothetical protein
SMU.510c	-1,5	-2,5	-3,3	**0,00**		hypothetical protein
SMU.512c	-1	x	-1,8	0,07	*	hypothetical protein
SMU.513	-1,3	5,1	1,7	**0,04**		hypothetical protein
SMU.514	-1,2	6,6	1,7	**0,04**		transcriptional regulator
SMU.515	-1	7,3	1,7	**0,02**		hypothetical protein
SMU.516	-1,1	x	-1,2	0,32		hypothetical protein
SMU.517	-1,2	x	-1,6	**0,01**		phosphopantetheine adenylyltransferase
SMU.518	-1,1	x	-1,5	**0,03**		hypothetical protein
SMU.520	-1	x	-1,2	0,39		hypothetical protein
SMU.521	-1,2	x	-1,0	0,98		hypothetical protein
SMU.522	-1,1	x	-1,1	0,74		ribosomal RNA large subunit methyltransferase N
SMU.523	-1,3	2,1	-1,1	0,77		hypothetical protein
SMU.524	-1,1	x	1,3	0,24		ABC transporter ATP-binding protein
SMU.525	-1,1	x	1,3	0,18		ABC transporter ATP-binding protein
SMU.526c	-1	x	-1,1	0,96	*	transcriptional regulator
SMU.527	-1,1	x	-1,0	0,91		hypothetical protein
SMU.528c	-1	x	-1,2	0,59	*	hypothetical protein

SMU.529	1,1	x	1,5	0,55	*	hypothetical protein
SMU.530c	1,3	x	4,2	**0,00**		hypothetical protein
SMU.531	-1,4	x	-8,7	**0,00**	*	chorismate mutase
SMU.532	-1,4	2,6	-13,6	**0,00**	*	anthranilate synthase component I
SMU.533	-1,4	4,4	-12,6	**0,00**	*	anthranilate synthase component II
SMU.534	-1,5	2,7	-15,8	**0,00**	*	anthranilate phosphoribosyltransferase
SMU.535	-1,5	4,1	-27,9	**0,00**	*	indole-3-glycerol phosphate synthase
SMU.536	-1,7	6,1	-33,4	**0,00**	*	N-(5'-phosphoribosyl)anthranilate isomerase
SMU.537	-1,4	6,2	-9,6	**0,00**	*	tryptophan synthase subunit beta
SMU.538	-1,7	15,8	-6,5	**0,00**	*	tryptophan synthase subunit alpha
SMU.539c	**8,7**	45,1	358,7	**0,00**		signal peptidase type IV
SMU.540	1	x	-1,8	**0,04**		peroxide resistance protein Dpr
SMU.541	1,1	x	-1,5	0,05		hypothetical protein
SMU.542	1	x	-1,8	**0,00**		glucose kinase
SMU.543	-1,3	x	-2,0	**0,00**		hypothetical protein
SMU.545	-1,4	x	-1,5	0,35	*	hypothetical protein
SMU.546	-1,1	x	-1,6	**0,00**		GTP-binding protein
SMU.547	-1,3	x	-1,7	**0,00**		hypothetical protein
SMU.548	-1,1	x	1,0	0,85		UDP-N-acetylmuramoyl-L-alanyl-D-glutamate synthetase
SMU.549	-1,2	x	-1,1	0,76		undecaprenyldiphospho-muramoylpentapeptide beta-N-acetylglucosaminyltransferase
SMU.550	-1,3	x	-1,1	0,71		cell division protein FtsQ
SMU.551	-1	x	-1,2	0,20		cell division protein FtsA
SMU.552	-1	x	-1,2	0,18		cell division protein FtsZ
SMU.553	-1,1	x	-1,3	0,16		hypothetical protein
SMU.554	-1,1	x	-1,3	0,13		hypothetical protein
SMU.555	-1,1	x	-1,8	0,15		hypothetical protein
SMU.556	-1,3	x	-1,1	0,56		hypothetical protein
SMU.557	-1,1	x	-1,1	0,71		cell division protein DivIVA
SMU.558	-1,3	x	-1,4	**0,03**		isoleucyl-tRNA synthetase
SMU.560c	-1,2	-2	1,2	0,40		hypothetical protein
SMU.561c	-1	x	1,7	**0,00**		hydrolase (MutT family)
SMU.562	-1,1	x	1,1	0,68		ATP-dependent protease ClpE
SMU.563	-1,2	x	1,5	0,18		ornithine carbamoyltransferase
SMU.564	1	x	-1,1	0,79		hypothetical protein
SMU.565c	-1,2	x	1,5	0,39	*	transposase, ISSmu1
SMU.566c	-1,1	x	3,7	0,13	*	hypothetical protein
SMU.567	-1,2	x	-1,2	0,32		glutamine ABC transporter permease
SMU.568	-1,2	x	-1,2	0,20		amino acid ABC transporter ATP-binding protein
SMU.569	1,2	x	1,1	0,63		ferrous ion transport protein A
SMU.570	1,2	x	1,0	0,82		ferrous ion transport protein B
SMU.571	1,3	x	-1,0	0,96		hypothetical protein
SMU.572	1,1	x	-1,2	0,31		bifunctional 5,10-methylene-tetrahydrofolate dehydrogenase/ 5,10-methylene-tetrahydrofolate cyclohydrolase
SMU.573	-1,1	x	-1,3	0,17		hypothetical protein
SMU.574c	-1	2,3	1,1	0,88	*	hypothetical protein
SMU.575c	-1	x	-1,8	0,38	*	hypothetical protein
SMU.576	1,1	x	-1,6	**0,01**		response regulator LytR
SMU.577	-1	x	-1,5	**0,02**		histidine kinase LytS
SMU.580	1,1	x	1,1	0,47		exodeoxyribonuclease VII large subunit
SMU.581	-1,1	x	1,2	0,42		exodeoxyribonuclease VII small subunit
SMU.582	-1,1	x	1,2	0,31		farnesyl diphosphate synthase
SMU.583	-1,1	x	1,2	0,30		hemolysin
SMU.584	-1	x	1,2	0,34		arginine repressor ArgR
SMU.585	1,3	x	1,6	**0,01**		DNA repair protein RecN
SMU.586	1	x	1,3	0,15		hypothetical protein

SMU.587	-1,1	x	1,3	0,08		hypothetical protein
SMU.588	-1	x	1,5	**0,02**		hypothetical protein
SMU.589	1	x	-1,3	0,16		DNA-binding protein
SMU.590c	-1,2	4	1,7	0,36	*	transposase, fragment
SMU.591c	-1,2	3,2	1,4	0,37	*	hypothetical protein
SMU.592c	1,4	3,9	-1,1	0,91	*	transcriptional regulator
SMU.593	1	x	1,6	**0,01**		ferric uptake regulator protein FurR
SMU.594	1,5	-2	1,6	0,39	*	hypothetical protein
SMU.595	-1,2	x	-1,6	0,09		dihydroorotate dehydrogenase 1A
SMU.596	-1,2	x	-1,5	**0,02**		phosphoglyceromutase
SMU.597	-1	x	-1,1	0,80		penicillin-binding protein 2b
SMU.598	1	x	1,1	0,63		recombination protein RecR
SMU.599	-1,1	x	-1,2	0,24		D-alanyl-alanine synthetase A
SMU.600c	1,1	x	-1,1	0,55		hypothetical protein
SMU.602	-1	x	1,1	0,76		sodium-dependent transporter
SMU.603	-1,1	x	-1,2	0,38		UDP-N-acetylmuramoylalanyl-D-glutamyl-2,6- diaminopimelate-D-alanyl-D-alanyl ligase pentapeptide synthase
SMU.604	-1,2	x	-1,7	0,49	*	hypothetical protein
SMU.605	-1,2	x	-1,3	0,56	*	hypothetical protein
SMU.606	-1,2	x	1,0	0,89		hypothetical protein
SMU.607	-1,1	x	-1,2	0,49		hypothetical protein
SMU.608	-1,1	x	-1,5	**0,02**		peptide chain release factor 3
SMU.609	-1,2	x	-1,6	0,45		40K cell wall protein
SMU.610	-1,2	-6	-1,7	**0,00**		cell surface antigen SpaP
SMU.611	1,3	x	1,7	**0,00**		ATP-dependent RNA helicase, DEAD-box family
SMU.613	1,3	x	1,8	**0,00**		hypothetical protein
SMU.614	1	x	1,6	0,10		hypothetical protein
SMU.616	1,3	x	-1,4	0,30		hypothetical protein
SMU.618	-1,2	x	-1,3	0,31		hypothetical protein
SMU.620	1,1	x	1,1	0,60	*	hypothetical protein
SMU.621c	-1	x	-1,1	0,55		hypothetical protein
SMU.622c	-1,2	x	-1,5	0,10	*	hypothetical protein
SMU.623c	-1	x	-1,3	0,17		deacetylase
SMU.624	-1,1	x	-1,2	0,33		1-acylglycerol-3-phosphate O-acyltransferase
SMU.625	**131,7**	183,8	280,0	**0,00**		competence protein
SMU.626	**61,7**	394,6	305,7	**0,00**		competence protein
SMU.627	**3,3**	2,2	7,2	**0,00**		hypothetical protein
SMU.628	1,3	x	5,3	**0,00**		DNA polymerase III subunit delta
SMU.629	-1,4	x	-1,1	0,78		manganese-type superoxide dismutase, Fe/Mn-SOD
SMU.630	-1,2	3,1	-1,1	0,57		hypothetical protein
SMU.631	-1,2	3,2	1,2	0,40		hypothetical protein
SMU.632	1	x	1,1	0,55		transcriptional regulator
SMU.633	-1,2	x	1,0	0,96		thioesterase
SMU.634	-1	x	1,2	0,32		S-adenosylmethionine--tRNA ribosyltransferase-isomerase
SMU.635	1,5	x	1,4	**0,04**		hypothetical protein
SMU.636	1,3	x	2,3	**0,00**		N-acetylglucosamine-6-phosphate isomerase
SMU.637c	1,1	x	-1,1	0,81	*	hypothetical protein
SMU.638	1,1	x	2,4	**0,00**		16S pseudouridylate synthase
SMU.639	1,1	x	2,2	**0,00**		acetyltransferase
SMU.640c	-1	x	1,2	0,64	*	GntR family transcriptional regulator
SMU.641	1,2	2,3	2,2	**0,00**		oxidoreductase
SMU.642	-1,1	2,2	1,4	0,17	*	hypothetical protein
SMU.643	-1	3,3	2,6	**0,00**		esterase
SMU.644	**39,7**	216	202,7	**0,00**		competence protein/transcription factor
SMU.645	**9,8**	15,6	61,6	**0,00**		oligopeptidase

SMU.646	**10,3**	13,6	48,0	**0,00**		phosphatase
SMU.647	1,8	3,4	9,5	**0,00**		methyltransferase
SMU.648	1	x	2,4	**0,00**		foldase PrsA
SMU.649	-1,2	x	1,2	0,23		hypothetical protein
SMU.650	-1	x	1,1	0,61		alanyl-tRNA synthetase
SMU.651c	1,4	x	6,0	**0,00**	*	ABC transporter substrate-binding protein
SMU.652c	1,3	x	7,3	**0,00**	*	nitrate ABC transporter ATP-binding protein
SMU.653c	1,3	2,5	5,0	**0,00**	*	ABC transporter permease
SMU.654	-1	2	6,5	**0,00**	*	ABC transporter ATP-binding protein MutF
SMU.655	-1	x	3,0	0,33	*	protein MutE
SMU.656	1	3,3	2,1	0,42	*	protein MutE
SMU.657	1	2,3	-1,2	0,92	*	MutG
SMU.658	1	x	2,9	**0,03**	*	hypothetical protein
SMU.659	1,1	x	-1,2	0,42	*	response regulator SpaR
SMU.660	-1	x	1,2	0,34		histidine kinase SpaK
SMU.661	-1,1	x	-1,0	0,97	*	transcriptional regulator
SMU.662	-1,1	x	-1,3	0,39	*	hypothetical protein
SMU.663	1	3,2	2,2	**0,03**	*	N-acetyl-gamma-glutamyl-phosphate reductase
SMU.664	1,1	3	2,2	**0,04**	*	bifunctional ornithine acetyltransferase/N-acetylglutamate synthase
SMU.665	1,2	2,5	2,6	**0,02**	*	acetylglutamate kinase
SMU.666	1,1	2,4	4,4	**0,00**		acetylornithine aminotransferase
SMU.667	-1,1	x	-1,6	**0,01**		ribonucleotide-diphosphate reductase subunit beta
SMU.668c	-1,3	x	-1,8	**0,00**		ribonucleotide-diphosphate reductase subunit alpha
SMU.669c	-1,3	x	-2,0	**0,00**		glutaredoxin
SMU.670	-1,2	-4	-1,5	0,50		aconitate hydratase
SMU.671	-1,1	-4,2	-1,6	0,45		citrate synthase
SMU.672	-1,1	-3,9	-1,7	0,45		isocitrate dehydrogenase
SMU.673	-1	-2,4	-1,3	0,64		hypothetical protein
SMU.674	1,2	-2,8	-1,2	0,39		phosphocarrier protein HPr
SMU.675	1,1	x	-1,1	0,52		PTS system transporter protein EI
SMU.676	1	x	-1,1	0,58		NADP-dependent glyceraldehyde-3-phosphate dehydrogenase
SMU.677	-1,1	x	1,1	0,80	*	MerR family transcriptional regulator
SMU.678	-1	x	1,2	0,38		oxidoreductase
SMU.679	1	2	1,2	0,50		oxidoreductase
SMU.680	-1	x	1,2	0,44	*	gamma-carboxymuconolactone decarboxylase subunit
SMU.681	1,1	x	1,2	0,78	*	hypothetical protein
SMU.682	-1,3	x	-1,2	0,39		hypothetical protein
SMU.683	-1,2	x	-1,3	0,13		ATP-binding protein
SMU.684	-1,2	x	-1,2	0,36		hypothetical protein
SMU.685	-1,4	x	-1,0	0,94	*	hypothetical protein
SMU.687c	1,1	x	32,0	**0,00**	*	hypothetical protein
SMU.688	-1,1	x	1,1	0,56		hypothetical protein
SMU.689	-1,1	x	-1,2	0,20		hypothetical protein
SMU.690	-1,4	x	-1,5	**0,03**		hypothetical protein
SMU.691	-1,2	x	-1,6	**0,01**		peptidase T
SMU.692	-1,1	x	1,1	0,74		hypothetical protein
SMU.694c	1,3	x	-1,9	0,58	*	ferredoxin (4Fe-4S)
SMU.695	1,1	x	1,1	0,64		hypothetical protein
SMU.696	-1	x	1,2	0,26		cytidylate kinase
SMU.697	-1,2	x	1,1	0,64		translation initiation factor IF-3
SMU.698	-1,2	x	1,1	0,50		50S ribosomal protein L35
SMU.699	-1	x	1,1	0,65		50S ribosomal protein L20
SMU.700c	-1,4	x	-1,1	0,55		phosphoglycerate mutase
SMU.701c	-1,2	x	-1,0	0,94		hypothetical protein

SMU.702c	-1,1	x	-1,2	0,35		transcriptional regulator
SMU.703c	-1,2	x	-1,2	0,36		hypothetical protein
SMU.704c	-1,1	x	1,3	0,16		autolysin; amidase
SMU.706c	-1,2	x	1,2	0,53	*	hypothetical protein
SMU.707c	-1,1	x	-1,4	0,08		endolysin
SMU.709	-1,1	x	-1,5	**0,02**		hypothetical protein
SMU.711	-1	x	-1,6	**0,02**		hypothetical protein
SMU.712	-1,3	x	-1,4	**0,04**		phosphoenolpyruvate carboxylase
SMU.713	-1,1	x	-1,0	0,88		cell division protein FtsW
SMU.714	1,2	x	-1,1	0,58		elongation factor Tu
SMU.715	1,1	x	-1,2	0,36		triosephosphate isomerase
SMU.716	-1,2	x	-1,1	0,62		peptidoglycan branched peptide synthesis protein MurN
SMU.717	-1,2	x	-1,2	0,39		peptidoglycan branched peptide synthesis protein MurM
SMU.718c	-1,1	x	-1,2	0,44		hypothetical protein
SMU.719c	1	x	1,1	0,65		hypothetical protein
SMU.720	-1,3	x	-1,3	0,09		Na+/solute symporter
SMU.721	-1,4	x	-1,8	**0,01**		hypothetical protein
SMU.722	-1,2	x	-1,5	0,15	*	hypothetical protein
SMU.723	1	x	-1,3	0,12		cadmium-transporting ATPase
SMU.724	-1,1	x	-1,3	0,49	*	glycerophosphoryl diester phosphodiesterase
SMU.725c	-1	x	1,3	0,16		hypothetical protein
SMU.727	-1	x	1,3	0,25	*	transcriptional regulator
SMU.728	1,1	x	1,1	0,47		oxidoreductase
SMU.730	1,1	x	1,1	0,72	*	hypothetical protein
SMU.731	1	x	1,5	0,16		ABC transporter ATP-binding protein
SMU.732	-1,3	x	1,4	0,18	*	hypothetical protein
SMU.734	-1,2	x	-1,2	0,49	*	hypothetical protein
SMU.735	-1	x	1,1	0,74		hypothetical protein
SMU.737	1,1	x	1,5	**0,02**		hypothetical protein
SMU.738	-1,1	x	1,5	0,40	*	hypothetical protein
SMU.739c	-1	2	1,3	0,48	*	hypothetical protein
SMU.741	1	x	1,1	0,51		hypothetical protein
SMU.742	-1,1	x	1,1	0,48		hypothetical protein
SMU.743	-1	x	1,1	0,69		hypothetical protein
SMU.744	-1	x	1,0	0,86		cell division protein FtsY
SMU.745	-1,2	x	-1,4	**0,05**		drug-export protein; multidrug resistance protein
SMU.746c	1,1	2,1	1,4	0,05		hypothetical protein
SMU.747c	-1,1	x	1,3	0,14		permease
SMU.748	-1,1	2,1	-1,3	0,85	*	hypothetical protein
SMU.750c	-1,4	x	14,0	0,87	*	hypothetical protein
SMU.751	-1	x	-1,3	0,14		transcriptional accessory protein
SMU.752	-1,2	x	-1,1	0,71		hypothetical protein
SMU.753	1,9	x	2,4	**0,00**		hypothetical protein
SMU.754	1,2	x	1,4	**0,03**		HPr kinase/phosphorylase
SMU.755	1,1	x	1,5	**0,02**		prolipoprotein diacylglyceryl transferase
SMU.756	-1,1	x	1,7	**0,00**		hypothetical protein
SMU.757	-1,1	2,5	1,6	**0,04**		hypothetical protein
SMU.758c	1	x	1,1	0,81		hypothetical protein
SMU.759	1,1	x	1,5	**0,01**		protease
SMU.761	1,2	x	1,2	0,29		protease
SMU.764	1,2	x	1,2	0,34		alkyl hydroperoxide reductase
SMU.765	-1	x	1,3	0,10		alkyl hydroperoxide reductase
SMU.766	-1,1	4,1	-1,8	0,39	*	hypothetical protein
SMU.767	-1,2	2,6	1,1	0,71	*	transposase, ISSmu1

SMU.768c	-1,8	x	-2,0	0,22	*	hypothetical protein
SMU.769	**18,2**	19,2	111,8	**0,00**		hypothetical protein
SMU.770c	-1,3	x	-5,6	**0,00**	*	manganese transporter
SMU.771c	-1,3	7,1	1,2	0,69	*	hypothetical protein
SMU.772	**4,9**	x	22,8	**0,00**		glucan-binding protein D
SMU.773c	-1,2	x	-1,3	0,14		lysyl-tRNA synthetase
SMU.774	-1	x	1,8	**0,00**		hypothetical protein
SMU.775c	-1,1	x	1,1	0,50		hypothetical protein
SMU.776	1	x	1,0	0,81		hypothetical protein
SMU.777	1,1	x	1,1	0,56		3-dehydroquinate dehydratase
SMU.778	1	x	1,1	0,43		shikimate 5-dehydrogenase
SMU.779	1,2	x	1,1	0,70		3-dehydroquinate synthase
SMU.780	1,2	x	1,0	0,92		chorismate synthase
SMU.781	1,1	x	1,1	0,57		prephenate dehydrogenase
SMU.782	1	x	1,1	0,64		hypothetical protein
SMU.784	-1,1	x	-1,1	0,42		3-phosphoshikimate 1-carboxyvinyltransferase
SMU.785	-1,2	x	-1,2	0,31		shikimate kinase
SMU.786	-1,3	x	-1,2	0,33		prephenate dehydratase
SMU.787	-1,1	x	-1,1	0,44		transcriptional regulator
SMU.788	-1	x	-1,2	0,39		RNA methyltransferase
SMU.789	-1,1	x	1,0	0,85		hypothetical protein
SMU.790	-1,1	3,7	3,2	**0,03**	*	hypothetical protein
SMU.791c	-1,1	3,1	3,8	0,14	*	hypothetical protein
SMU.793	-1,1	x	-1,1	0,77		hypothetical protein
SMU.794	-1,1	x	-1,0	0,86		hypothetical protein
SMU.795	-1,1	x	1,0	0,90		esterase
SMU.796	-1	x	-1,1	0,73		hypothetical protein
SMU.797	-1,2	x	-1,3	0,43	*	hypothetical protein
SMU.798c	1,1	x	1,8	0,42	*	hypothetical protein
SMU.799c	1,1	x	1,7	0,08	*	hypothetical protein
SMU.800	-1,1	x	1,2	0,30		hypothetical protein
SMU.801	1,1	x	1,1	0,44		GTP-binding protein
SMU.802	1,1	x	1,1	0,62		hypothetical protein
SMU.803c	1,2	x	1,8	**0,00**		ABC transporter ATP-binding protein
SMU.804	-1,1	x	1,8	**0,05**		hypothetical protein
SMU.805c	1	x	-1,6	0,36		amino acid ABC transporter ATP-binding protein
SMU.806c	1	x	-1,5	0,38		glutamine ABC transporter permease
SMU.807	1,2	2,1	2,3	**0,00**		hypothetical protein
SMU.809	-1	x	1,4	**0,03**		excinuclease ABC subunit B
SMU.811	-1,2	2,2	1,8	**0,02**		hypothetical protein
SMU.812	-1,1	3,4	-1,1	1,00	*	hypothetical protein
SMU.813	-1,1	2,1	1,5	**0,05**		transcriptional regulator
SMU.814	-1	x	1,0	0,85		MutT-like protein
SMU.815	-1,3	x	-1,1	0,65		amino acid ABC transporter substrate-binding protein
SMU.816	-1	x	-1,1	0,43		transaminase
SMU.817	-1,3	x	-1,1	0,60		amino acid ABC transporter substrate-binding protein
SMU.818	-1,1	x	-1,4	0,10		30S ribosomal protein S21
SMU.819	1	x	-1,1	0,66		large conductance mechanosensitive channel
SMU.820	1,1	2,4	1,4	0,06		hypothetical protein
SMU.821	1,1	2,1	1,4	**0,04**		DNA primase
SMU.822	1,1	x	1,3	0,17		RNA polymerase sigma factor RpoD
SMU.823	1	x	1,3	0,11		hypothetical protein
SMU.824	-1	x	1,4	0,07		dTDP-4-keto-L-rhamnose reductase
SMU.825	1,1	x	1,2	0,20		RgpAc; glycosyltransferase

SMU.826	1,1	x	1,3	0,09		rhamnosyltransferase
SMU.827	-1,1	x	1,1	0,45		polysaccharide ABC transporter permease
SMU.828	1,1	x	1,1	0,49		polysaccharide ABC transporter ATP-binding protein
SMU.829	-1,1	x	1,2	0,30		glycosyltransferase
SMU.830	-1	x	1,1	0,72		RgpFc protein
SMU.831	-1,2	x	1,0	0,90		hypothetical protein
SMU.832	-1,2	x	1,1	0,43		hypothetical protein
SMU.833	1,1	x	1,2	0,31		glycosyltransferase
SMU.834	-1	2,1	1,4	0,07		hypothetical protein
SMU.835	1,5	2,4	2,5	**0,00**		hypothetical protein
SMU.836	**74,8**	71	172,4	**0,00**		hypothetical protein
SMU.837	**32**	29,7	82,5	**0,00**		reductase
SMU.838	**2,4**	5,8	29,3	**0,00**		glutathione reductase
SMU.839	-1,1	x	-1,6	**0,02**		folyl-polyglutamate synthetase
SMU.840c	1,1	x	-1,1	0,87		hypothetical protein
SMU.841	1	x	1,0	0,92		aminotransferase
SMU.842	-1	x	-1,1	0,60		thiamine biosynthesis protein ThiI
SMU.843	1,1	x	-1,0	0,80		hypothetical protein
SMU.844	1	2	1,0	0,97		hypothetical protein
SMU.845	-1,1	2	1,2	0,52		hypothetical protein
SMU.846	-1	x	-1,1	0,62		50S ribosomal protein L21
SMU.847c	-1,1	x	3,2	0,50	*	hypothetical protein
SMU.848	-1,1	x	-1,3	0,15		hypothetical protein
SMU.849	-1,1	x	-1,1	0,44		50S ribosomal protein L27
SMU.850	-1	x	1,1	0,56		hypothetical protein
SMU.851	-1,1	x	1,1	0,50		hypothetical protein
SMU.852	1,2	x	1,3	0,19		transcriptional regulator; CpsY-like protein
SMU.853	-1,1	x	1,3	0,23		lipoprotein signal peptidase
SMU.854	1	x	1,2	0,39		pseudouridylate synthase
SMU.855	-1	x	1,4	0,07		hypothetical protein
SMU.856	1,2	x	1,7	**0,02**		bifunctional pyrimidine regulatory protein PyrR/uracil phosphoribosyltransferase
SMU.857	1,1	x	1,5	**0,02**		uracil permease
SMU.858	1,1	x	1,3	0,17		aspartate carbamoyltransferase
SMU.859	1,2	x	1,3	0,12		carbamoyl phosphate synthase small subunit
SMU.860	1,2	x	1,2	0,26		carbamoyl phosphate synthase large subunit
SMU.862	1,1	x	2,7	**0,01**		permease
SMU.863	1,1	2,4	2,5	**0,00**		ABC transporter ATP-binding protein
SMU.864	1,2	2,4	2,9	**0,00**		ABC transporter permease
SMU.865	-1,1	x	1,1	0,79		30S ribosomal protein S16
SMU.866	-1,1	x	-1,1	0,79		hypothetical protein
SMU.867	-1,1	x	1,4	0,10		16S rRNA-processing protein RimM
SMU.868	1	x	1,1	0,66		tRNA (guanine-N(1)-)-methyltransferase
SMU.869	-1,2	x	-1,2	0,40		thioredoxin reductase
SMU.870	1,2	x	1,1	0,91		sugar metabolism transcriptional regulator
SMU.871	-1	x	-1,3	0,13		fructose-1-phosphate kinase
SMU.872	-1	x	-1,5	**0,02**		PTS system fructose-specific transporter subunit IIABC
SMU.873	1,1	x	1,6	**0,03**		5-methyltetrahydropteroyltriglutamate/homocysteine S-methyltransferase
SMU.874	1,2	x	1,6	0,09		bifunctional homocysteine S-methyltransferase/5,10-methylenetetrahydrofolate reductase
SMU.875c	1	x	3,0	0,34	*	transposase, IS150-like
SMU.876	-1,1	x	1,3	0,55	*	MSM operon regulatory protein
SMU.877	1,4	x	1,6	**0,02**		alpha-galactosidase
SMU.878	1,4	x	1,8	**0,00**		multiple sugar-binding ABC transporter, sugar-binding protein MsmE
SMU.879	1,4	x	1,9	**0,00**		multiple sugar-binding ABC transporter permease MsmF

SMU.880	1,3	x	1,8	**0,00**		multiple sugar-binding ABC transporter permease MsmG
SMU.881	1,6	x	1,9	**0,00**		sucrose phosphorylase GtfA
SMU.882	1,6	x	1,8	**0,01**		multiple sugar-binding ABC transporter ATP-binding protein, MsmK
SMU.883	1,7	x	1,9	**0,00**		dextran glucosidase DexB
SMU.885	1,1	x	1,1	0,61		LacI family transcriptional repressor
SMU.886	1,4	x	3,1	**0,00**		galactokinase
SMU.887	1,5	x	1,7	**0,01**		galactose-1-phosphate uridylyltransferase
SMU.888	1,1	x	-1,1	0,62		UDP-galactose 4-epimerase GalE
SMU.889	1,1	x	1,1	0,42		penicillin-binding protein, class C; fmt-like protein
SMU.890	-1,3	x	1,2	0,39		hypothetical protein
SMU.891	-1,2	x	1,2	0,26		type I restriction-modification system DNA methylase
SMU.892	-1,2	x	1,1	0,44		type I restriction-modification system, specificity determinant
SMU.893	-1,2	x	1,1	0,65		anticodon nuclease
SMU.895	-1,1	x	1,8	0,05	*	DNA-damage-inducible protein
SMU.896	-1,3	x	2,2	**0,01**	*	hypothetical protein
SMU.897	-1,2	x	1,5	**0,02**		type I restriction-modification system, helicase subunits
SMU.898	-1,2	x	1,1	0,61		hypothetical protein
SMU.899	-1,2	x	-1,1	0,45		hypothetical protein
SMU.900	1	x	-1,2	0,24		dihydrodipicolinate reductase
SMU.901	1	x	1,0	0,97		tRNA CCA-pyrophosphorylase
SMU.902	1,1	x	1,0	0,90		ABC transporter ATP-binding protein
SMU.905	1,2	2,8	1,3	0,10		ABC transporter ATP-binding protein
SMU.906	1,1	2,9	1,3	0,13		ABC transporter ATP-binding protein
SMU.909	-1	2	1,3	0,21		permease
SMU.910	-1,3	x	-1,5	0,29		glucosyltransferase-S
SMU.911c	1,2	x	-1,1	0,68		hypothetical protein
SMU.913	-1,1	x	1,3	0,38		glutamate dehydrogenase
SMU.914c	1,1	x	-2,7	**0,00**		hypothetical protein
SMU.915c	-1,1	x	-3,0	**0,00**		7-cyano-7-deazaguanine reductase
SMU.916c	1,1	x	-3,2	**0,00**		hypothetical protein
SMU.917c	1,2	x	-2,9	**0,00**		6-pyruvoyl tetrahydrobiopterin synthase
SMU.919c	1,1	x	-2,8	**0,00**		ATPase, confers aluminum resistance
SMU.921	-1,2	x	1,0	0,87		transcriptional regulator
SMU.922	1,1	x	1,0	0,89		ABC transporter ATP-binding protein
SMU.923	1,2	x	1,0	0,84		ABC transporter ATP-binding protein
SMU.924	-1,3	x	-1,5	0,26		lipid hydroperoxide peroxidase
SMU.925	**10,3**	x	6,0	**0,00**		hypothetical protein
SMU.926	**3,7**	x	1,7	**0,01**		GTP-pyrophosphokinase
SMU.927	**3,4**	x	2,0	**0,00**		response regulator
SMU.928	**2,7**	x	1,7	**0,01**		histidine kinase
SMU.929c	-1,2	2,2	-1,3	0,59		hypothetical protein
SMU.930c	-1,1	x	1,6	0,30	*	transcriptional regulator
SMU.932	-1,2	3,2	3,4	**0,00**	*	hypothetical protein
SMU.933	-1	2,8	2,7	**0,04**	*	amino acid ABC transporter substrate-binding protein
SMU.934	1	2,1	2,0	0,13	*	amino acid ABC transporter permease
SMU.935	-1,1	x	2,5	0,11	*	amino acid ABC transporter permease
SMU.936	-1	x	3,2	**0,02**	*	amino acid ABC transporter ATP-binding protein
SMU.937	-1,2	x	-1,1	0,63		mevalonate diphosphate decarboxylase
SMU.938	-1,1	x	-1,1	0,67		phosphomevalonate kinase
SMU.939	1	x	-1,0	0,77		isopentenyl pyrophosphate isomerase
SMU.940c	1	x	1,2	0,45		hemolysin III
SMU.941c	1,2	x	1,2	0,45		hypothetical protein
SMU.942	1	x	1,0	0,85		hydroxymethylglutaryl-CoA reductase
SMU.943c	1,1	x	-1,2	0,31		hydroxymethylglutaryl-CoA synthase

SMU.944	-1,1	x	-1,4	0,10		thymidylate synthase
SMU.946	1,1	x	-1,6	**0,02**		permease
SMU.947	1,1	x	1,3	0,17		dihydrofolate reductase
SMU.948	1,2	x	-1,2	0,90		hypothetical protein
SMU.949	1	x	1,2	0,27		ATP-dependent protease ATP-binding subunit ClpX
SMU.950	1	x	1,2	0,24		GTP-binding protein YsxC
SMU.951	1	x	1,7	**0,00**		amino acid permease
SMU.952	1	x	1,9	**0,00**		homocysteine methyltransferase
SMU.953c	1,1	x	-1,3	0,32	*	transcriptional regulator/aminotransferase
SMU.954	-1,3	x	2,5	**0,02**		pyridoxamine kinase
SMU.955	-1,2	x	2,3	**0,01**		hypothetical protein
SMU.956	1,1	x	1,5	0,56		Clp-like ATP-dependent protease, ATP-binding subunit
SMU.957	-1,2	x	-1,2	0,24		50S ribosomal protein L10
SMU.958	-1	-2,2	-1,2	0,21		hypothetical protein
SMU.959c	-1	x	1,3	0,89	*	hypothetical protein
SMU.960	-1	x	-1,2	0,36		50S ribosomal protein L7/L12
SMU.961	1,1	x	2,2	**0,00**		hypothetical protein
SMU.962	-1,1	x	2,1	**0,00**		dehydrogenase
SMU.963c	-1,1	x	-1,0	0,99		deacetylase
SMU.965	-1,1	x	-1,6	**0,01**		homoserine dehydrogenase
SMU.966	-1,1	x	-1,5	**0,02**		homoserine kinase
SMU.967	-1,1	x	-1,4	0,07		folyl-polyglutamate synthetase
SMU.968	-1	x	-1,3	0,14		GTP cyclohydrolase I
SMU.969	1	x	-1,4	**0,04**		dihydropteroate synthase
SMU.970	1	x	-1,3	0,16		dihydroneopterin aldolase
SMU.971	1,1	x	-1,3	0,17		2-amino-4-hydroxy-6-hydroxymethyldihydropteridine pyrophosphokinase
SMU.972	-1,1	x	-1,0	0,85		UDP-N-acetylenolpyruvoylglucosamine reductase
SMU.973	1	x	-1,0	0,96		spermidine/putrescine ABC transporter ATP-binding protein
SMU.974	1	x	-1,1	0,84		spermidine/putrescine ABC transporter permease
SMU.975	-1,1	2,4	1,0	0,85		spermidine/putrescine ABC transporter permease
SMU.976	1	x	1,1	0,74		ABC transporter periplasmic spermidine/putrescine-binding protein
SMU.977	1	2,6	1,4	0,07		transcriptional antiterminator LicT (fragment)
SMU.980	1,2	x	2,4	**0,00**		PTS system beta-glucoside-specific transporter subunit II
SMU.981	1,2	3	3,3	**0,01**		BglB fragment
SMU.982	1,1	4,2	3,0	**0,01**	*	BglB fragment
SMU.983	-1,2	x	-1,1	0,84	*	transcriptional regulator
SMU.984	-1,2	x	-1,6	0,17	*	hypothetical protein
SMU.985	-1,1	x	-1,2	0,38		beta-glucosidase
SMU.986c	-1,2	x	-1,2	0,71	*	hypothetical protein
SMU.987	-1,3	-2	-1,9	**0,00**		cell wall-associated protein WapA
SMU.988	-1,3	x	-1,3	0,10		cardiolipin synthase
SMU.989	1	x	1,6	**0,01**		aspartate-semialdehyde dehydrogenase
SMU.990	1,1	x	1,4	0,06		dihydrodipicolinate synthase
SMU.991	-1,1	x	1,5	0,05		ribonucleotide reductase
SMU.992	-1,2	2,8	1,1	0,81		hypothetical protein
SMU.993	-1,1	2,6	-1,1	0,72		GTPase YlqF
SMU.994	-1,1	2,2	-1,1	0,74		ribonuclease HII
SMU.995	-1,2	x	-1,0	0,96		ferrichromeABC transporter permease
SMU.996	-1,3	2,4	1,1	0,69		ferrichromeABC transporter permease
SMU.997	-1,1	2,2	1,3	0,19		inorganic ion ABC transporter ATP-binding protein
SMU.998	-1,2	x	1,1	0,61		ABC transporter periplasmic ferrichrome-binding protein
SMU.999	-1,3	x	-1,3	0,90	*	hypothetical protein
SMU.1000	-1,2	-3	1,9	0,69	*	hypothetical protein
SMU.1001	**45**	525,1	533,7	**0,00**		DNA processing Smf protein

SMU.1002	**6,5**	8,9	41,8	**0,00**		DNA topoisomerase I
SMU.1003	**5,8**	10,2	30,0	**0,00**		tRNA (uracil-5-)-methyltransferase Gid
SMU.1004	1,2	x	2,1	**0,00**		glucosyltransferase-I
SMU.1005	-1,1	x	-1,8	**0,00**		glucosyltransferase-SI
SMU.1006	-1,3	x	-1,2	0,29		ABC transporter ATP-binding protein
SMU.1007	-1,4	x	-1,1	0,76		ABC transporter permease
SMU.1008	-1,1	x	-1,2	0,45		response regulator
SMU.1009	-1,3	x	-1,4	0,11		histidine kinase
SMU.1010	-1,3	x	1,0	0,92	*	citrate lyase ligase
SMU.1011	1	x	-1,0	0,84	*	CitG protein
SMU.1012c	-1,1	x	-1,1	0,73	*	transcriptional regulator
SMU.1013c	-1,1	x	1,3	0,39	*	Mg2+/citrate transporter
SMU.1014	-1,3	x	-1,4	0,08		hypothetical protein
SMU.1016	-1,2	x	-1,7	**0,02**		acetyl-CoA carboxylase biotin carboxyl carrier protein subunit
SMU.1017	-1,2	x	-1,3	0,23		oxaloacetate decarboxylase, sodium ion pump subunit
SMU.1018	-1,2	x	-1,6	0,23	*	hypothetical protein
SMU.1019	-1,1	x	-1,5	0,08		citrate lyase subunit gamma
SMU.1020	-1,2	x	-1,4	0,05		citrate lyase CilB, cityl-CoA lyase subunit beta
SMU.1021	-1,1	x	-1,5	**0,03**		citrate lyase, alfa subunit
SMU.1022	-1,2	x	-1,5	0,07		2-(5''-triphosphoribosyl)-3'-dephosphocoenzyme-A synthase
SMU.1023	-1	x	-1,4	0,06		oxaloacetate decarboxylase
SMU.1024c	-1,2	2	-1,0	1,00	*	transposase fragment
SMU.1025	-1	x	1,1	0,83	*	transcriptional regulator
SMU.1026	-1,1	x	-1,6	0,48	*	hypothetical protein
SMU.1027	-1,4	x	1,2	0,78	*	transcriptional regulator
SMU.1028	-1,2	x	1,4	0,13		hydrolase
SMU.1029	-1,1	x	77,6	0,34	*	hypothetical protein
SMU.1030	-1,1	x	69,9	0,37	*	polyribonucleotide nucleotidyltransferase
SMU.1031	1,1	x	2,9	0,98	*	transposon excisionase; Tn916 ORF1-like
SMU.1032	-1,1	x	-1,5	0,19	*	transposon integrase; Tn916 ORF3-like
SMU.1034c	-1,1	x	1,3	0,12		site-specific tyrosine recombinase XerS
SMU.1035	-1	x	1,4	0,38	*	ABC transporter ATP-binding protein
SMU.1036	1	x	1,9	0,16	*	hypothetical protein
SMU.1037c	-1,1	x	1,4	0,09		histidine kinase
SMU.1038c	-1,1	x	1,5	**0,04**		response regulator
SMU.1039c	-1,2	x	1,7	**0,01**		lipopolysaccharide glycosyltransferase
SMU.1040c	-1,1	x	2,1	**0,00**		oxidoreductase
SMU.1041	-1,3	2,4	1,1	0,70		ABC transporter ATP-binding protein
SMU.1042	-1,3	2,6	-1,1	0,87		hypothetical protein
SMU.1043c	1,5	2	2,9	**0,00**		phosphotransacetylase
SMU.1044c	1,4	2,6	3,9	**0,00**		pseudouridylate synthase
SMU.1045c	1,2	2,6	4,1	**0,00**		inorganic polyphosphate/ATP-NAD kinase
SMU.1046c	1,5	2,8	4,8	**0,00**		GTP pyrophosphokinase
SMU.1047c	1,1	2,1	5,5	**0,00**		hypothetical protein
SMU.1048	-1,3	2,1	-2,4	**0,00**	*	hypothetical protein
SMU.1050	-1,2	x	-4,1	**0,00**		ribose-phosphate pyrophosphokinase
SMU.1051	-1,5	x	-4,3	**0,00**		iron-sulfur cofactor synthesis protein; NifS family
SMU.1052	-1,7	x	-4,0	0,08	*	hypothetical protein
SMU.1053	-1,8	10,5	-6,2	**0,00**	*	redox-sensing transcriptional repressor Rex
SMU.1054	-1,6	20,3	-18,8	**0,00**	*	glutamine amidotransferase
SMU.1055	**23**	27,5	55,1	**0,00**		hypothetical protein
SMU.1056	-1,1	3,4	-5,0	0,14	*	hypothetical protein
SMU.1057	-1,3	x	-1,2	0,38		hypothetical protein
SMU.1058	1	x	-1,4	0,08		hypothetical protein

SMU.1059	-2	x	-1,2	0,53		hypothetical protein
SMU.1060	-1,2	x	-1,3	0,10		signal recognition particle protein
SMU.1061	-1,2	x	-1,2	0,53		DNA-binding protein
SMU.1062	-1,3	x	-2,4	**0,00**		ABC transporter proline/glycine betaine permease
SMU.1063	-1,3	x	-2,2	**0,00**		proline/glycine betaine ABC transporter ATP-binding protein
SMU.1064c	-1,1	x	-1,6	**0,03**		GntR family transcriptional regulator
SMU.1065c	-1	x	-1,3	0,18		GntR family transcriptional regulator
SMU.1066	-1,1	x	-1,3	0,08		GMP synthase
SMU.1067c	1,1	2,6	1,5	0,06		ABC transporter permease
SMU.1068c	1,1	2,2	1,5	**0,03**		ABC transporter ATP-binding protein
SMU.1069c	1,1	2,2	1,5	0,31		hypothetical protein
SMU.1070c	1,4	2,4	1,6	**0,03**		hypothetical protein
SMU.1071c	1,1	x	-1,1	0,86		hypothetical protein
SMU.1072c	-1,1	x	-3,7	**0,00**	*	acetyltransferase
SMU.1073	-1,3	-2,5	-3,6	**0,00**		formate--tetrahydrofolate ligase
SMU.1074	1,2	x	1,5	**0,04**		phosphopantothenate--cysteine ligase
SMU.1075	-1	x	1,2	0,26		phosphopantothenoylcysteine decarboxylase
SMU.1076	-1,1	x	-1,0	0,93		hypothetical protein
SMU.1077	1,1	x	-1,3	0,16		phosphoglucomutase
SMU.1078c	-1	x	1,1	0,50		ABC transporter ATP-binding protein
SMU.1079c	-1,1	x	-1,0	0,98		ABC transporter ATP-binding protein
SMU.1080c	-1	2,1	-1,2	0,41		transposon-like protein
SMU.1081c	-1,3	x	-1,2	0,23		hypothetical protein
SMU.1082	-1,1	x	-1,4	0,05		serine hydroxymethyltransferase
SMU.1083c	-1,1	x	-1,4	0,06		hypothetical protein
SMU.1084	-1,1	x	-1,2	0,23		N5-glutamine S-adenosyl-L-methionine-dependent methyltransferase
SMU.1085	-1,1	x	-1,3	0,09		peptide chain release factor 1
SMU.1086	-1,1	x	-1,2	0,34		thymidine kinase
SMU.1087	-1,2	x	-1,5	0,11	*	4-oxalocrotonate tautomerase
SMU.1088	-1	x	-1,2	0,35		thiamine biosynthesis lipoprotein
SMU.1089	-1,1	x	-1,4	0,06		hypothetical protein
SMU.1090	-1	x	-1,3	0,10		hypothetical protein
SMU.1091	-1,1	2,6	1,2	0,56		cell wall protein, WapE
SMU.1093	-1,1	2,1	2,2	**0,00**		ABC transporter permease
SMU.1094	1	x	2,6	**0,04**	*	ABC transporter ATP-binding protein
SMU.1095	-1,3	x	1,0	0,93		choline ABC transporter, osmoprotectant binding protein
SMU.1096	-1,1	x	1,0	0,90		choline transporter ABC transporter ATP-binding protein
SMU.1097c	-1,1	x	-1,0	0,97		transcriptional regulator
SMU.1098c	-1,2	x	-1,1	0,71		oxidoreductase
SMU.1100c	-1,3	x	-1,6	**0,01**		permease
SMU.1102	-1	x	1,3	0,22		6-phospho-beta-glucosidase
SMU.1104c	1,1	x	1,5	0,11		phosphoglycerate mutase
SMU.1105c	1	x	1,1	0,54		phosphoglycerate mutase
SMU.1106c	1	x	1,4	0,16		phosphoglycerate mutase
SMU.1107c	1	x	1,4	0,09		hypothetical protein
SMU.1108c	1	x	1,5	**0,05**		hypothetical protein
SMU.1109c	1,4	x	2,0	**0,00**		permease
SMU.1111c	1,6	x	2,4	**0,00**		hypothetical protein
SMU.1112c	1,1	x	1,3	0,28		hypothetical protein
SMU.1113	1,1	x	-1,1	0,56		sortase
SMU.1114	1,1	x	1,1	0,76		DNA gyrase subunit A
SMU.1115	1	x	-1,2	0,37		L-lactate dehydrogenase
SMU.1116c	-1	x	-1,1	0,77		hypothetical protein
SMU.1117	-1	x	1,0	0,99		NADH oxidase (H2O-forming)

SMU.1118c	-1,1	x	-1,1	0,50		ABC sugar transporter, permease
SMU.1119c	-1,1	x	-1,2	0,26		sugar ABC transporter permease
SMU.1120	-1,1	x	-1,4	0,06		sugar ABC transporter ATP-binding protein
SMU.1121c	-1,1	x	-1,3	0,09		ABC transporter
SMU.1122	-1,1	x	-1,1	0,35		cytidine deaminase
SMU.1123	-1,1	x	-1,2	0,23		deoxyribose-phosphate aldolase
SMU.1124	-1	x	-1,2	0,24		pyrimidine-nucleoside phosphorylase
SMU.1125c	-1	x	-1,0	0,84		hypothetical protein
SMU.1126	-1,4	x	-1,2	0,60	*	pantothenate kinase
SMU.1127	-1,1	x	1,0	0,86		30S ribosomal protein S20
SMU.1128	-1,2	x	1,2	0,23		histidine kinase sensor CiaH
SMU.1129	-1,2	x	1,2	0,33		response regulator CiaR
SMU.1131c	-1	x	1,7	**0,04**		hypothetical protein
SMU.1132	1	2	1,3	0,16		aminopeptidase
SMU.1133	-1,2	x	-1,1	0,65		phosphate transport system regulatory protein
SMU.1134c	-1,1	x	1,1	0,87		phosphate transporter ATP-binding protein
SMU.1135	-1,2	x	-1,1	0,64		phosphate transporter ATP-binding protein
SMU.1136	-1,1	x	-1,2	0,30		phosphate ABC transporter permease
SMU.1137	-1,2	x	-1,2	0,38		phosphate ABC transporter permease
SMU.1138	-1	x	-1,1	0,66		phosphate ABC transporter substrate-binding protein
SMU.1139c	1	x	1,3	0,21		methylase
SMU.1140c	-1	x	1,0	0,93		hypothetical protein
SMU.1141c	1	x	1,1	0,82		hypothetical protein
SMU.1142c	-1	x	-1,1	0,62		transcriptional regulator Spx
SMU.1143c	1	x	1,3	0,17		bifunctional riboflavin kinase/FMN adenylyltransferase
SMU.1144	1,1	x	1,5	**0,05**		tRNA pseudouridine synthase B
SMU.1145c	1,4	x	2,4	**0,00**		histidine kinase
SMU.1146c	1,4	x	2,7	**0,00**		response regulator
SMU.1147c	1,1	x	8,2	**0,00**	*	hypothetical protein
SMU.1148	1	2,8	15,4	**0,00**	*	transporter, ATP-binding protein; bacteriocin immunity protein
SMU.1149	1	2,2	5,5	**0,02**	*	transporter, trans-membrane domain bacteriocin immunity protein
SMU.1150	1	x	7,5	**0,00**	*	transporter, trans-membrane domain bacteriocin immunity protein
SMU.1151c	1,1	x	1,1	0,45		hypothetical protein
SMU.1152c	1,1	x	1,2	0,63	*	hypothetical protein
SMU.1153c	1	x	1,3	0,35		hypothetical protein
SMU.1154c	1	x	-1,1	0,69		hypothetical protein
SMU.1155	1	x	4,4	0,12	*	hypothetical protein
SMU.1156c	1	x	-1,1	0,57	*	hypothetical protein
SMU.1157c	-1,3	x	1,0	0,83		hypothetical protein
SMU.1158c	-1,2	x	-1,3	0,25		hypothetical protein
SMU.1159c	-1,2	x	-1,6	0,35	*	hypothetical protein
SMU.1160c	-1,2	x	1,1	0,80	*	hypothetical protein
SMU.1161c	-1,1	x	1,9	0,08	*	hypothetical protein
SMU.1163c	-1	x	-1,3	0,18		ABC transporter ATP-binding protein
SMU.1164c	-1	x	-1,2	0,32		ABC transporter ATP-binding protein
SMU.1165c	-1	x	-1,2	0,50	*	transcriptional regulator
SMU.1166c	-1	3,4	1,5	**0,03**		ABC transporter permease
SMU.1167c	1,1	3,1	1,5	0,12		ABC transporter ATP-binding protein
SMU.1168	-1,1	2	-1,3	0,44	*	transcriptional regulator
SMU.1169c	-1,1	2,5	1,0	0,89		thioredoxin
SMU.1170	-1,2	2,3	-1,1	0,78		cytochrome C biogenesis protein
SMU.1171c	-1,1	x	1,2	0,31		hypothetical protein
SMU.1172c	1,4	2,5	1,2	0,35		hypothetical protein
SMU.1173	1,3	2,6	1,2	0,43		O-acetylhomoserine sulfhydrylase

SMU.1174	1,2	2,8	-1,1	0,75		ATP-dependent DNA helicase
SMU.1175	-1,3	x	-1,0	0,86		sodium/amino acid (alanine) symporter
SMU.1176	-1,2	x	-1,3	0,22		cation efflux transporter
SMU.1177c	-1,3	x	-1,2	0,44		ABC transporter glutamine binding protein
SMU.1178c	-1,2	x	-1,1	0,43		amino acid ABC transporter ATP-binding protein
SMU.1179c	-1,1	x	-1,2	0,40		amino acid ABC transporter permease
SMU.1180	-1,1	x	-1,1	0,52		alkylphosphonate uptake protein
SMU.1182	-1	x	1,1	0,66		mannitol-1-phosphate 5-dehydrogenase
SMU.1183	1,1	x	1,1	0,76		PTS system mannitol-specific transporter subunit IIA
SMU.1184c	-1,1	x	1,2	0,46		transcriptional regulator, antiterminator
SMU.1185	1,1	x	1,7	0,06		PTS system mannitol-specific transporter subunit IIBC
SMU.1187	-1	x	-1,7	0,07		glucosamine--fructose-6-phosphate aminotransferase
SMU.1188	-1,3	x	1,3	0,14		signal peptidase
SMU.1189c	1,1	x	1,4	0,15		hypothetical protein
SMU.1190	1,1	x	-1,0	0,83		pyruvate kinase
SMU.1191	1,2	x	-1,2	0,35		6-phosphofructokinase
SMU.1192	1,1	2,4	1,6	**0,03**		DNA polymerase III DnaE
SMU.1193	1,1	x	2,0	**0,05**		transcriptional regulator
SMU.1194	1	x	2,1	0,08		ABC transporter ATP-binding protein
SMU.1195	-1,1	x	1,8	0,07		permease
SMU.1196c	-1,1	x	1,5	**0,02**		hypothetical protein
SMU.1197	1,2	x	2,3	**0,00**		hypothetical protein
SMU.1200	-1,1	x	-1,2	0,32		30S ribosomal protein S1
SMU.1201c	-1,2	x	-1,0	0,95	*	hypothetical protein
SMU.1203	1	x	1,1	0,57		branched-chain amino acid aminotransferase
SMU.1204	1	x	-1,1	0,42		DNA topoisomerase IV subunit A
SMU.1205c	-1,3	x	-1,7	0,45		hypothetical protein
SMU.1206c	-1,1	x	1,0	0,86		hypothetical protein
SMU.1207	-1	x	-1,2	0,39		hypothetical protein
SMU.1208c	-1	x	-1,2	0,22		hypothetical protein
SMU.1209c	-1,3	x	-1,3	0,18		hypothetical protein
SMU.1210	-1,1	x	-1,2	0,26		DNA topoisomerase IV subunit B
SMU.1211	-1,2	x	-1,8	**0,00**		glycerol-3-phosphate acyltransferase PlsY
SMU.1213c	-1	x	-1,3	0,09		5'-nucleotidase
SMU.1214	-1	x	-1,2	0,28		dihydroorotase
SMU.1215	-1	x	-1,2	0,25		uracil-DNA glycosylase
SMU.1216c	1	x	-1,2	0,22		amino acid ABC transporter permease
SMU.1217c	-1,2	x	1,5	**0,04**		ABC transporter amino acid binding protein
SMU.1218	1,1	x	1,2	0,22		amidase
SMU.1219c	1,1	x	1,1	0,61		hypothetical protein
SMU.1220c	-1	x	1,3	0,21		hypothetical protein
SMU.1221	1,1	x	1,3	0,14		orotate phosphoribosyltransferase
SMU.1222	1,2	x	1,2	0,39		orotidine 5'-phosphate decarboxylase
SMU.1223	1,1	x	1,4	0,08		dihydroorotate dehydrogenase 1B
SMU.1224	1,1	x	1,8	**0,01**		dihydroorotate dehydrogenase electron transfer subunit
SMU.1225	-1	x	-1,3	0,14		transcriptional regulator
SMU.1226c	-1,2	x	-1,1	0,59		hypothetical protein
SMU.1227	1	x	-1,2	0,30		purine nucleoside phosphorylase
SMU.1228c	-1,1	x	-1,2	0,34		glutamine amidotransferase
SMU.1229	-1	x	-1,3	0,09		purine nucleoside phosphorylase
SMU.1230c	-1,2	x	-1,2	0,30		hypothetical protein
SMU.1231c	1,1	x	-1,4	0,09		hypothetical protein
SMU.1232c	-1,1	x	-1,3	0,24		hypothetical protein
SMU.1233	1	x	-1,3	0,17		phosphopentomutase

SMU.1234	1,1	x	-1,3	0,15		ribose-5-phosphate isomerase A
SMU.1235	1,1	x	1,4	0,10		tRNA modification GTPase TrmE
SMU.1236c	-1,2	x	-1,1	0,71	*	hypothetical protein
SMU.1237c	-1,2	x	-1,6	0,32	*	hypothetical protein
SMU.1238c	1,1	x	-1,1	0,57		hypothetical protein
SMU.1239	1,1	x	-1,1	0,71		dipeptidase PepV
SMU.1240c	1,1	x	-1,0	0,95		nitroreductase
SMU.1241	1,1	x	1,0	0,92		excinuclease ABC subunit C
SMU.1243	-1,1	x	1,6	0,13	*	low temperature requirement A protein
SMU.1245c	1	x	1,0	0,97		hypothetical protein
SMU.1246c	-1	x	1,1	0,68		transcriptional regulator
SMU.1247	1	x	-1,3	0,10		enolase
SMU.1249c	-1,3	x	-1,0	0,96		hypothetical protein
SMU.1250c	-1,3	x	1,2	0,51	*	hypothetical protein
SMU.1251	1,1	x	1,9	0,05	*	hypothetical protein
SMU.1252	1,1	x	1,2	0,36		glycerate kinase
SMU.1253c	1	x	1,3	0,33		hypothetical protein
SMU.1254	1,1	x	-1,4	0,23	*	hypothetical protein
SMU.1255c	-1,2	x	1,1	0,84		hypothetical protein
SMU.1256c	1	2,1	1,1	0,55		hypothetical protein
SMU.1257c	1	x	1,2	0,46		hypothetical protein
SMU.1258c	-1,1	x	-1,0	0,91	*	restriction endonuclease
SMU.1259	1,1	x	2,5	0,58	*	restriction endonuclease
SMU.1260c	-1,4	x	-1,2	0,50	*	hypothetical protein
SMU.1261c	-1,4	x	-1,5	0,07		phosphoribosyl-ATP pyrophosphohydrolase
SMU.1262c	-1,4	x	-1,1	0,88	*	hypothetical protein
SMU.1263	-1,5	x	-1,7	**0,01**		phosphoribosyl-ATP pyrophosphatase / phosphoribosyl-AMP cyclohydrolase
SMU.1264	-1,5	x	-1,8	**0,00**		imidazole glycerol phosphate synthase subunit HisF
SMU.1265	-1,5	x	-1,9	**0,00**		1-(5-phosphoribosyl)-5-[(5-phosphoribosylamino)methylideneamino] imidazole-4-carboxamide isomerase
SMU.1266	-1,5	x	-1,6	**0,02**		imidazole glycerol phosphate synthase subunit HisH
SMU.1267c	-1,6	x	-1,7	**0,02**		hypothetical protein
SMU.1268	-1,7	x	-1,9	**0,00**		imidazoleglycerol-phosphate dehydratase
SMU.1269	-1,6	x	-1,9	**0,00**		phosphoserine phosphatase
SMU.1270	-1,6	x	-2,0	**0,00**		bifunctional histidinal dehydrogenase/ histidinol dehydrogenase
SMU.1271	-1,7	x	-1,8	**0,00**		ATP phosphoribosyltransferase
SMU.1272	-1,6	x	-1,7	**0,01**		histidyl-tRNA synthetase
SMU.1273	-1,6	x	-1,7	**0,01**		histidinol-phosphate aminotransferase
SMU.1276c	-1,1	x	-1,2	0,23		septation ring formation regulator EzrA
SMU.1277	-1,1	x	-1,1	0,47		DNA gyrase subunit B
SMU.1278c	1,1	x	-1,2	0,39		hypothetical protein
SMU.1279c	-1,1	x	-1,0	0,92		cell shape determining protein
SMU.1280c	1,1	2,9	2,2	**0,00**		hypothetical protein
SMU.1282	-1,1	x	1,1	0,61	*	transcriptional regulator
SMU.1284c	1	3,6	1,6	**0,01**		hypothetical protein
SMU.1286c	-1,2	2,9	1,9	**0,00**		permease
SMU.1287	-1,2	x	-1,4	0,39	*	transcriptional regulator
SMU.1288	1,1	x	1,0	0,82		50S ribosomal protein L19
SMU.1289c	-1,1	x	-1,2	0,29		chloride channel permease
SMU.1290c	-1,1	x	-1,3	0,17		chloride channel permease
SMU.1291c	-1	x	-1,3	0,17		hypothetical protein
SMU.1292c	1,1	x	1,2	0,33		hypothetical protein
SMU.1293c	1,1	x	1,2	0,31		hypothetical protein
SMU.1294	-1,1	x	-1,1	0,72		flavodoxin
SMU.1295	-1	x	-1,1	0,61		adenosine deaminase

SMU.1296	-1,2	x	1,2	0,48		S-transferase
SMU.1297	-1,4	x	-1,7	**0,01**		hypothetical protein
SMU.1298	1,1	x	-1,3	0,10		50S ribosomal protein L31
SMU.1299c	1,3	x	1,9	**0,00**		acetate kinase
SMU.1300c	1,2	x	2,1	**0,00**		hypothetical protein
SMU.1301c	1,2	x	2,0	**0,00**		methyltransferase
SMU.1302	-1,1	x	1,2	0,38		surface adhesin
SMU.1303c	1,1	x	-1,1	0,50		dipeptidase
SMU.1304c	-1,1	x	-1,1	0,66		hypothetical protein
SMU.1305c	1,1	x	-1,1	0,46		hypothetical protein
SMU.1306c	1	x	-1,2	0,36		glmZ(sRNA)-inactivating NTPase
SMU.1307c	-1,1	x	-1,3	0,20		hypothetical protein
SMU.1308	-1,1	x	-1,3	0,12		translation initiation inhibitor; aldR regulator-like protein
SMU.1309c	-1,1	x	-1,3	0,16		glycerol dehydrogenase
SMU.1310	-1,2	2,2	-1,0	1,00	*	hypothetical protein
SMU.1311	-1,1	x	-1,6	**0,01**		asparaginyl-tRNA synthetase
SMU.1312	-1,1	x	-1,3	0,08		aspartate aminotransferase
SMU.1313c	-1,1	x	-1,2	0,39		bifunctional ATP-dependent DNA helicase/DNA polymerase III subunit epsilon
SMU.1314	-1,1	x	-1,5	**0,03**		hypothetical protein
SMU.1315c	-1,1	x	-1,8	**0,01**		ATP-binding protein
SMU.1316c	-1	x	-1,4	0,32	*	hypothetical protein
SMU.1317c	-2,1	x	-1,7	0,37	*	hypothetical protein
SMU.1319c	-1,1	x	-1,2	0,31		hypothetical protein
SMU.1321c	-1,1	x	-1,0	0,90		hypothetical protein
SMU.1322	1,2	4,2	1,1	0,68		acetoin reductase
SMU.1323	1,2	x	2,0	**0,00**		hydrolase
SMU.1324	-1,3	x	-1,2	0,38		cell-division protein FtsX
SMU.1325	-1	x	-1,1	0,64		ABC transporter ATP-binding protein
SMU.1326	1	x	-1,1	0,60		peptide chain release factor 2
SMU.1327c	1,1	x	1,7	**0,01**		4Fe-4S ferredoxin
SMU.1329c	1	2,4	2,6	**0,01**		transposase
SMU.1330c	-1,3	x	2,5	**0,02**	*	transposase
SMU.1331c	-1	x	3,6	**0,00**		transposase
SMU.1332c	1,1	x	5,8	**0,00**	*	transposase
SMU.1334	-1,3	x	12,0	**0,00**		phosphopantetheinyl transferase
SMU.1335c	1	x	8,3	**0,00**		enoyl-ACP reductase
SMU.1336	1	2,2	11,3	**0,00**		hypothetical protein
SMU.1337c	-1,3	2,1	17,1	**0,00**		alpha/beta hydrolase
SMU.1338c	-1,1	2,2	7,4	**0,00**		permease
SMU.1339	-1,2	x	6,6	**0,00**		bacitracin synthetase
SMU.1340	-1,5	2	4,2	**0,00**		surfactin synthetase
SMU.1341c	-1,7	x	1,7	0,22		gramicidin S synthetase
SMU.1342	-2	x	-1,5	0,35		bacitracin synthetase 1; BacA
SMU.1343c	-1,8	x	-2,6	0,12		polyketide synthase
SMU.1344c	-2	x	-2,7	**0,01**	*	malonyl CoA-ACP transacylase
SMU.1345c	-2,1	x	-3,0	0,15		peptide synthetase
SMU.1346	-2,3	x	-3,7	0,06	*	thioesterase
SMU.1347c	-1,8	x	-2,7	0,05		permease
SMU.1348c	-1,8	x	-2,5	0,11		ABC transporter ATP-binding protein
SMU.1349	1,1	x	-1,2	0,49		hypothetical protein
SMU.1351	-1,1	2	1,1	0,84		transposase fragment
SMU.1352	-1	x	-1,0	0,84	*	transposase
SMU.1353	1	x	-1,3	0,45	*	transposase
SMU.1354c	1,1	x	1,6	0,11	*	transposase fragment

SMU.1355c	1	x	1,7	0,07	*	transposase fregment
SMU.1356c	1,3	x	2,3	**0,02**	*	transposase fragment
SMU.1357	-1	x	2,0	0,66	*	transposase fragment
SMU.1358	-1,5	x	1,3	0,94	*	transposase fragment
SMU.1359	-2	x	21,1	0,79	*	hypothetical protein
SMU.1360c	-1,4	x	1,3	0,34	*	hypothetical protein
SMU.1361c	-1,2	x	1,1	0,74		TetR family transcriptional regulator
SMU.1363c	1,2	x	1,4	0,18		transposase
SMU.1365c	-1,8	x	-2,5	**0,05**		permease
SMU.1366c	-1,8	x	-2,1	0,12		ABC transporter ATP-binding protein
SMU.1367c	1	x	1,1	0,79	*	hypothetical protein
SMU.1368	1,3	3,9	11,3	**0,03**	*	hypothetical protein
SMU.1369	1,7	2,1	14,7	**0,00**	*	hypothetical protein
SMU.1370c	-1	x	1,1	0,86	*	transposase, IS150-like
SMU.1372c	1	2	1,5	0,33	*	hypothetical protein
SMU.1373c	1	2,3	2,0	0,25	*	hypothetical protein
SMU.1374	**2**	x	41,3	**0,00**	*	hypothetical protein
SMU.1375c	-1,1	x	1,0	1,00		hypothetical protein
SMU.1377c	-1,2	x	-1,3	0,16		hypothetical protein
SMU.1378	1,2	10,3	27,1	**0,00**		hypothetical protein
SMU.1379	-1,1	3,7	1,2	0,84	*	hypothetical protein
SMU.1381	1,1	2,9	2,4	**0,00**		isopropylmalate isomerase small subunit
SMU.1382	1,3	3	2,1	**0,00**		isopropylmalate isomerase large subunit
SMU.1383	1,2	2,6	2,2	**0,00**		3-isopropylmalate dehydrogenase
SMU.1384	1,2	2,6	2,4	**0,00**		2-isopropylmalate synthase
SMU.1386	1	x	1,2	0,47		uridine/cytidine kinase
SMU.1387	-1,1	x	-1,3	0,08		oxidoreductase
SMU.1388	-1,2	x	-1,3	0,15		RNA helicase
SMU.1389	1,4	x	1,6	**0,00**		hypothetical protein
SMU.1390	-1,2	x	1,0	0,90		hypothetical protein
SMU.1391c	-1,1	x	-1,3	0,12		hypothetical protein
SMU.1392c	-1,1	x	-1,4	0,06		acetyltransferase
SMU.1393c	-1,2	x	-1,5	**0,02**		hypothetical protein
SMU.1394	-1,1	x	-1,5	**0,01**		GTP-binding protein LepA
SMU.1395c	-1,1	x	-3,5	0,27	*	hypothetical protein
SMU.1396	1,4	x	1,3	0,20		glucan-binding protein GbpC
SMU.1397c	1,1	x	-1,2	0,67	*	hypothetical protein
SMU.1398	-1,2	2,4	1,1	0,69		transcriptional regulator
SMU.1399	-1,2	-2	1,9	0,54	*	hypothetical protein
SMU.1400c	**2,7**	3,1	10,7	**0,00**		hypothetical protein
SMU.1402c	-1,2	-2,3	-1,7	0,31	*	hypothetical protein
SMU.1403c	1	-2,7	-1,4	0,19	*	hypothetical protein
SMU.1404c	1	-2,1	-1,2	0,48		hypothetical protein
SMU.1405c	-1,2	-2,6	-1,2	0,49		hypothetical protein
SMU.1406c	-1,1	x	-1,0	0,99	*	hypothetical protein
SMU.1407c	-1,1	x	1,1	0,83	*	transposase, ISSmu1
SMU.1408c	-1,1	x	2,5	0,18	*	hypothetical protein
SMU.1409c	-1	x	-1,2	0,64		transcriptional regulator
SMU.1410	1,1	x	-1,2	0,45	*	reductase
SMU.1411	-1,1	x	-1,2	0,41	*	hypothetical protein
SMU.1412c	1,2	x	1,4	**0,03**		ABC transporter membrane protein subunit and ATP-binding protein
SMU.1414c	-1,2	x	-1,3	0,17		hypothetical protein
SMU.1415c	-1,1	x	-1,4	0,09		phosphatase
SMU.1416c	-1,1	x	-1,3	0,13		mutator protein MutT

SMU.1417c	-1,2	x	-1,3	0,15		oleoyl-acyl carrier protein thioesterase
SMU.1418	-1,1	x	-1,2	0,22		coproporphyrinogen III oxidase
SMU.1419	1	x	-1,2	0,50		transcriptional regulator
SMU.1420	-1,1	x	-1,3	0,18		oxidoreductase
SMU.1421	1,4	2,9	2,7	**0,00**	*	branched-chain alpha-keto acid dehydrogenase E2 subunit
SMU.1422	1,5	2,1	3,1	**0,00**	*	pyruvate dehydrogenase E1 component subunit beta
SMU.1423	1,2	2,8	2,5	**0,02**	*	pyruvate dehydrogenase, TPP-dependent E1 component alpha-subunit
SMU.1424	1,2	3,7	3,0	**0,00**	*	dihydrolipoamide dehydrogenase
SMU.1425	1	x	-1,3	0,57		Clp proteinase, ATP-binding subunit ClpB
SMU.1426c	1	x	-1,1	0,53		phosphoglucosamine mutase
SMU.1427c	-1,1	x	-1,0	0,92		hypothetical protein
SMU.1428c	-1	x	1,0	0,83		hypothetical protein
SMU.1429	-1	x	-1,1	0,55		UDP-N-acetylmuramyl tripeptide synthetase MurC
SMU.1430	-1,1	x	-1,1	0,52		cobyric acid synthase CobQ
SMU.1431c	1	x	1,1	0,49		ABC transporter ATP-binding protein
SMU.1432c	1,1	x	1,1	0,55		endoglucanase
SMU.1434c	1,1	x	1,1	0,50		glycosyltransferase
SMU.1435c	-1,2	x	-1,3	0,63	*	hypothetical protein
SMU.1436c	1	x	1,2	0,25		hypothetical protein
SMU.1437	1,1	x	1,2	0,25		UDP-N-acetylglucosamine 2-epimerase
SMU.1438c	1	x	2,1	**0,00**		Zn-dependent protease
SMU.1442c	-1,2	2,2	1,1	0,57		hypothetical protein
SMU.1443c	-1,1	x	1,2	0,26		tributyrin esterase
SMU.1444c	-1,1	x	-1,0	0,92		hypothetical protein
SMU.1445c	-1,1	x	-1,4	0,05		ABC transporter ATP-binding protein
SMU.1446c	-1,1	x	-1,4	0,07		ABC transporter permease
SMU.1447c	-1,2	x	-1,2	0,30		hypothetical protein
SMU.1449	1	x	1,2	0,23		fibronectin/fibrinogen-binding protein
SMU.1450	-1,4	x	-1,4	**0,03**		amino acid permease
SMU.1451	-1	x	1,3	0,17		alpha-acetolactate decarboxylase
SMU.1452	-1	x	1,2	0,23		acetolactate synthase
SMU.1453c	1,1	x	1,4	0,09		hypothetical protein
SMU.1454c	1	x	1,4	0,09		hypothetical protein
SMU.1455	1,2	x	1,5	0,09		mutator protein, pyrophosphohydrolase
SMU.1456c	1,1	x	1,1	0,78	*	hypothetical protein
SMU.1457	1	x	-1,3	0,14		dTDP-glucose-4,6-dehydratase
SMU.1459c	-1,1	x	-1,2	0,31		hypothetical protein
SMU.1460	-1,1	x	-1,3	0,16		dTDP-4-keto-L-rhamnose reductase
SMU.1461	-1	x	-1,2	0,29		glucose-1-phosphate thymidylyltransferase
SMU.1462c	1	x	1,1	0,62		oxidoreductase
SMU.1463c	-1,1	x	1,1	0,77		hypothetical protein
SMU.1464c	1	x	1,2	0,43		hypothetical protein
SMU.1465c	1,1	x	1,1	0,64		replication protein DnaD-like
SMU.1466	-1	x	-1,1	0,44		homoserine O-succinyltransferase
SMU.1467	1	x	-1,2	0,33		adenine phosphoribosyltransferase
SMU.1470c	-1,2	x	-1,3	0,25		hypothetical protein
SMU.1471c	-1,1	x	-1,1	0,73	*	hypothetical protein
SMU.1472	-1	3	-1,3	0,17		single-strand DNA-specific exonuclease RecJ
SMU.1473c	-1,2	2,8	-1,2	0,23		oxidoreductase
SMU.1474c	-1,1	2,8	-1,3	0,13		ribonuclease Z
SMU.1475c	-1,1	3,1	-1,1	0,59		hypothetical protein
SMU.1476c	-1,1	2,1	-1,4	**0,04**		GTP-binding protein
SMU.1477	-1	x	-1,1	0,72		tRNA delta(2)-isopentenylpyrophosphate transferase
SMU.1479	1,1	x	1,1	0,55		hypothetical protein

SMU.1480	1,1	x	1,5	**0,04**		hypothetical protein
SMU.1482c	-1,3	x	-1,5	**0,02**		hypothetical protein
SMU.1483c	-1,1	x	-1,5	**0,03**		hypothetical protein
SMU.1484c	-1,1	x	-1,8	**0,00**		hypothetical protein
SMU.1485c	-1,3	x	-1,8	**0,00**		endonuclease
SMU.1486c	-1,1	x	-1,2	0,50	*	hypothetical protein
SMU.1487	1,6	x	2,2	**0,00**		hypothetical protein
SMU.1488c	1,2	x	-1,1	0,86	*	hypothetical protein
SMU.1489	1,1	x	1,0	0,99	*	hypothetical protein
SMU.1490	1,1	x	1,3	0,75		6-phospho-beta-galactosidase
SMU.1491	1	x	1,1	0,90		PTS system lactose-specific transporter subunit IIBC
SMU.1492	1,2	x	1,3	0,80	*	PTS system lactose-specific transporter subunit IIA
SMU.1493	1	x	1,1	0,94	*	tagatose 1,6-diphosphate aldolase
SMU.1494	1,1	x	1,2	0,88	*	tagatose-6-phosphate kinase
SMU.1495	-1	2	-1,4	0,61	*	galactose-6-phosphate isomerase subunit LacB
SMU.1496	-1,1	x	2,6	0,37	*	galactose-6-phosphate isomerase subunit LacA
SMU.1498	-1	x	1,4	0,23		lactose repressor
SMU.1499	1,1	x	1,3	0,19		exonuclease RexA
SMU.1500	1,1	2	1,5	**0,02**		exonuclease RexB
SMU.1502c	-1	x	1,2	0,36		hypothetical protein
SMU.1504c	-1,1	2,1	1,7	0,12	*	hypothetical protein
SMU.1505c	-1,4	x	1,3	0,61	*	hypothetical protein
SMU.1506c	-1	x	1,5	0,10		hypothetical protein
SMU.1507c	1,1	x	2,3	0,10	*	hypothetical protein
SMU.1508c	-1,1	x	1,6	0,07		coenzyme PQQ synthesis protein
SMU.1509	-1	x	2,0	**0,02**	*	transcriptional regulator
SMU.1510	-1,2	x	-1,9	**0,00**		phenylalanyl-tRNA synthetase subunit beta
SMU.1511c	-1,4	x	-1,7	**0,00**		acetyltransferase
SMU.1512	-1,1	x	-1,5	**0,04**		phenylalanyl-tRNA synthetase subunit alpha
SMU.1513	-1	x	1,0	0,96		chromosome segregation ATPase
SMU.1514	-1,1	x	-1,4	0,05		ribonuclease III
SMU.1515	1	2,4	-1,0	0,83		hypothetical protein
SMU.1516	-1,1	2	1,1	0,61		histidine kinase CovS
SMU.1517	-1,1	2	-1,1	0,69		response regulator CovR
SMU.1519	1	x	-1,1	0,80		amino acid ABC transporter ATP-binding protein
SMU.1520	-1,1	x	-1,0	0,98		ABC transporter glutamine binding protein
SMU.1521	-1,1	x	-1,0	0,98		amino acid ABC transporter permease
SMU.1522	-1,1	x	-1,1	0,86		amino acid ABC transporter integral membrane protein
SMU.1523	1	x	1,1	0,45		membrane nuclease EndA
SMU.1524c	-1,2	x	1,2	0,46		hypothetical protein
SMU.1525	1	x	1,0	0,87		UDP-N-acetylglucosamine 1-carboxyvinyltransferase
SMU.1526c	1,1	x	-1,3	0,73	*	hypothetical protein
SMU.1527	1,1	x	-1,2	0,32		ATP synthase F0F1 subunit epsilon
SMU.1528	-1	x	-1,3	0,07		ATP synthase F0F1 subunit beta
SMU.1529	-1,1	x	-1,2	0,33		ATP synthase F0F1 subunit gamma
SMU.1530	1	x	-1,2	0,23		ATP synthase F0F1 subunit alpha
SMU.1531	-1,2	x	-1,0	0,88		ATP synthase F0F1 subunit delta
SMU.1532	1	x	-1,1	0,63		ATP synthase F0F1 subunit B
SMU.1533	-1,1	x	-1,0	0,92		ATP synthase F0F1 subunit A
SMU.1534	-1,1	x	-1,2	0,53		ATP synthase F0F1 subunit C
SMU.1535	1	x	-1,6	**0,00**		glycogen phosphorylase
SMU.1536	1	x	-1,6	**0,01**		glycogen synthase
SMU.1537	-1,1	x	-1,8	**0,00**		glycogen biosynthesis protein GlgD
SMU.1538	1	x	-1,8	**0,00**		glucose-1-phosphate adenylyltransferase

SMU.1539	1	x	-1,5	**0,01**		glycogen branching protein
SMU.1541	-1,1	x	-1,2	0,21		pullulanase
SMU.1542c	-1	x	-1,2	0,34		lipid kinase
SMU.1543	-1	x	-1,2	0,30		NAD-dependent DNA ligase LigA
SMU.1545c	-1,3	x	-1,2	0,42		hypothetical protein
SMU.1546	-1,2	x	-1,8	0,16	*	hypothetical protein
SMU.1547c	1,1	x	-1,0	0,77		response regulator
SMU.1548c	1,1	x	-1,1	0,73		histidine kinase
SMU.1550c	-1,1	x	1,5	0,15	*	hypothetical protein
SMU.1551c	-1	2	1,2	0,35		ABC transporter ATP-binding protein
SMU.1552c	1,2	x	1,2	0,34		hypothetical protein
SMU.1553c	-1,2	2,8	1,4	0,43	*	hypothetical protein
SMU.1554c	-1,1	x	1,5	0,14	*	hypothetical protein
SMU.1555c	-1,2	x	-1,1	0,60		hypothetical protein
SMU.1556	1,1	x	-1,2	0,27		methionine aminopeptidase
SMU.1557c	1	x	1,1	0,49		hypothetical protein
SMU.1558c	1,2	x	1,2	0,46		acetyltransferase
SMU.1560	1	x	1,2	0,83	*	hypothetical protein
SMU.1561	-1	x	1,0	0,84		potassium uptake system protein TrkB
SMU.1562	1	x	1,0	0,82		potassium uptake protein TrkA
SMU.1563	1,1	x	1,0	0,81		cation-transporting P-type ATPase PacL
SMU.1564	1	x	-1,1	0,63		glycogen phosphorylase
SMU.1565	1,1	x	-1,1	0,79		4-alpha-glucanotransferase
SMU.1566	-1,2	x	-1,1	0,60		maltose operon transcriptional repressor
SMU.1568	-1,1	x	-1,3	0,32		maltose ABC transporter substrate-binding protein
SMU.1569	1	-2,7	-1,6	0,17		maltose ABC transporter permease
SMU.1570	-1,1	x	-1,3	0,44		maltose ABC transporter permease
SMU.1571	1	x	-1,4	0,28		MsmK-like ABC transporter ATP-binding protein
SMU.1572	1,1	x	-1,1	0,69		UDP-N-acetylglucosamine 1-carboxyvinyltransferase
SMU.1573	-1,1	x	-1,4	**0,05**		S-adenosylmethionine synthetase
SMU.1574c	-1,4	x	1,2	0,37		hypothetical protein
SMU.1575c	-1,4	x	-2,4	0,59	*	hypothetical protein
SMU.1576c	-1,3	x	1,1	0,81	*	hypothetical protein
SMU.1577c	-1,2	x	-1,0	1,00	*	hypothetical protein
SMU.1578	1,2	x	1,3	0,23		bifunctional biotin--[acetyl-CoA-carboxylase] synthetase/biotin operon repressor
SMU.1579	-1,2	x	1,3	0,28	*	hypothetical protein
SMU.1581	1,1	x	-1,3	0,17		DNA polymerase III subunits gamma and tau
SMU.1582c	-1	x	-1,3	0,18		hypothetical protein
SMU.1584c	-1,1	x	1,3	0,40	*	hypothetical protein
SMU.1585c	-1	x	1,1	0,69		transcriptional regulator
SMU.1586	-1,1	x	-1,6	**0,01**		threonyl-tRNA synthetase
SMU.1587c	-1,1	x	-1,7	**0,01**		hypothetical protein
SMU.1588c	-1	x	-1,0	0,98		hexosyltransferase
SMU.1589c	-1	x	-1,0	0,93		hexosyltransferase
SMU.1590	1,2	x	1,2	0,38		alpha-amylase
SMU.1591	1,3	x	1,1	0,41		catabolite control protein CcpA
SMU.1592	-1,2	x	-1,6	0,05		dipeptidase PepQ
SMU.1593c	1,1	x	1,2	0,22		CDP-diglyceride synthetase
SMU.1595	-1,2	x	1,7	**0,01**		carbonic anhydrase
SMU.1596	-1	x	-1,2	0,64	*	cellobiose phosphotransferase system IIC component
SMU.1597c	-1,2	x	-118,7	0,18	*	hypothetical protein
SMU.1598	1,1	x	-2,1	0,38	*	cellobiose phosphotransferase system IIA component
SMU.1599	1	x	-1,4	0,51	*	transcriptional regulator
SMU.1600	1	-2,2	-2,9	0,50	*	PTS system cellobiose transporter subunit IIB

SMU.1601	1	x	1,0	0,99	*	6-phospho-beta-glucosidase
SMU.1602	1,2	x	1,1	0,58		NAD(P)H-flavin oxidoreductase
SMU.1603	1,2	x	1,2	0,31		lactoylglutathione lyase
SMU.1604c	-1,5	x	1,9	0,24	*	hypothetical protein
SMU.1605	1	x	-1,5	0,27	*	MDR permease
SMU.1606	-1	x	1,1	0,69		SsrA-binding protein
SMU.1607	-1,1	x	1,0	0,82		exoribonuclease R
SMU.1609c	-1,1	x	-1,0	0,87		preprotein translocase subunit SecG
SMU.1610	-1,1	3,6	1,3	0,44	*	50S ribosomal protein L33
SMU.1611c	-1	x	1,1	0,49		permease
SMU.1612c	1	x	1,1	0,73		hypothetical protein
SMU.1613c	1,1	x	1,1	0,68		dephospho-CoA kinase
SMU.1614	1,1	x	1,1	0,67		formamidopyrimidine/5-formyluracil/ 5-hydroxymethyluracil DNA glycosylase
SMU.1615c	1,1	x	-1,0	0,97		hypothetical protein
SMU.1616c	-1	x	1,0	0,83		hypothetical protein
SMU.1617	-1,1	x	1,0	0,88		GTPase Era
SMU.1618	-1	x	-1,0	0,92		diacylglycerol kinase
SMU.1619c	1,1	x	1,1	0,72		metal-binding heat shock protein
SMU.1620	1,1	x	-1,0	0,99		phosphate starvation-induced protein PhoH
SMU.1621c	1,2	3,2	1,1	0,68		hypothetical protein
SMU.1622	1,1	x	1,2	0,57		methionine sulfoxide reductase A
SMU.1623c	-1,2	x	-1,1	0,57		hypothetical protein
SMU.1624	-1,1	x	-1,1	0,42		ribosome recycling factor
SMU.1625	-1	x	-1,2	0,19		uridylate kinase
SMU.1626	-1	x	-1,0	0,88		50S ribosomal protein L1
SMU.1627	1	x	-1,1	0,66		50S ribosomal protein L11
SMU.1628	-1,2	x	-1,1	0,80	*	hypothetical protein
SMU.1629c	-1	x	-1,1	0,51		cell division DNA segregation ATPase
SMU.1631	-1,4	x	-1,0	0,96		peptidyl-prolyl cis-trans isomerase
SMU.1632	-1	x	-1,1	0,52		5'-methylthioadenosine/S-adenosylhomocysteine nucleosidase
SMU.1633c	-1	x	-1,0	0,96		hypothetical protein
SMU.1634c	-1,1	x	-1,1	0,61		hypothetical protein
SMU.1635	-1	x	1,0	0,96		UDP-N-acetylglucosamine pyrophosphorylase
SMU.1636c	1,1	x	1,0	0,97		hypothetical protein
SMU.1637c	1,1	x	-1,0	0,85		hypothetical protein
SMU.1638c	-1,2	x	1,0	0,87		hypothetical protein
SMU.1639	1,1	x	1,0	0,86		methionyl-tRNA synthetase
SMU.1641c	-1,1	x	-1,7	**0,01**		hypothetical protein
SMU.1642c	1,1	2,1	1,7	**0,01**		hypothetical protein
SMU.1643c	1,1	x	1,8	**0,02**		hypothetical protein
SMU.1644c	-1,4	x	1,0	0,84		hypothetical protein
SMU.1645	-1,2	x	-1,1	0,76		tellurite resistance protein TehB
SMU.1646c	-1,2	x	1,1	0,72		hemolysis inducing protein
SMU.1647c	1,1	x	1,4	0,12		transcriptional regulator
SMU.1648c	-1	x	1,5	0,37	*	hypothetical protein
SMU.1649	-1,1	x	-1,2	0,29		exodeoxyribonuclease III
SMU.1650	-1	x	3,4	**0,00**		endonuclease III (DNA repair)
SMU.1651	1,7	x	1,6	**0,01**		arsenate reductase
SMU.1652	1,2	x	1,0	0,82		methylated DNA-protein cysteine methyltransferase
SMU.1653	-1,1	x	-1,3	0,08		D-3-phosphoglycerate dehydrogenase
SMU.1654c	1	x	-1,4	**0,04**		acetyltransferase
SMU.1655c	1	x	-1,4	0,13		hypothetical protein
SMU.1656	1	x	-1,3	0,14		3-phosphoserine/phosphohydroxythreonine aminotransferase
SMU.1657c	-1	-2,5	-2,3	0,23		nitrogen regulatory protein PII

SMU.1658	-1,1	x	-2,5	0,23		ammonium transporter
SMU.1659c	-1,1	x	-1,1	0,77		hypothetical protein
SMU.1660c	1,1	x	1,1	0,74		DNA replication intiation control protein YabA
SMU.1661c	-1,1	x	1,0	0,99		signal peptidase II
SMU.1662	-1,1	x	1,0	0,83		DNA polymerase III subunit delta'
SMU.1663	1,1	x	1,1	0,82		thymidylate kinase
SMU.1664c	-1	x	1,1	0,66		acetoin utilization protein, acetoin dehydrogenase
SMU.1665	-1	x	-1,1	0,53		branched chain amino acid ABC transporter ATP-binding protein
SMU.1666	1	x	-1,1	0,55		branched chain amino acid ABC transporter ATP-binding protein
SMU.1667	-1	x	-1,0	0,78		branched chain amino acid ABC transporter permease
SMU.1668	1	x	-1,1	0,63		branched chain amino acid ABC transporter permease
SMU.1669	-1,1	x	-1,3	0,10		branched chain amino acid ABC transporter substrate-binding protein
SMU.1670c	-1,3	x	-1,2	0,55	*	hypothetical protein
SMU.1671c	1	x	-1,0	0,91		hypothetical protein
SMU.1672	-1	x	1,1	0,79		ATP-dependent Clp protease proteolytic subunit
SMU.1673	-1,1	x	-1,4	**0,04**		uracil phosphoribosyltransferase
SMU.1674	1,1	x	1,2	0,26		aminotransferase
SMU.1675	1,1	x	1,3	0,13		cystathionine gamma-synthase
SMU.1676c	-1	x	-1,1	0,77		hypothetical protein
SMU.1677	1,3	x	1,8	**0,00**		UDP-N-acetylmuramoylalanyl-D-glutamate--L-lysine ligase
SMU.1678	1,3	2,4	1,9	**0,00**		acyl-CoA thioesterase
SMU.1679c	1,1	x	1,2	0,20		hypothetical protein
SMU.1680c	1,1	x	1,3	0,18		hypothetical protein
SMU.1681c	1,2	x	1,2	0,20		hypothetical protein
SMU.1682c	-1,1	x	-1,1	0,88	*	intracellular protease
SMU.1683c	-1,1	x	-1,1	0,61		hypothetical protein
SMU.1685c	-1,1	x	-1,2	0,49		hypothetical protein
SMU.1687	-1	x	-1,0	0,91		manganese-dependent inorganic pyrophosphatase
SMU.1688	-1,1	x	1,0	0,91		extramembranal protein, DltD protein
SMU.1689	-1,1	x	1,0	0,88		D-alanine--poly(phosphoribitol) ligase subunit 2
SMU.1690	-1,2	x	-1,1	0,62		hypothetical protein
SMU.1691	-1	x	-1,2	0,40		D-alanine--poly(phosphoribitol) ligase subunit 1
SMU.1692	-1,1	x	-1,1	0,76		pyruvate-formate lyase activating enzyme
SMU.1693	-1	x	1,0	0,87		hemolysin
SMU.1694c	-1,2	x	-1,9	0,16	*	permease
SMU.1695	-1	x	-1,3	0,16		molybdenum ABC transporter ATP-binding protein
SMU.1697c	1,1	x	1,1	0,75		hypothetical protein
SMU.1699c	1,2	x	-1,0	0,92		hypothetical protein
SMU.1700c	-1,1	x	-1,1	0,68	*	LrgB family protein
SMU.1701c	-1,2	2	1,3	0,50	*	hypothetical protein
SMU.1702c	-1,2	x	-1,4	0,11		phosphatase
SMU.1703c	-1,1	x	-1,4	0,09		hypothetical protein
SMU.1704	1,4	x	1,0	0,95	*	hypothetical protein
SMU.1705	1,2	x	1,5	0,23	*	hypothetical protein
SMU.1706	1,1	x	1,1	0,67		hypothetical protein
SMU.1707c	-1,1	x	1,1	0,55		rRNA methylase
SMU.1708	-1	x	-1,3	0,10		potassium transporter peripheral membrane protein
SMU.1709	-1	x	-1,3	0,17		potassium uptake protein TrkH
SMU.1710c	-1,1	x	1,3	0,25		hypothetical protein
SMU.1711	1	x	1,1	0,50		pseudouridylate synthase
SMU.1712c	-1,1	x	1,3	0,15		segregation and condensation protein B
SMU.1713c	-1,1	2	1,2	0,25		segregation and condensation protein A
SMU.1714c	-1,3	2	1,0	0,80		site-specific tyrosine recombinase XerD
SMU.1715c	-1,1	x	1,1	0,43		hypothetical protein

SMU.1716c	-1	x	1,0	0,81		hypothetical protein
SMU.1717c	1	x	1,0	1,00		deoxyribonucleotide triphosphate pyrophosphatase/hypothetical protein
SMU.1718	1	x	1,2	0,37		glutamate racemase
SMU.1719c	-1,1	x	1,1	0,60		hypothetical protein
SMU.1721c	1	x	1,2	0,35		diaminopimelate decarboxylase
SMU.1722c	-1,1	x	-1,0	0,97		hypothetical protein
SMU.1723c	1,2	x	-1,0	0,94		hypothetical protein
SMU.1724c	1,1	x	1,0	0,82		rRNA methylase
SMU.1725	-1	x	1,3	0,55	*	acylphosphatase
SMU.1727	1,1	x	1,4	**0,05**		OxaA-like protein precursor
SMU.1728	-1,2	x	1,1	0,53		transcription elongation factor GreA
SMU.1729c	-1,2	x	1,0	0,87		aminodeoxychorismate lyase
SMU.1730c	-1,3	x	-1,1	0,53		acetyltransferase
SMU.1731	-1,2	x	-1,2	0,32		UDP-N-acetyl muramate-alanine ligase
SMU.1732c	-1,3	x	-1,0	0,93		hypothetical protein
SMU.1733c	-1,1	2,1	1,1	0,72		SNF helicase
SMU.1734	1,1	x	-1,1	0,62		acetyl-CoA carboxylase subunit alpha
SMU.1735	-1	x	-1,1	0,55		acetyl-CoA carboxylase subunit beta
SMU.1736	-1	x	-1,1	0,74		acetyl-CoA carboxylase biotin carboxylase subunit
SMU.1737	-1,1	x	-1,2	0,29		(3R)-hydroxymyristoyl-ACP dehydratase
SMU.1738	1	x	-1,1	0,55		biotin carboxyl carrier protein of acetyl-CoA carboxylase
SMU.1739	-1	x	-1,1	0,54		3-oxoacyl-ACP synthase
SMU.1740	-1	x	-1,0	0,81		3-ketoacyl-ACP reductase
SMU.1741	-1,1	x	-1,1	0,72		malonyl CoA-ACP transacylase
SMU.1742c	-1,1	x	-1,0	0,83		trans-2-enoyl-ACP reductase
SMU.1743	-1,1	x	-1,4	0,17		acyl carrier protein
SMU.1744	-1	x	-1,1	0,53		3-oxoacyl-ACP synthase
SMU.1745c	-1,1	x	-1,1	0,43		transcriptional regulator
SMU.1746c	1,1	x	1,1	0,48		enoyl-CoA hydratase
SMU.1747c	-1	x	1,3	0,28		phosphatase
SMU.1748	-1,1	x	1,1	0,69		aspartate kinase
SMU.1750c	-1,2	x	-1,3	0,24		hypothetical protein
SMU.1752c	1,1	-2,5	-1,2	0,59		hypothetical protein
SMU.1753c	1,2	x	-1,7	**0,02**		hypothetical protein
SMU.1754c	1	x	-1,6	**0,04**		hypothetical protein
SMU.1755c	1,1	x	-1,6	0,14		hypothetical protein
SMU.1757c	-1	x	-1,7	0,09		hypothetical protein
SMU.1758c	1,1	x	-1,7	0,05		hypothetical protein
SMU.1760c	1,1	x	-1,5	0,24		hypothetical protein
SMU.1761c	-1,1	x	-1,5	0,07		hypothetical protein
SMU.1762c	-1,2	x	-1,4	0,20		hypothetical protein
SMU.1763c	1,1	-2	-1,7	0,14		hypothetical protein
SMU.1764c	-1	-2,3	-1,9	**0,04**		hypothetical protein
SMU.1765c	-1,1	x	-1,0	0,86		hypothetical protein
SMU.1766c	-1,3	x	-1,1	0,62		hypothetical protein
SMU.1767c	-1,2	x	-1,2	0,30		hypothetical protein
SMU.1768c	-1,1	x	-1,2	0,27		hypothetical protein
SMU.1770	-1,2	x	-1,3	0,08		valyl-tRNA synthetase
SMU.1771c	-1,4	x	-1,5	0,51	*	hypothetical protein
SMU.1772c	-1,4	x	-1,5	0,51	*	hypothetical protein
SMU.1773c	-1,3	x	-1,3	0,45	*	hypothetical protein
SMU.1774c	1	x	1,0	0,96		hypothetical protein
SMU.1775c	-1	x	1,2	0,40		hypothetical protein
SMU.1776c	-1,2	x	1,1	0,85	*	hypothetical protein

SMU.1777	-1,1	x	1,0	0,96		flavoprotein NrdI
SMU.1779c	-1,1	x	1,7	**0,00**		RNA methyltransferase
SMU.1780	-1,2	x	-1,2	0,57	*	recombination regulator RecX
SMU.1781	-1,2	x	1,0	0,79		hypothetical protein
SMU.1782	-1,2	x	1,1	0,76		hypothetical protein
SMU.1783	-1,1	x	-1,1	0,40		prolyl-tRNA synthetase
SMU.1784c	-1,1	x	-1,2	0,38		Eep protein-like protein
SMU.1785	-1,1	x	-1,1	0,67		phosphatidate cytidylyltransferase synthase
SMU.1786	-1	x	-1,0	0,75		undecaprenyl pyrophosphate synthase
SMU.1787c	-1	x	1,1	0,43		preprotein translocase subunit YajC
SMU.1788c	-1,1	x	-1,2	0,60		bacterocin transport accessory protein, Bta
SMU.1789c	-1	x	-1,4	0,08		hypothetical protein
SMU.1790c	1,2	x	1,1	0,68		transcriptional regulator
SMU.1791c	1,1	x	-1,0	0,92		hypothetical protein
SMU.1792c	1,2	x	-1,2	0,34		hypothetical protein
SMU.1794c	1	x	-1,1	0,55		hypothetical protein
SMU.1795c	1	x	-1,1	0,75		hypothetical protein
SMU.1797c	1,1	x	-1,1	0,72		hypothetical protein
SMU.1798c	1,1	x	-1,1	0,55		hypothetical protein
SMU.1799	-1,1	x	-1,1	0,72		nicotinic acid mononucleotide adenylyltransferase
SMU.1800c	-1	x	-1,1	0,44		hypothetical protein
SMU.1801c	1	x	-1,2	0,27		GTPase YqeH
SMU.1802c	1,1	x	-1,1	0,61		hypothetical protein
SMU.1803c	-1,2	-2,3	-1,4	0,21		hypothetical protein
SMU.1804c	-1,5	-3,8	-2,2	0,39	*	hypothetical protein
SMU.1805	1,1	x	1,6	0,32	*	transcriptional regulator
SMU.1806	-1	x	1,3	0,58	*	glycosyltransferase
SMU.1807c	-1	x	1,1	0,82		permease
SMU.1808c	-1,1	x	-1,1	0,84	*	integrase fragment
SMU.1809	1,1	x	-1,3	0,22		bacteroiocin operon protein ScnG-like protein
SMU.1810	1	x	-1,2	0,47	*	bacteriocin operon component, ScnE-like protein
SMU.1811	1	x	-1,3	0,28		bacteriocin component ScnF-like protein
SMU.1812	-1	x	4,3	0,32	*	transposase, ISSmu2
SMU.1813	1,3	x	2,3	0,63	*	transposase fragment
SMU.1814	-1,2	x	-1,5	**0,04**		histidine kinase, ScnK-like protein
SMU.1815	-1,2	x	-1,1	0,58		response regulator; ScnR-like protein
SMU.1816c	1	x	-1,5	0,27	*	maturase-like protein
SMU.1817c	-1,2	x	-1,3	0,70	*	maturase-like protein
SMU.1818c	-1,1	x	-1,2	0,51	*	hypothetical protein
SMU.1819	-1,2	x	-1,2	0,24		aspartyl/glutamyl-tRNA amidotransferase subunit B
SMU.1820c	-1,1	x	-1,2	0,32		aspartyl/glutamyl-tRNA amidotransferase subunit A
SMU.1821c	-1,2	x	-1,2	0,50		aspartyl/glutamyl-tRNA amidotransferase subunit C
SMU.1822	-1,1	x	-1,1	0,62		aspartyl-tRNA synthetase
SMU.1823	1,1	x	1,0	0,92		pyrazinamidase/nicotinamidase
SMU.1824c	-1	x	1,1	0,53		transcriptional repressor CodY
SMU.1826	1	x	-1,0	0,84		aminotransferase
SMU.1827	-1,1	x	-1,5	0,29	*	biotin biosynthesis protein
SMU.1828	1,1	x	-1,1	0,56		hypothetical protein
SMU.1830c	1,1	x	1,2	0,48		hypothetical protein
SMU.1831	1	x	-1,0	0,73		L-asparaginase
SMU.1832	-1,4	x	-1,3	0,33		hypothetical protein
SMU.1833	1,3	x	1,5	**0,04**		ATP-dependent DNA helicase, RecG
SMU.1834	1,2	x	1,2	0,43		alanine racemase
SMU.1835	1	x	1,1	0,77		4'-phosphopantetheinyl transferase

SMU.1836	-1	x	1,0	0,96		phospho-2-dehydro-3-deoxyheptonate aldolase
SMU.1837	1,1	x	1,1	0,72		phospho-2-dehydro-3-deoxyheptonate aldolase
SMU.1838	1,1	x	-1,0	0,91		preprotein translocase subunit SecA
SMU.1839	-1	x	-1,2	0,35		mannose-6-phosphate isomerase
SMU.1840	-1,1	x	-1,0	0,96		fructokinase
SMU.1841	1,2	x	1,1	0,64		PTS system sucrose-specific transporter subunit IIABC
SMU.1843	1	x	-1,4	**0,04**		sucrose-6-phosphate hydrolase
SMU.1844	-1,1	x	-1,6	**0,01**		sucrose operon repressor
SMU.1845	-1,1	x	-1,1	0,71		transcription antitermination protein NusB
SMU.1846c	-1	x	-1,1	0,71		hypothetical protein
SMU.1847	-1,2	x	1,1	0,68		elongation factor P
SMU.1848	1,1	x	-1,1	0,85	*	hypothetical protein
SMU.1849	1,2	x	1,9	**0,00**		deoxycytidylate deaminase
SMU.1850	1,2	x	1,7	**0,00**		aminopeptidase
SMU.1851	1,2	x	1,7	**0,00**		excinuclease ABC subunit A
SMU.1852	-1,1	x	-1,2	0,26		magnesium/cobalt transport protein
SMU.1853	-1,1	x	-1,2	0,23		hypothetical protein
SMU.1854	-1,1	x	-1,1	0,89		hypothetical protein
SMU.1855	1	2,1	1,1	0,75		hypothetical protein
SMU.1856c	1	2,5	1,6	0,13		hypothetical protein
SMU.1858	-1	x	-1,2	0,36		30S ribosomal protein S18
SMU.1859	1,2	x	-1,1	0,70		single-stranded DNA-binding protein
SMU.1860	1,1	x	1,1	0,72		30S ribosomal protein S6
SMU.1861c	-1,9	x	-3,4	0,15	*	hypothetical protein
SMU.1862	1,1	2,4	9,0	**0,00**	*	hypothetical protein
SMU.1865	-1	x	-1,2	0,31		A/G-specific adenine glycosylase
SMU.1867c	-1,1	x	-1,2	0,25		alcohol dehydrogenase
SMU.1869	-1,1	x	1,1	0,58		thioredoxin
SMU.1870	-1,1	x	-1,2	0,37		DNA mismatch repair protein MutS2
SMU.1871c	-1,4	x	-1,7	0,07		hypothetical protein
SMU.1872c	-1,4	x	-1,7	**0,00**		hypothetical protein
SMU.1873	-1,1	x	-1,1	0,54		ribonuclease HIII
SMU.1874	-1,1	x	-1,3	0,16		signal peptidase I
SMU.1875	-1,1	x	1,0	0,76		exodeoxyribonuclease V
SMU.1876	-1,4	x	-1,5	0,09		hypothetical protein
SMU.1877	-1	-2	-1,9	**0,00**		PTS system mannose-specific transporter subunit IIAB
SMU.1878	-1,2	x	-1,9	**0,00**		PTS system mannose-specific transporter subunit IIC
SMU.1879	-1,1	x	-2,0	**0,00**		PTS system mannose-specific transporter subunit IID
SMU.1881c	-1,3	x	-2,0	**0,01**		ABC transporter ATP-binding protein
SMU.1882c	-1,5	x	-1,9	0,16		hypothetical protein
SMU.1883	-1,1	x	1,2	0,45		hypothetical protein
SMU.1884c	-1,1	2,8	1,5	0,33	*	hypothetical protein
SMU.1886	-1,3	x	-1,8	**0,01**		seryl-tRNA synthetase
SMU.1888	1,1	x	1,1	0,98	*	transposase fragment
SMU.1889c	-1	x	-1,1	0,84	*	hypothetical protein
SMU.1891c	1,1	2,3	1,0	0,93	*	hypothetical protein
SMU.1892c	1,1	x	-1,3	0,72	*	hypothetical protein
SMU.1893c	-1,2	x	-1,3	0,48	*	transposase, ISSmu1
SMU.1894c	-1,1	x	1,3	0,72	*	hypothetical protein
SMU.1895c	-1,7	-5,2	-4,3	**0,03**		hypothetical protein
SMU.1896c	-1,4	-4,3	-4,4	**0,03**		hypothetical protein
SMU.1897	1,7	x	-1,3	0,36	*	ABC transporter ATP-binding protein
SMU.1898	1,1	x	-1,0	0,92	*	ABC transporter ATP-binding protein/permease
SMU.1899	1,2	4,4	2,9	0,39	*	ABC transporter ATP-binding protein/permease (fragment)

SMU.1900	1,1	x	-1,6	0,26	*	hypothetical protein
SMU.1902c	**2,5**	6,5	5,2	**0,01**		hypothetical protein
SMU.1903c	**16**	6	22,3	**0,00**		hypothetical protein
SMU.1904c	**49,7**	5,5	35,2	**0,00**		hypothetical protein
SMU.1905c	**47,8**	5	52,1	**0,00**		bacteriocin secretion protein
SMU.1906c	**34**	6,5	37,5	**0,00**		hypothetical protein
SMU.1907	-1,1	5,6	31,9	0,67	*	hypothetical protein
SMU.1908c	**57,1**	4,2	41,7	**0,00**		hypothetical protein
SMU.1909c	**55,1**	5,1	42,3	**0,00**		hypothetical protein
SMU.1910c	**39,2**	4,6	42,3	**0,00**		hypothetical protein
SMU.1912c	**29,9**	4	51,1	**0,00**		hypothetical protein
SMU.1913c	**29,3**	3	40,1	**0,00**		immunity protein, BLpL-like
SMU.1914c	**17,4**	3,1	34,7	**0,00**		hypothetical protein
SMU.1915	1	2,2	-1,7	0,19	*	competence stimulating peptide
SMU.1916	**6,3**	8,9	43,6	**0,00**		histidine kinase of the competence regulon, ComD
SMU.1917	**10**	7,8	58,4	**0,00**		response regulator of the competence regulon ComE
SMU.1918	1,2	2,4	1,8	**0,00**		membrane-associated protein DedA
SMU.1919	1,3	2,3	2,1	**0,00**		hypothetical protein
SMU.1920	1,4	2,2	1,8	**0,00**		GTP-binding protein Der
SMU.1921	-1,1	x	1,1	0,43		primosomal protein DnaI
SMU.1922	-1,2	x	1,1	0,39		chromosome replication protein
SMU.1923c	-1,1	2	1,1	0,48		transcriptional regulator NrdR
SMU.1924	-1,1	x	-1,0	0,78		response regulator GcrR for glucan-binding protein C
SMU.1925c	-1,1	x	1,4	0,17		hypothetical protein
SMU.1926	-1	2	1,7	**0,02**		transcriptional regulator
SMU.1927	1,1	2,8	2,5	**0,00**		ABC transporter ATP-binding protein
SMU.1928	1	2,4	2,6	**0,00**		ABC transporter permease
SMU.1929	1,1	x	1,0	0,82		heat shock protein HtpX
SMU.1930	-1,1	x	-1,1	0,49		cytoplasmic membrane protein
SMU.1931	-1,1	x	-1,4	0,10		16S rRNA methyltransferase GidB
SMU.1933c	1,1	x	1,2	0,61		cobalt permease
SMU.1934c	1,2	x	1,1	0,82		cobalt ABC transporter ATP-binding protein
SMU.1935c	1,1	x	-1,0	0,94		hypothetical protein
SMU.1936c	1,2	x	1,0	0,93		hypothetical protein
SMU.1937	1,2	x	-1,0	0,80		carbon-nitrogen hydrolase
SMU.1938c	-1	x	-1,0	0,80		ABC transporter permease
SMU.1939c	1,1	x	1,0	0,95		ABC transporter ATP-binding protein
SMU.1940c	1	x	1,1	0,84		peptidase, AtmC; ArgE/DapE/Acy1 family protein
SMU.1941	-1,3	x	1,1	0,71		membrane lipoprotein
SMU.1942c	-1,1	x	1,1	0,75		amino acid binding protein
SMU.1943	-1,1	x	-1,3	0,11		leucyl-tRNA synthetase
SMU.1945	-1,2	x	-1,4	0,22		hypothetical protein
SMU.1946	-1,2	x	-1,6	**0,03**		hypothetical protein
SMU.1947	-1,1	x	1,1	0,51		transcription antitermination protein NusG
SMU.1948	1,2	x	-1,8	**0,01**		preprotein translocase subunit SecE
SMU.1949	1	x	1,1	0,74		membrane carboxypeptidase, penicillin-binding protein 2a
SMU.1950	-1	x	-1,4	0,11		pseudouridylate synthase
SMU.1951c	1,1	x	1,6	**0,04**		hypothetical protein
SMU.1954	1,3	x	1,5	**0,04**		molecular chaperone GroEL
SMU.1955	1,2	x	1,5	0,07		co-chaperonin GroES
SMU.1956c	-1,1	2	9,6	**0,00**		hypothetical protein
SMU.1957	-1,1	3,3	8,8	**0,00**		PTS system mannose-specific transporter subunit IID
SMU.1958c	1,1	2,8	10,2	**0,00**		PTS system mannose-specific transporter subunit IIC
SMU.1960c	-1,1	3	9,9	**0,00**		PTS system mannose-specific transporter subunit IIB

SMU.1961c	1,1	3,4	10,7	**0,00**		PTS system sugar-specific transporter subunit IIA
SMU.1963c	1,9	3,5	20,0	**0,00**		sugar-binding periplasmic protein
SMU.1964c	**2**	3,9	27,1	**0,00**		response regulator
SMU.1965c	**2,4**	4,1	32,1	**0,00**		histidine kinase
SMU.1966c	**3,7**	4,3	61,7	**0,00**		periplasmic sugar-binding protein
SMU.1967	**52,8**	215,1	233,1	**0,00**		single-stranded DNA-binding protein
SMU.1968c	-1,2	-2,5	1,1	0,86	*	hypothetical protein
SMU.1969c	-1,1	-2,6	1,2	0,48	*	transcriptional regulator
SMU.1970c	-1,1	x	1,5	**0,04**		phenylalanyl-tRNA synthetase subunit beta
SMU.1971c	-1	x	2,4	**0,00**		thioredoxin
SMU.1972c	-1,1	x	2,3	**0,00**		hypothetical protein
SMU.1973	-1,2	x	-2,6	**0,00**		glutamyl-aminopeptidase; endo-1,4-beta-glucanase
SMU.1974	-1,4	x	-2,7	**0,00**		pyrroline-5-carboxylate reductase
SMU.1975c	1,6	2,4	4,9	**0,00**		hypothetical protein
SMU.1976c	1,6	2,6	5,4	**0,00**		hypothetical protein
SMU.1977c	1,9	2,8	4,9	**0,00**		transcriptional regulator
SMU.1978	**7,5**	17,9	24,4	**0,00**		acetate kinase
SMU.1979c	**29,8**	92,5	209,9	**0,00**		hypothetical protein
SMU.1980c	**102,3**	1618,5	473,1	**0,00**		hypothetical protein
SMU.1981c	**68,6**	936,7	469,8	**0,00**		hypothetical protein
SMU.1982c	**111,6**	715,7	304,2	**0,00**		hypothetical protein
SMU.1983	**68,8**	408,9	450,4	**0,00**		competence protein ComYD
SMU.1984	**70**	1185,2	262,3	**0,00**		competence protein ComYC
SMU.1985	**69,8**	783	343,8	**0,00**		ABC transporter ComYB
SMU.1987	**106**	625,8	353,0	**0,00**		ABC transporter ATP-binding protein ComYA; late competence gene
SMU.1988c	1	x	2,1	**0,00**		DNA binding protein
SMU.1989	1,2	x	1,0	0,97		DNA-directed RNA polymerase subunit beta'
SMU.1990	1,2	x	1,0	0,96		DNA-directed RNA polymerase subunit beta
SMU.1991	1,1	x	-1,0	0,81		membrane carboxypeptidase, penicillin-binding protein 1b
SMU.1992	-1,2	x	-1,4	0,06		tyrosyl-tRNA synthetase
SMU.1993	-1,1	x	-1,1	0,84	*	ABC transporter zinc permease
SMU.1994	1	x	1,4	0,16		ABC transporter ATP-binding protein
SMU.1995c	1,1	3,1	1,6	0,10	*	transcriptional regulator
SMU.1996	1,6	2,5	4,4	**0,00**		4-diphosphocytidyl-2-C-methyl-D-erythritol kinase
SMU.1997	**25,7**	61,5	45,9	**0,00**		ComX1, transcriptional regulator of competence-specific genes
SMU.1999c	-1,3	x	-1,1	0,73		hypothetical protein
SMU.2000	-1,1	x	-1,3	0,16		50S ribosomal protein L17
SMU.2001	-1,2	x	-1,3	0,13		DNA-directed RNA polymerase subunit alpha
SMU.2002	-1,1	x	-1,3	0,11		30S ribosomal protein S11
SMU.2003	-1,1	x	-1,4	0,06		30S ribosomal protein S13
SMU.2003a	-1,2	x	-1,4	0,06		50S ribosomal protein L36
SMU.2004	-1,2	x	-1,5	**0,03**		translation initiation factor IF-1
SMU.2005	-1,1	x	-1,5	**0,03**		adenylate kinase
SMU.2006	-1,2	x	-1,4	0,07		preprotein translocase subunit SecY
SMU.2007	-1,1	x	-1,4	**0,05**		50S ribosomal protein L15
SMU.2008	-1,1	x	-1,4	**0,03**		50S ribosomal protein L30
SMU.2009	-1	x	-1,7	**0,00**		30S ribosomal protein S5
SMU.2010	-1	x	-1,5	**0,01**		50S ribosomal protein L18
SMU.2011	-1	x	-1,6	**0,00**		50S ribosomal protein L6
SMU.2012	-1,2	x	-1,8	**0,00**		30S ribosomal protein S8
SMU.2014	-1,1	x	-1,7	**0,00**		30S ribosomal protein S14
SMU.2015	-1,3	x	-1,6	**0,00**		50S ribosomal protein L5
SMU.2016	-1,3	x	-1,8	**0,00**		50S ribosomal protein L24
SMU.2017	-1,1	x	-1,6	**0,01**		50S ribosomal protein L14

SMU.2018	-1,2	x	-1,7	**0,00**		30S ribosomal protein S17
SMU.2019	-1,1	x	-1,6	**0,00**		50S ribosomal protein L29
SMU.2020	-1,1	x	-2,0	**0,00**		50S ribosomal protein L16
SMU.2021	-1,1	x	-2,1	**0,00**		30S ribosomal protein S3
SMU.2022	-1,3	x	-2,0	**0,00**		50S ribosomal protein L22
SMU.2023c	-1,1	x	-1,9	**0,00**		30S ribosomal protein S19
SMU.2024c	-1,1	x	-1,5	**0,02**		50S ribosomal protein L4
SMU.2025	-1,1	x	-1,6	**0,00**		50S ribosomal protein L3
SMU.2026c	-1,1	x	-1,3	0,14		30S ribosomal protein S10
SMU.2027	1,2	x	1,5	**0,02**		transcriptional regulator
SMU.2028	-1,1	4,2	-1,2	0,40		beta-D-fructosyltransferase
SMU.2029	-1,1	x	-1,1	0,72		class III stress response-related ATP-dependent Clp protease, ATP-binding subunit
SMU.2030	1	x	-1,2	0,41		CtsR family transcriptional regulator
SMU.2031	-1,1	x	-1,2	0,25		elongation factor Ts
SMU.2032	-1,1	x	-1,2	0,20		30S ribosomal protein S2
SMU.2033c	-1,3	x	-1,1	0,48		hypothetical protein
SMU.2035	-1,1	x	1,2	0,22		bacteriocin immunity protein
SMU.2036	-1	x	-1,2	0,41		peptidase
SMU.2037	1,3	x	-1,5	0,08		trehalose-6-phosphate hydrolase TreA
SMU.2038	1,2	x	-1,4	0,22		PTS system trehalose-specific transporter subunit IIABC
SMU.2040	1	x	1,4	0,26	*	transcriptional regulator; repressor of the trehalose operon
SMU.2042	-1,1	x	-1,5	**0,02**	*	dextranase
SMU.2043c	-1,1	x	-1,4	0,09	*	D-tyrosyl-tRNA(Tyr) deacylase
SMU.2044	-1,2	x	-1,3	0,17		stringent response protein, ppGpp synthetase
SMU.2046c	1	x	-1,0	0,90		hypothetical protein
SMU.2047	1	x	-1,4	0,05		PTS system glucose-specific transporter subunit IIABC
SMU.2048	1	x	6,4	0,17		hypothetical protein
SMU.2049c	1,1	x	1,1	0,84		16S ribosomal RNA methyltransferase RsmE
SMU.2050c	1,2	x	1,0	0,88	*	50S ribosomal protein L11 methyltransferase
SMU.2052c	1	x	1,2	0,60		hypothetical protein
SMU.2053c	-1,3	2,4	1,5	0,48		hypothetical protein
SMU.2054c	1,2	x	1,2	0,44	*	hypothetical protein
SMU.2055	-1	x	-1,1	0,63	*	acetyltransferase
SMU.2056	1,1	x	-1,2	0,25		recombination factor protein RarA
SMU.2057c	1,3	x	2,4	**0,05**		cadmium-transporting ATPase
SMU.2058	-1,1	x	-1,0	0,97		transcriptional regulator
SMU.2059c	-1,1	x	-1,3	0,25		hypothetical protein
SMU.2060	-1	x	1,0	0,95	*	LysR family transcriptional regulator
SMU.2061	-1	x	1,0	0,81		hypothetical protein
SMU.2063	-1	x	-1,4	0,08		ferrochelatase
SMU.2064c	-1,1	x	-1,4	0,07		transmembrane protein
SMU.2065	-1,1	x	-1,3	0,11		UDP-glucose 4-epimerase
SMU.2066c	-1,1	x	-1,3	0,12		transmembrane protein
SMU.2067	-1	x	-1,3	0,23		stress response protein
SMU.2069	-1,1	x	1,3	0,51		zinc transporter ZupT
SMU.2070	1	x	1,2	0,75		hypothetical protein
SMU.2071	-1	x	1,4	0,07	*	anaerobic ribonucleotide reductase activating protein
SMU.2072c	-1	x	1,3	0,26	*	acetyltransferase
SMU.2073c	1	x	1,4	0,19		hypothetical protein
SMU.2074	-1	x	1,1	0,49		anaerobic ribonucleoside triphosphate reductase
SMU.2075c	1,8	x	4,1	**0,00**		hypothetical protein
SMU.2076c	**4,2**	6,5	23,7	**0,00**		hypothetical protein
SMU.2077c	1,1	x	2,3	**0,00**		hypothetical protein
SMU.2078c	1,3	x	2,8	**0,00**	*	Holliday junction resolvase-like protein

SMU.2079c	1,3	x	2,8	**0,00**		hypothetical protein
SMU.2080	-1,1	5	1,4	0,64		hypothetical protein
SMU.2081	-1	4,2	1,1	0,85		hypothetical protein
SMU.2083c	1,8	3,1	5,3	**0,00**	*	hypothetical protein
SMU.2084c	1,9	2,9	5,6	**0,00**	*	transcriptional regulator Spx
SMU.2085	**4,6**	6,5	14,7	**0,00**		recombinase A
SMU.2086	**8**	10	27,0	**0,00**		competence damage-inducible protein A
SMU.2087	-1,1	x	1,1	0,72		3-methyladenine DNA glycosylase
SMU.2088	-1,1	x	-1,1	0,48		Holliday junction DNA helicase RuvA
SMU.2089	-1,1	x	1,1	0,65		DNA mismatch repair protein
SMU.2090c	1,3	x	1,1	0,67		hypothetical protein
SMU.2091c	1,1	x	1,2	0,21		DNA mismatch repair protein MutS
SMU.2092c	1,1	x	1,1	0,55		hypothetical protein
SMU.2093	1	x	1,1	0,59		transcriptional regulator of arginine metabolism
SMU.2094c	-1,1	x	1,9	0,08		hypothetical protein
SMU.2096c	-1	x	2,6	0,06		hypothetical protein
SMU.2097	-1,1	x	1,0	0,98	*	hypothetical protein
SMU.2098	-1	x	-1,2	0,25	*	arginyl-tRNA synthetase
SMU.2099c	1	x	-1,2	0,31		hypothetical protein
SMU.2100c	-1,1	x	-1,3	0,20		hypothetical protein
SMU.2101	-1	x	-1,3	0,19		aspartyl-tRNA synthetase
SMU.2102	-1,1	x	-1,3	0,22		histidyl-tRNA synthetase
SMU.2104	-1,1	x	-1,1	0,43		hypothetical protein
SMU.2104a	-1,2	x	-1,2	0,21		50S ribosomal protein L32
SMU.2105	-1,1	x	-1,2	0,53		hypothetical protein
SMU.2106c	1,1	x	1,0	0,97		transcriptional regulator
SMU.2107c	1	x	-1,3	0,50		hypothetical protein
SMU.2108c	-1,1	x	-1,2	0,63	*	transcriptional regulator
SMU.2109	-1,2	x	1,1	0,55	*	MDR permease
SMU.2111c	1,1	x	-1,7	0,17	*	hypothetical protein
SMU.2112	-1,1	x	-2,2	**0,00**		glucan-binding protein GbpA
SMU.2113c	-1,1	x	-1,2	0,57	*	hypothetical protein
SMU.2114c	-1,1	x	1,1	0,89		transcriptional regulator
SMU.2115	-1	x	-1,3	0,69	*	short-chain dehydrogenase
SMU.2116	-1,1	-2,1	-1,2	0,31	*	osmoprotectant amino acid ABC transporter ATP-binding protein
SMU.2117	-1	x	-1,2	0,42	*	osmoprotectant ABC transporter permease
SMU.2118	-1,1	x	-1,0	0,98		ABC transporter glycine betaine/carnitine/choline-binding protein
SMU.2119	-1,1	x	1,0	0,90		osmoprotectant ABC transporter permease
SMU.2120c	1,1	x	1,0	0,99		3-methyladenine DNA glycosylase
SMU.2121c	-1,2	x	1,3	0,42		hypothetical protein
SMU.2123	-1	x	-1,1	0,90		hypothetical protein
SMU.2124	-1,2	x	1,3	0,62	*	hypothetical protein
SMU.2125	-1	x	-1,2	0,63	*	hypothetical protein
SMU.2126c	1,1	x	1,5	0,09	*	purine-nucleoside phosphorylase
SMU.2127	-1	x	-1,2	0,19	*	succinate semialdehyde dehydrogenase
SMU.2128	1	x	-1,3	0,17		dihydroxy-acid dehydratase
SMU.2129c	-1,1	x	-1,1	0,64		hypothetical protein
SMU.2130	-1,1	x	-1,0	0,97		hypothetical protein
SMU.2131	-1,3	x	-1,4	0,73	*	hypothetical protein
SMU.2133c	1	x	2,4	**0,00**	*	hypothetical protein
SMU.2134	1,1	x	1,0	0,98	*	transcriptional regulator
SMU.2135c	-1	x	1,0	0,93		30S ribosomal protein S4
SMU.2136c	-1,2	x	-1,1	0,60	*	hypothetical protein
SMU.2137c	-1,2	x	-1,1	0,60		hypothetical protein

SMU.2138	-1	x	-1,0	0,92		replicative DNA helicase
SMU.2139c	-1,3	x	1,0	0,75		50S ribosomal protein L9
SMU.2142	-1,1	3,3	1,3	0,20		hypothetical protein
SMU.2143c	-1,1	2,6	1,3	0,11		tRNA-specific 2-thiouridylase MnmA
SMU.2146c	-1	x	-1,8	0,08		hypothetical protein
SMU.2147c	1,2	x	-1,5	0,16		hypothetical protein
SMU.2148c	-1,2	x	-1,5	**0,05**		cobalt permease
SMU.2149c	-1,2	x	-1,3	0,20		cobalt transporter ATP-binding subunit
SMU.214c	-1,2	2	1,1	0,83		hypothetical protein
SMU.2150c	-1,2	x	-1,3	0,11		cobalt transporter ATP-binding subunit
SMU.2152c	-1,1	x	-1,2	0,21		hypothetical protein
SMU.2153c	-1,3	x	1,0	0,78		peptidase
SMU.2154c	-1,2	x	1,6	0,09		peptidase
SMU.2155	-1,2	x	1,3	0,23	*	hypothetical protein
SMU.2156	-1,2	x	1,3	0,12		recombination protein F
SMU.2157	-1	x	-1,8	**0,00**		inosine 5'-monophosphate dehydrogenase
SMU.2158c	-1	x	-1,3	0,14		tryptophanyl-tRNA synthetase
SMU.2159	-1,1	x	1,0	0,89		ABC transporter ATP-binding protein
SMU.215c	-1,2	2,2	1,3	0,32		hypothetical protein
SMU.2160	-1,1	2	-1,1	1,00		transmembrane protein
SMU.2162c	-1	x	-1,4	0,33		rRNA large subunit methyltransferase
SMU.2164	-1,1	x	-1,2	0,38	*	serine protease HtrA
SMU.2165	-1,3	x	1,0	0,95	*	SpoJ
SMU.2166	-1,2	x	-1,7	**0,00**		50S ribosomal protein L23
SMU.2167	-1,1	x	-1,7	**0,00**		50S ribosomal protein L2

1 Khan, R. *et al.* Comprehensive Transcriptome Profiles of Streptococcus mutans UA159 Map Core Streptococcal Competence Genes. *mSystems* **1**, doi:10.1128/mSystems.00038-15 (2016).

2 Wenderska, I. B. *et al.* Transcriptional Profiling of the Oral Pathogen Streptococcus mutans in Response to Competence Signaling Peptide XIP. *mSystems* **2**, doi:10.1128/mSystems.00102-16 (2017).
